# Halogen-Bonded Guanine Base Pairs, Quartets and Ribbons

**DOI:** 10.3390/ijms21186571

**Published:** 2020-09-08

**Authors:** Nicholas J. Thornton, Tanja van Mourik

**Affiliations:** EaStCHEM School of Chemistry, University of St Andrews, North Haugh, St Andrews KY16 9ST, UK; njthornton2@gmail.com

**Keywords:** guanine–guanine base pair, guanine ribbons, guanine quartet, density functional theory, dispersion correction

## Abstract

Halogen bonding is studied in different structures consisting of halogenated guanine DNA bases, including the Hoogsteen guanine–guanine base pair, two different types of guanine ribbons (R-I and R-II) consisting of two or three monomers, and guanine quartets. In the halogenated base pairs (except the Cl-base pair, which has a very non-planar structure with no halogen bonds) and R-I ribbons (except the At trimer), the potential N-X•••O interaction is sacrificed to optimise the N-X•••N halogen bond. In the At trimer, the astatines originally bonded to N1 in the halogen bond donating guanines have moved to the adjacent O6 atom, enabling O-At•••N, N-At•••O, and N-At•••At halogen bonds. The brominated and chlorinated R-II trimers contain two N-X•••N and two N-X•••O halogen bonds, whereas in the iodinated and astatinated trimers, one of the N-X•••N halogen bonds is lost. The corresponding R-II dimers keep the same halogen bond patterns. The G-quartets display a rich diversity of symmetries and halogen bond patterns, including N-X•••N, N-X•••O, N-X•••X, O-X•••X, and O-X•••O halogen bonds (the latter two facilitated by the transfer of halogens from N1 to O6). In general, halogenation decreases the stability of the structures. However, the stability increases with the increasing atomic number of the halogen, and the At-doped R-I trimer and the three most stable At-doped quartets are more stable than their hydrogenated counterparts. Significant deviations from linearity are found for some of the halogen bonds (with halogen bond angles around 150°).

## 1. Introduction

The last two decades have seen a huge upsurge in studies on halogen bonding, with overview articles and reviews also appearing in the literature [1,2,3,4,5,6,7]. A halogen bond is a type of σ-hole interaction, where a nucleophile interacts with the positively charged region (dubbed the σ-hole) at the extent of the R–X bond, where X is the halogen and R is typically C but can also be another atom. Usually, the halogen is covalently bonded to one atom, but it has been shown that halogen-bonded complexes can also be formed with molecules where the halogen is bonded to two carbon atoms [8]. Halogen bonding plays important roles in many aspects of chemistry and biology, including material science [9], molecular crystals [10], biological molecules [11], and molecular recognition [12].

Halogen bonding has promising applications in synthetic DNA. As the four canonical DNA bases, adenine (A), guanine (G), cytosine (C), and thymine (T), are the “letters” of the “genetic alphabet”, incorporating halogenated bases could extend this alphabet, which may allow engineering of novel artificial proteins or nucleic acids. However, not many studies exist on halogen bonding in nucleic acid structures. A density functional theory study at the B3LYP [13,14,15] level has shown that modified DNA bases can form halogen-bonded base pairs [16]. The authors considered canonical adenine–thymine (AT) and guanine–cytosine (GC) base pairs with either all or one of the hydrogens involved in hydrogen bonding replaced by Cl, Br, or I. They found that nearly all of the substituted base pairs form co-planar structures with favourable interaction energies. Another computational study on halogen bonds in nucleic acids using the ONIOM (Our own N-layered Integrated molecular Orbital and Molecular mechanics) method [17] considered C-Br•••O-P contacts [18]. Frontera and Bauzá showed through a PDB (protein data bank [19]) survey coupled with MP2 (second-order Møller–Plesset perturbation theory [20]) calculations that halogenated nucleic acids can form halogen bonds with protein residues [21]. Other PDB surveys considered halogen bonding in biomolecules including nucleic acids [11,22]. An experimental study demonstrated that a halogen bond formed between a brominated uracil and phosphate oxygen can be engineered in a four-stranded DNA junction [23]. In the current study, we investigate halogen bonding in guanine base pairs, quartets, and ribbons. The inspiration for this study came from a recent computational study by Paragi and Fonseca Guerra on the self-assembly of guanine nucleobases into quartet and ribbon structures [24]. The study relates to scanning tunnelling microscopy (STM) experiments where guanine molecules were shown to form tetramer ring structures on a gold surface; however, these ring structures rearranged into ribbon-like structures when heated up [25]. Two different ribbon patterns were observed: one where the ribbons contain an N-H•••N and an N-H•••O hydrogen bond between the guanine monomers (type I) and one where there are alternatively two N-H•••N hydrogen bonds or two N-H•••O hydrogen bonds between adjacent guanine monomers (type II) [26,27,28]. We were intrigued to see if these quartets and ribbons would still exist if the hydrogen-bonding hydrogens would be replaced by halogens. We consider the halogens Cl, Br, I, and At. We do not include F, as this halogen usually does not form halogen bonds [29]. This has been ascribed to the fluorine’s large electronegativity and tendency to engage in sp hybridisation, with a resulting influx of negative charge into the region where the positive σ-hole would be [4,30]. As in our previous studies on halogen bonding systems [8,31,32,33], we include the radioactive element At as it may help to reveal trends in energy and properties with increasing halogen size. Astatine is the most polarisable of the halogens considered here [34]. The first experimental evidence of halogen bonding with astatine was provided in 2018 [35]. In 2020, a coupled experimental and computational approach identified a 1:1 adduct of Bu_3_PO•••AtI as the strongest astatine-mediated halogen bond found so far [36].

## 2. Results and Discussion

### 2.1. Method Comparison

Table 1 compares the interaction energies and selected geometrical parameters calculated at different levels of theory for the I1,I2-substituted R-I dimer.

The dimer contains two potential halogen bonds: an N-I•••N interaction and an N-I•••O interaction (see Figure 2; upper part). Potential halogen bond distances and angles are included in Table 1 (R(I•••N)/∠(NI•••N) for the N-I•••N interaction and R(I•••O)/∠(NI•••O for the N-I•••O interaction). Further discussion on whether these are proper halogen bonds can be found in Section 2.3 below. There are some geometrical differences between the structures optimised with BLYP-D3 and B3LYP-D3 coupled with the def2-TZVP basis set. In particular, the R(I•••O) distance is shorter for the BLYP-D3/def2-SVP geometry optimisation. This is presumably due to the considerably larger BSSE, which brings the two monomers closer together. However, the effect on the interaction energy is negligible when computed using the TZVP level and we therefore decided to optimise structures with BLYP-D3/def2-SVP and calculate interaction energies with BLYP-D3/def2-TZVP.

### 2.2. Hoogsteen Base Pair

**Figure 1:**
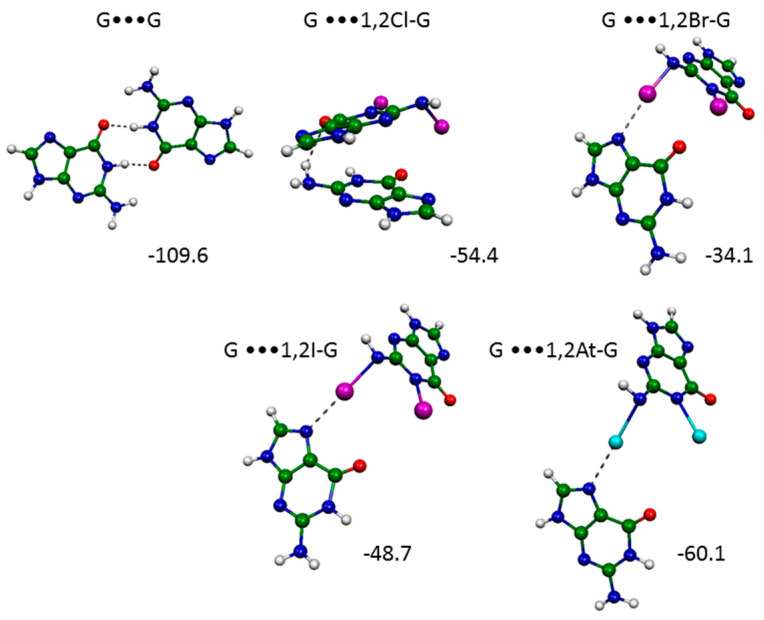
Optimised structures of the undoped and doped G•••1,2X-G (X = F, Cl, Br, I, At) base pairs starting from Hoogsteen structures, with their interaction energies (in kJ/mol).

Figure 1 shows the optimised structures of the G•••1,2X-G HS base pairs. The undoped HS base pair is not stable at the level of theory employed; it optimised to a C_2_-symmetric guanine base pair with N-H•••O hydrogen bonds (see Figure 1). The halogenated base pairs all have C_1_ symmetry. The Cl-doped base pair is very non-planar. It contains an N-H•••O hydrogen bond but no halogen bonds. The dimers with the heavier halogens are also non-planar but show potential halogen bonds. Their structures retain the Hoogsteen bonding pattern. All have interaction energies smaller than that for the corresponding undoped base pair. Table 2 lists the potential halogen bond distances and angles, as well as their vdW ratios. The vdW ratios are well below 1 for the three X•••N interactions (X = Br, I, At), just below 1 for the Br•••O interaction, and above 1 for the I•••O and At•••O interactions. This correlates with the nearly linear ∠(NX•••N) and non-linear ∠(NX•••O) angles. The very non-linear ∠(NX•••O) angles indicate that the N-X•••O interaction cannot be classified as a halogen bond in any of these base pairs. Thus, it appears that the structure of the halogenated base pairs results from optimisation of the N-X•••N halogen bond at the expense of the N-X•••O interaction. Optimisation of one halogen bond at the expense of another was also observed for the halogenated AT and CG base pairs [16]. The interaction energies of the Br- and I-doped base pairs (−34.1 and −48.7 kJ/mol, respectively) are in the same order of magnitude as those of Parker et al.’s AT base pair with two hydrogens replaced by Br or I (−39.8 and −28.3 kJ/mol, respectively) [16]. However, in our case, the I-substituted base pair is more stable than the Br-substituted base pair.

### 2.3. Ribbons R-I

**Figure 2:**
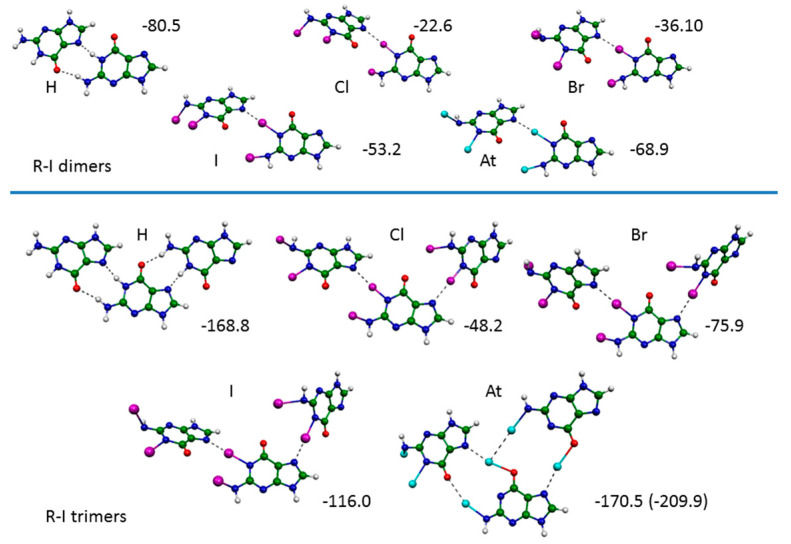
Optimised structures of the 1,2X-G•••1,2X-G R-I dimers and trimers (X = H, Cl, Br, I, At), with their interaction energies (in kJ/mol). The interaction energy in brackets for the At-substituted trimer is calculated with respect to two 1,6At-G monomers and one 1,2At-G monomer.

The R-I dimers and trimers are shown in Figure 2. Selected geometrical parameters and vdW ratios are given in Table 3. The unsubstituted dimer is a C_2_-symmetric reverse Hoogsteen base pair. It contains an N-H•••N and N-H•••O hydrogen bond. Bhattacharyya et al. calculated an interaction energy of −65.0 kJ/mol for this base pair with HF/6-31G(d,p) [37] and −73.6 kJ/mol at the B3LYP/6-31G(2d,2p) level [38], both smaller than the −80.5 kJ/mol calculated by us. This is presumably due to the missing correlation in the HF calculations and B3LYP’s inability to account for dispersion effects [39]. Reverse Hoogsteen G-G and A-A base pairs were found to occur in homo-DNA (a DNA homologue with the standard 2′-deoxyribofuranose replaced by 2′,3′-dideoxyglucopyranose) [40,41]. Doping with halogens makes the dimers non-planar and reduces the symmetry to C_1_. Like for the halogenated HS base pairs, the potential N-X•••O halogen bond has been sacrificed to optimise the N-X•••N halogen interaction, which is a proper halogen bond in all substituted dimers (vdW ratios below 0.8; ∠(NX•••N) angles 177–179°). As for the HS base pair, the interaction energy of the Br-doped dimer (−36.1 kJ/mol) is smaller, and that of the I-doped dimer larger (−53.2 kJ/mol), than those of the AT base pair with two Br or I atoms, respectively [16]. The addition of a third guanine monomer leaves the geometry of the dimer unit qualitatively unchanged for the undoped trimer and the trimers with Cl, Br, and I halogens (Table 3), with the Cl-, Br-, and I-doped trimers containing two N-X•••N and no N-X•••O halogen bonds. In the At-substituted trimer, however, the halogen bond pattern has changed. One of the astatines in the right and middle guanine moved from N1 to O6, creating two O-X•••N halogen bonds (vdW ratios 0.73 and 0.69 and ∠(OX•••N) angles 170 and 168°) instead of N-X•••N halogen bonds. The change in halogen bonding site in the right-most guanine molecule also allows an additional N-X•••X halogen bond (vdW ratio 0.83; angle 177°) and the change from N-X•••N to O-X•••N halogen bond in the left dimer allows the formation of an N-X•••O halogen bond (vdW ratio 0.78; angle 161°). The interaction energies of the substituted trimers increase upon increasing the atomic number of the halogen. For the At-substituted trimer, we also calculated the interaction energy with respect to the constituent monomers, i.e., two 1,6-At-G and one 1,2-At-G molecules (value in brackets in Figure 2). This value shows how strongly the bases interact but ignores the energy required to deform two 1,2-At-G to 1,6-At-G molecules. For all trimers, the interactions are cooperative: E_coop_ is −7.9, −2.9, −3,7, −9.6, and −32.7 kJ/mol for the undoped, Cl-, Br, I-, and At-doped trimers, respectively (calculated using the interaction energies with respect to 1,2-X-G monomers). The large value for the At-substituted trimer shows a large increase in stability due to the additional halogen bonds facilitated by the transfer of two hydrogens from N1 to O6.

### 2.4. Ribbons R-II

**Figure 3:**
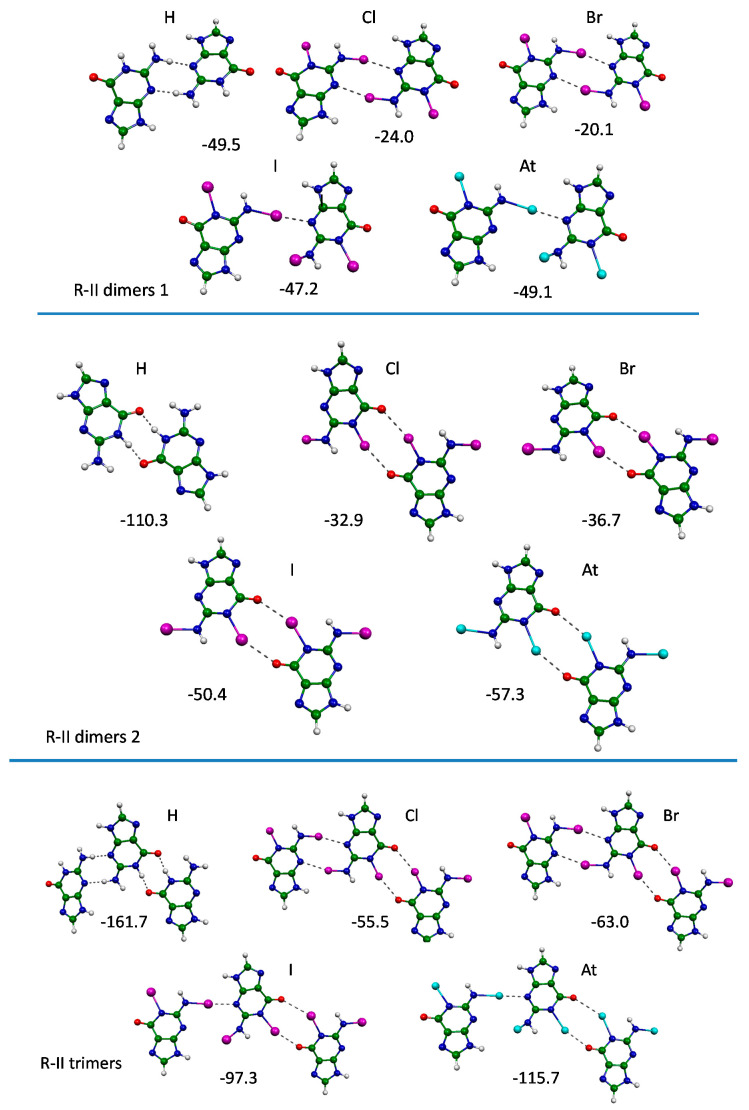
Optimised structures of the 1,2X-G•••1,2X-G ribbon II dimers and trimers (X = H, Cl, Br, I, At), with their interaction energies (in kJ/mol).

Figure 3 shows the optimised R-II dimers and trimers, whereas selected geometrical parameters and vdW ratios have been collected in Table 4. In contrast to the R-I trimers, the R-II trimers contain two different constituent dimers: one containing two N-X•••N interactions (dimer 1) and one with two N-X•••O interactions (dimer 2). The R-II trimers were first optimised; subsequently, starting structures were obtained for the geometry optimisations of the dimers by removing one of the outermost guanines. The undoped dimer 1 has C_2_ symmetry. It is non-planar, with the guanines twisted out of plane. It is the least stable of the three different guanine–guanine dimers considered in this work. Bhattacharyya et al. calculated an interaction energy of −34.0 kJ/mol for this base pair, at the B3LYP/6-31G(2d,2p) level [38], again smaller than the −49.5 kJ/mol calculated by us. The Cl-dimer has C_i_ symmetry. It is also non-planar, with a slight twist of one guanine accompanied by an opposite twist of the second guanine. The Br-dimer is planar with C_2h_ symmetry. The I- and At-dimers have no symmetry elements. In the latter two, one N-X•••N interaction is optimised at the expense of the second one. For the R-II dimer 2, the undoped, I-, and At-substituted dimers have C_2_ symmetry, whereas the Cl- and Br-substituted dimers have C_i_ symmetry. The undoped dimer is a reverse Watson–Crick base pair. Such base pairs occur in RNA, contributing to its structural complexity [42]. Mitra et al. calculated an interaction energy of −107.9 kJ/mol for the reverse Watson–Crick GG base pair (with methyl groups at the 9-position) at the M05-2X/6-31G++(2d,2p) level of theory [43], close to our result of −110.3 kJ/mol. All dimers 2 contain two symmetry-related N-X•••O halogen bonds. Again, the interaction energy of the Br-doped dimers (both 1 and 2) is smaller, and those of the I-doped dimers larger, than those of the AT base pair with two Br or I atoms, respectively [16]. The symmetry elements in the dimer disappear in the trimer. The undoped, Cl-, and Br-containing trimers have clearly two N-X•••N and two N-X•••O halogen bonds. Like for the dimer 1, in the I- and At-trimer, one of the N-X•••N halogen bonds is sacrificed to optimise the other N-X•••N halogen bond. Unlike for the ribbons R-I, the interactions in the trimers are not always cooperative: E_coop_ is −1.9, 1.4, −6.1, 0.3, and −9.4 kJ/mol for the undoped, Cl-, Br-, I-, and At-containing trimers, respectively.

### 2.5. G-Quartets

**Figure 4:**
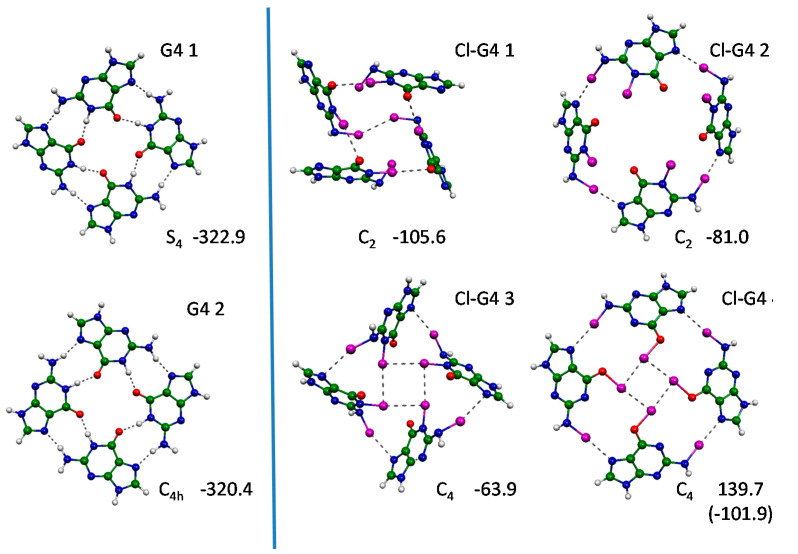

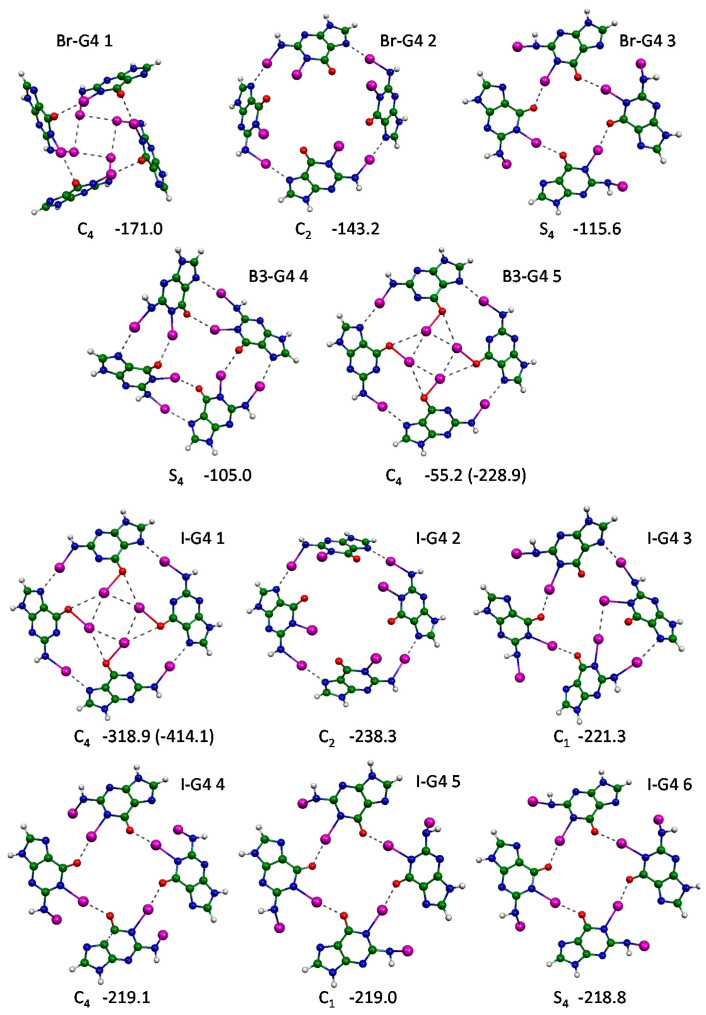

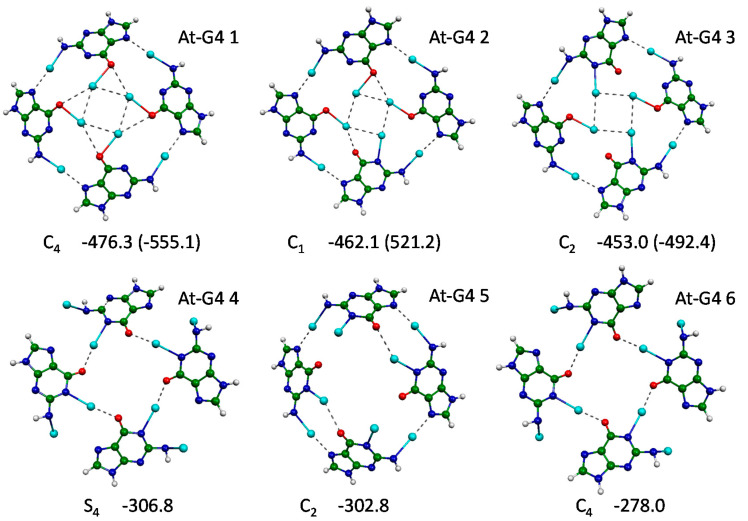
The optimised X-quartets (X = Cl, Br, I, At). The symmetry and interaction energy (in kJ/mol) are listed below the structure. The interaction energy in brackets is calculated with respect to four 1,6X-G monomers (for Cl-G4 4, Br-G4 5, and At-G4 1), three 1,6X-G monomers, and one 1,2X-G monomer (At-G4 2) or two 1,6X-G monomers and two 1,2X-G monomers (At-G4 3).

G-quartets are structural motifs occurring in G-quadruplexes, which are higher-order DNA and RNA structures formed by sequences that are rich in guanine [44]. G-quartets consist of four guanine bases, interacting through Hoogsteen base pairing. Figure 4 shows the optimised structures of the 1,2X-G quartets. Some selected geometrical parameters are available in the Supplementary Materials (Table S1).

We located two structures for the undoped G-quartet: an S_4_-symmetric quartet and a C_4h_-symmetric quartet, both containing Hoogsteen GG base-pair hydrogen bonds. The S_4_ quartet is slightly more stable than the C_4h_ quartet, in agreement with previous results [45,46]. The absence of imaginary frequencies indicates that the S_4_-symmetric quartet is a true minimum on the potential energy surface. We find one imaginary frequency for the C_4h_ quartet, in agreement with previous calculations at the B3LYP/6-311G(d,p) level [46] but in disagreement with the B3LYP/DZVP results of Meyer et al. [45], which found four imaginary frequencies. Unlike the previous studies, no quartet with bifurcated hydrogen bonds was located. Attempts to optimise such a structure resulted in the S_4_-symmetric quartet. Paragi and Fonseca Guerra calculated an interaction energy of −331 kJ/mol with BLYP-D and a basis set of triple-zeta quality [24], somewhat larger than our value of 320–322 kJ/mol. However, Paragi and Fonseca Guerra’s result is not corrected for BSSE. We calculate BSSE values of −12 to −13 kJ/mol for the unsubstituted quartets, which accounts for most of the difference between our results and those of Paragi and Fonseca Guerra. It should also be noted that Paragi and Fonseca Guerra optimised the quartets with planar constraint to mimic the adsorption onto a gold surface.

For the Cl-doped quartet, two C_2_-symmetric and two C_4_-symmetric structures were located. The most stable quartet (ΔE^CP^ = −105.6 kJ/mol) is a C_2_-symmetric structure with a central Cl•••Cl halogen bond (vdW ratio 0.88; but with ∠N-Cl•••Cl angles of 148°, on the border of being true halogen bonds) and four (two-by-two identical) N-Cl•••N halogen bonds (vdW ratios of 0.87 and 0.88; angles 166 and 156°, respectively). The next quartet (ΔE^CP^ = −81.0 kJ/mol) has also C_2_ symmetry, and contains four (two-by-two symmetric) N-Cl•••N halogen bonds (all vdW ratios 0.76; angles 176 and 179°). It has a slight “bowl” structure. The Cl•••O distances are too large to classify the N Cl•••O interactions as halogen bonds. The third most stable Cl-quartet (ΔE^CP^ = −63.9 kJ/mol) is C_4_-smmetric. It has four symmetry-related N-Cl•••N halogen bonds (vdW ratio 0.87; angle 163°). Four Cl atoms form a square in the central cavity linked by Cl•••Cl halogen bonds (vdW ratio 0.90; ∠N-Cl•••Cl 174°). The fourth Cl-quartet has a positive interaction energy when the energy of the 1,2Cl-G molecule is used as reference. In this quartet, the Cl atoms originally bonded to N1 have moved to the adjacent O6 oxygen. If the 1,6Cl-G monomer (with one Cl attached to O6) is used to calculate the interaction energy, it becomes negative (−101.9 kJ/mol; number in brackets in Figure 2), showing that the bases interact favourably.

The most stable Br-containing quartet is C_4_-symmetric, with N-Br•••O hydrogen bonds (vdW ratio 0.80; angles 166°) and a square of four bromines in the centre forming Br•••Br halogen bonds (vdW ratio 0.88; ∠N-Cl•••Br 176°). It is similar to the Cl-quartet 3. The second Br-quartet, which is similar to the Cl-quartet 2, has C_2_ symmetry and four N-Br•••N halogen bonds (vdW ratios 0.71 and 0.72; angles 179 and 176°). The four N-Br•••O contacts are not halogen bonds: two have a vdW ratio above 1 and, whereas the other two have vdW ratios below 1 (0.92), their N-Br•••O angles (101°) are too far from linearity for halogen bonds. The third Br-quartet is S_4_-symmetric; the guanine bases are slightly twisted out-of-plane. It contains four symmetry-related N-Br•••O halogen bonds (vdW ratios 0.73; angles 171°). The fourth Br-quartet is also S_4_-symmetric. It contains four N-Br•••N halogen bonds. The N-Br•••O contacts can just be considered proper halogen bonds with vdW ratios comfortably below 1 (0.81) but with rather non-linear N-H•••O angles (148°). The fifth Br-quartet has C_4_ symmetry. It contains four N-Br•••N halogen bonds (vdW ratio 0.77; angle 175°) and four bifurcated halogen bonds, consisting of one strong O-Br•••Br halogen bond (vdW ratio 0.77; angle 169°) and a weaker O-Br•••O halogen bond (vdW ratio 0.95; angle 150°). The bromine atoms have moved from N1 to O6. The structure is similar to Cl-quartet 4, except that, in the latter, the O-Cl•••O interactions cannot be considered halogen bonds because of their vdW ratios above 1 (1.14).

Like Br-quartet 5, the most stable I-quartet is C_4_-symmetric with four identical N-I•••N halogen bonds (vdW ratio 0.73; angle 174°) and bifurcated halogen bonds with a strong O-I•••I halogen bond (vdW ratio 0.78; angle 162) and weaker O-I•••O halogen bond (vdW ratio 0.93; angle 154°). Like Cl-quartet 4 and Br-quartet 5, the halogen has moved from N1 to the adjacent O6. The next I-quartet is a C_2_-symmetric bowl-like structure with four (two-by-two symmetry-related) N-I•••N halogen bonds (vdW ratios 0.70 for all four bonds; angles 179° and 177°). It is similar to Cl-quartet 2 and Br-quartet 2. The third I-quartet has no symmetry elements. Two of the N-I•••N interactions can be classified as halogen bonds (vdW ratios of 0.72 and 0.76; angles 170 and 168°). Moreover, two of the N-I•••O interactions are halogen bonds (vdW ratios 0.68 and 0.69; angles 178 and 174°), as well as one of the N-I•••N interactions (vdW ratio 0.81; angle 177°). The fourth I-quartet has C_4_ symmetry. It contains four N-I•••O halogen bonds (vdW ratios 0.68; angles 173°). The fifth most stable I-quartet has no symmetry elements. It contains four N-I•••O halogen bonds (vdW ratio 0.68–0.69; angle 173–175°). The sixth most stable I-quartet is S_4_-symmetric and contains four N-I•••O halogen bonds (vdW ratios 0.68; angles 173°). The last three I-quartets have similar energies; however, they differ slightly in structure. Whereas the iodines bonded to the amino group point alternatively up and down from the approximate plane of the molecule in the C_1_ and S_4_-symmetric structures, they point the same way in the C_4_-symmetric structure.

The most stable At-quartet is a bowl-shaped C_4_-symmetric structure with four N-At•••N halogen bonds (vdW ratio 0.72; angle 174°), four O-At•••O halogen bonds (vdW ratio 0.90; angle 157°), and four O-X•••X halogen bonds (vdW ratio 0.79; angle 157°). The inner astatine atoms moved from N1 to O6. It is similar to Cl-quartet 4 (even though this lacks proper O-Cl•••O halogen bonds), Br-quartet 5, and I-quartet 1. The next At-quartet does not have symmetry elements. It displays four N-At•••N halogen bonds (vdW ratios 0.71–0.72; angles 175–176°). If the distance from N1 or O6 to the halogen is used to determine which atom the astatine is bonded to, then, in three of the guanines, the inner At atom is bonded to O6, and in the fourth guanine, it is bonded to N1. There is a combination of N-At•••At, O-At•••At, and O-At•••O halogen bonds, as indicated in the figure. The third At-quartet is C_2_-symmetric, with four N-At•••N halogen bonds (vdW ratios 0.72–0.73; angles 178–177°), two N-At•••At halogen bonds (vdW ratio 0.80; angle 175°), and two O-At•••At halogen bonds (vdW ratios 0.81; angles 157°). The fourth At-quartet is S_4_-symmetric with four N-At•••O halogen bonds (vdW ratio 0.68; angle 173°). The fifth At-quartet has C_2_ symmetry; it contains four N-At•••N halogen bonds (vdW ratios 0.70–71; angles 179–174°) and two N-At•••O halogen bonds (vdW ratio 0.84; angle 161°). The last At-quartet is C_4_-symmetric with four N-At•••O halogen bonds (vdW ratio 0.69; angle 175°). At-quartets 4 and 6 have a similar structure; however, in the S_4_-symmetric structure, the astatines on the amino group are pointing alternatively up and down, whereas in the C_4_-symmetric structure, they point in the same direction.

Chlorination and bromination leads to less stable quartets. However, the most stable iodinated quartet has an interaction energy very close to the undoped quartet, and the three most stable astatinated quartets are more stable than the unsubstituted quartet.

## 3. Methodology

**Scheme 1:**
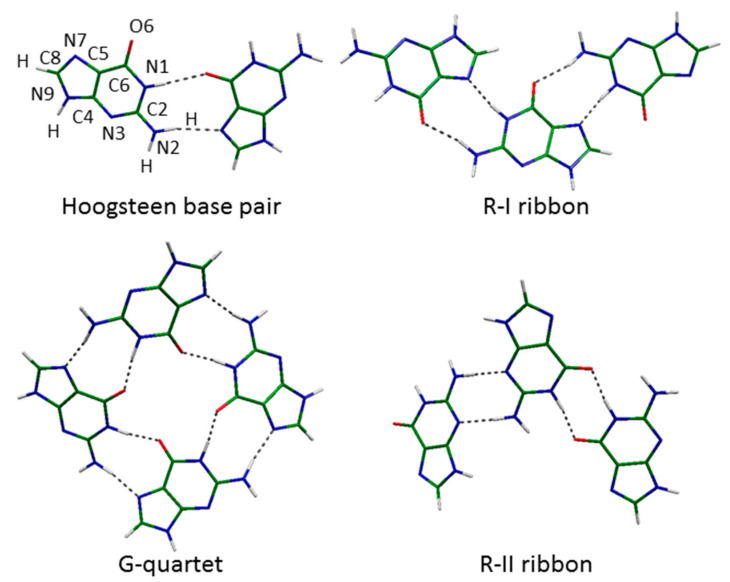
Guanine structures considered in this work. Atom labelling is shown for one of the guanines in the Hoogsteen base pair.

The structures considered include guanine–guanine (GG) base pairs with Hoogsteen (HS) base pairing, dimer and trimer ribbons with N-H•••N and N-H•••O hydrogen bonds between the monomers (R-I ribbons), dimer and trimer chains with two N-H•••N hydrogen bonds or two N-H•••O hydrogen bonds between the monomers (R-II ribbons), and G-quartets with Hoogsteen base pairing (see Scheme 1). Halogenated structures were created by replacing the hydrogens that form H-bonds in the GC base pair, i.e., the hydrogen attached to N1 and one of the hydrogens attached to N2 (see Scheme 1 for atom labelling) by a halogen (X = Cl, Br, I, or At). In the base pairs, only the hydrogens forming the hydrogen bonds were replaced; in all other structures, the corresponding hydrogens of all guanine substituents were replaced.

The structures were optimised using the BLYP density functional [13,15] augmented with a D3 dispersion term [47] with Becke–Johnson (BJ) damping [48,49], using the def2-SVP basis set [50]. We chose this functional in combination with the D3 dispersion term because the BLYP-D method showed excellent performance for systems of stacked nucleic acid base pairs and quartets [51]. Interaction energies, corrected for basis set superposition error (BSSE) using the counterpoise (CP) procedure [52], were computed at the BLYP-D3 level using the def2-TZVP basis set [50] (see Supplementary Materials Section S1 for details on the calculation of CP-corrected interaction energies and BSSE values). For the I1,I2-substituted R-I dimer, geometry optimisations were also carried out at the BLYP-D3/TZVP and B3LYP-D3/TZVP levels of theory. All calculations were done using the Orca 4.1.1 program [53,54] and used the def2/J auxiliary basis set [55] and integration Grid 7. The optimised structures are available in the Supplementary Materials (Table S2). Note that the def2 basis sets use the def2-ECP effective core potential for heavy atoms (in this case, iodine and astatine). In some cases, we encountered convergence problems with the SCF (self-consistent field), mainly during the CP calculations (see Supplementary Materials Section S2).

Criteria used to classify interactions as halogen bonds include (i) the requirement that the interatomic distance divided by the sum of the van der Waals radii [56,57] of the constituent atoms, labelled the vdW ratio, is smaller than 1, and (ii) that the halogen bond angle is “not too far away” from being linear. Though halogen bonds tend to be linear, in previous work we found that significantly non-linear halogen bonds (150–160°) may exist in complex environments with competing interactions [32,33]. Thus, we deem angles over 150° compatible with the interaction being a halogen bond.

Point group symmetry was determined using GaussView [58], in some cases using the loosest tolerance for detecting symmetry. For the trimers, cooperativity is defined as follows:

(1) $$E_{coop}=\Delta E_{trimer}^{CP}-\Delta E_{dimer1}^{CP}-\Delta E_{dimer2}^{CP}$$

where $\Delta E_{dimer1}^{CP}$ and $\Delta E_{dimer2}^{CP}$ are the interaction energies of the constituting dimers in the trimer, and the “CP” superscripts indicate that the interaction energies are counterpoise-corrected.

## 4. Conclusions

We investigated the effect on structure and stability of replacing the hydrogen-bonding hydrogens in various guanine-based structures with halogens. The systems studied include guanine–guanine base pairs (in the Hoogsteen form), two different types of ribbons (R-I and R-II) consisting of two and three guanines, and guanine quartets. In the base pairs and R-I ribbons, which contain potential N-X•••N and N-X•••O interactions, a trend was observed to optimise the N-X•••N halogen bond at the expense of the N-X•••O interaction. This is the case for the Br-, I-, and At-containing base pairs, all halogenated R-I dimers, and the Cl-, Br-, and I-containing R-I trimers. The Cl-doped base pair has a distorted structure with a hydrogen bond but no halogen bonds. The undoped Hoogsteen base pair is not stable on the BLYP/def2-SVP potential energy surface; however, doping with heavier halogens (Br, I, At) restores the Hoogsteen pattern. In the At-doped R-I trimer, the hydrogens originally attached to N1 in the two halogen bond donating monomer units have moved to the adjacent O6, facilitating O-At•••N instead of the N-At•••N halogen bonds as well as forming an additional N-At•••O and N-At•••At halogen bond. The R-II trimers contain two different dimers, with either two potential N-X•••N (dimers 1) or two potential N-X•••O interactions (dimers 2). The Cl- and Br-doped dimers 1 contain two equivalent N-X•••N halogen bonds, but in the I- and At-containing dimers, one of the interactions is lost. This is presumably due to the larger size of I and At, which makes it more difficult to accommodate both halogens in halogen-bonding positions. It is presumably more efficient to optimise one halogen bond instead. The dimers 2 all contain two equivalent N-X•••O halogen bonds. The halogen bond pattern is the same in the trimer as in its constituent dimers. The halogenated G-quartets display a range of halogen bond patterns. In some structures, the N-X•••N halogen bond is optimised at the expense of the N-X•••O interaction. However, in other structures, the N-X•••O halogen bond is optimised instead, whereas there are also structures where the N-X•••N and N-X•••O halogen bonds co-exist. Some structures manage to incorporate X•••X halogen bonds, either by adopting very non-planar structures or by moving the inner halogens from the N1 to the adjacent O6 atom. The latter structures become more important for the heavier atoms: whereas, for the Cl- and Br-quartets, this was the least stable structure, the most stable I- and At-quartet feature O6-X•••X halogen bonds. In fact, the three most stable At-quartets feature O6-X•••X halogen bonds. Our results indicate that halogenated bases can be incorporated into nucleic acid structures, which is relevant for the incorporation of unnatural halogenated bases into synthetic DNA and other nucleic acid structures.

In general, substitution of the hydrogen-bonding hydrogens by halogens leads to a decrease in stability, though the interaction energy of the At-doped R-I trimer just exceeds that of its hydrogen-containing counterpart, whereas the interaction energies of the three most stable At-doped quartets comfortably exceed that of the hydrogen-containing quartet. The At-doped R-II dimer 1 has an interaction energy nearly identical to the unsubstituted dimer (−49.1 vs. −49.5 kJ/mol). This shows that halogen bonds containing iodine or astatine can be similar, or stronger, in stability than corresponding hydrogen bonds. In all structures, the interaction energy increases with the increasing atomic number of the halogen. This is in disagreement with the results of Parker et al. for the canonical base pairs, where, in most cases, the Br-doped base pairs are more stable than the I-doped base pairs [16]. A recent study performing relativistic quantum calculations on complexes formed between halide anions and a series of Y_3_C-X (Y = F to X, X = I, At) halogen bond donors revealed the weaker donation ability of At_3_C-At compared to I_3_C-I [59]. However, in most cases reported in the literature, heavier halogens form stronger halogen bonds.

## Figures and Tables

**Table 1:** Interaction energies and BSSE (basis set superposition error) values (in kJ/mol) and selected geometrical parameters (distances in Å; angles in degrees) of the I1,I2-substituted R-I dimer, calculated at different levels of theory.

Geometry	Energy	ΔE^CP^	BSSE	R(I•••N)	R(I•••O)	∠(NI•••N)	∠(NI•••O)
BLYP-D3/SVP	BLYP-D3/SVP	−55.5	−13.5	2.58	3.78	178	113
BLYP-D3/SVP	BLYP-D3/TZVP	−53.2	−2.1				
BLYP-D3/TZVP	BLYP-D3/TZVP	−53.0	−2.0	2.61	3.94	177	110
BLYP-D3/SVP	B3LYP-D3/TZVP	−54.7	−1.8				
B3LYP-D3/TZVP	B3LYP-D3/TZVP	−53.2	−1.7	2.60	3.98	177	107

**Table 2:** Selected geometrical parameters (distances in Å; angles in degrees) and van der Waals ratios (vdW) for the G•••1,2X-G dimers (X = Br, I, At).

System	R(X•••N)	R(X•••O)	vdW(X•••N)	vdW(X•••O)	∠(NX•••N)	∠(NX•••O)
G•••1,2Br-G	2.59	3.26	0.76	0.97	173.1	137.2
G•••1,2I-G	2.60	3.99	0.74	1.14	179.5	122.3
G•••1,2At-G	2.63	4.34	0.74	1.23	179.4	117.8

**Table 3:** Selected geometrical parameters (distances in Å; angles in degrees) and van der Waals ratios (vdW) for the R-I dimers and trimers.

System	R(X•••N)	R(X•••O)	vdW(X•••N)	vdW(X•••O)	∠(NX•••N)	∠(NX•••O)
(1,2Cl-G)_2_	2.58	3.24	0.78	0.99	178	135
(1,2Br-G)_2_	2.57	3.47	0.76	1.03	179	124
(1,2I-G)3_2_	2.58	3.78	0.73	1.08	178	113
(1,2At-G)_2_	2.60	3.74	0.73	1.06	177	111
(1,2Cl-G)_3_	2.54/2.54	3.47/3.35	0.78/0.77	1.06/1.03	176/178	125/135
(1,2Br-G)_3_	2.52/2.51	3.45/3.34	0.74/0.74	1.02/0.99	178/178	124/126
(1,2I-G)3_3_	2.53/2.52	3.76/3.67	0.72/0.71	1.07/1.05	178/179	109/116
(1,2At-G)_3_	2.52/2.47	2.77/3.53	0.73/0.69	0.78/1.00	143/146	161/138

**Table 4:** Selected geometrical parameters (distances in Å; angles in degrees) and van der Waals ratios (vdW) for the R-II dimers and trimers.

System	R(X•••N)	R(X•••O)	vdW(X•••N)	vdW(X•••O)	∠(NX•••N)	∠(NX•••O)
Dimer 1						
(1,2Cl-G)_2_	2.83		0.86		166	
(1,2Br-G)_2_	2.85		0.84		163	
(1,2I-G)_2_	2.65/4.52		0.75/1.28		177/97	
(1,2At-G)_2_	2.55/4.19		0.71/1.17		178/91	
Dimer 2						
(1,2Cl-G)_2_		2.75		0.84		166
(1,2Br-G)_2_		2.79		0.83		161
(1,2I-G)_2_		2.94		0.84		155
(1,2At-G)_2_		2.86		0.81		153
(1,2Cl-G)_3_	2.86/2.81	2.72/2.77	0.87/0.85	0.83/0.85	165/167	167/165
(1,2Br-G)_3_	2.82/2.85	2.74/2.83	0.83/0.84	0.81/0.84	165/160	163/159
(1,2I-G)_3_	2.63/4.50	2.73/3.23	0.74/1.28	0.78/0.92	177/97	153/147
(1,2At-G)_3_	2.56/4.16	2.68/3.13	0.72/1.17	0.76/0.88	178/91	160/146

## Supplementary Materials

Supplementary Materials can be found at https://www.mdpi.com/1422-0067/21/18/6571/s1. Section S1: Calculation of CP-corrected interaction energies; Section S2: Conversion problems; Table S1: Selected geometrical and van der Waals ratios for the (1,2X-G)4 quartets (X = Br, I, At); Table S2: Cartesian coordinates of the undoped and halogenated reverse Hoogsteen base pairs, R-I and R-II ribbons, and G-quartets.
