# Supplementary material for: Halogen-Bonded Guanine Base Pairs, Quartets and Ribbons
Source: Int J Mol Sci. 2020 Sep 8;21(18):6571. doi: 10.3390/ijms21186571 (PMC7555031; doi:10.3390/ijms21186571)
Supplement: Supplementary file 1 [file ijms-21-06571-s001.pdf]

Supplementary Materials

Halogen-bonded guanine base pairs, quartets and ribbons

Nick J. Thornton and Tanja van Mourik

Section S1. Calculation of CP-corrected interaction energies

Section S2. Convergence problems

Table S1. Selected geometrical parameters (distances in Å; angles in degrees) and van der Waals ratios (vdW) for the $(1,2X\text{-}G)_4$ quartets (X = Br, I, At)

Table S2. Cartesian coordinates (in Å) of the undoped and halogenated reverse Hoogsteen base pairs, R-I and R-II ribbons and G-quartets, in xyz format.

Section S1. Calculation of CP-corrected interaction energies

The CP-corrected interaction energy for an n-mer G_n, consisting of n guanines G^1 to G^n, is calculated as follows:

$$\Delta E^{CP} = E^{CP} - \sum_{i=1}^{n} E_{G^i}^{\{G^i\}}(G^i) \qquad (1)$$

With the CP-corrected n-mer energy calculated as:

$$E^{CP} = E_{G_n}^{\{G_n\}}(G_n) - BSSE \qquad (2)$$

where

$$BSSE = \sum_{i=1}^{n} E_{G^i}^{\{G_n\}}(G_n) - \sum_{i=1}^{n} E_{G^i}^{\{G^i\}}(G_n) \qquad (3)$$

In these equations the subscripts denote the molecular system (The n-mer G_n or individual monomers G^i), the superscripts in curly brackets denote the basis set employed (*i.e.* the monomer basis set $\{G^i\}$ or the n-mer basis set $\{G_n\}$) and the attribute in round brackets indicates the geometry (*i.e.* the optimised monomer geometry (G^i) or the n-mer geometry (G_n)). With this definition of BSSE [1], BSSE values are negative. This is in accordance with the notion that BSSE is an (artificial) attraction.

[1] F.B. van Duijneveldt, J.G.C.M. van Duijneveldt-van de Rijdt, J.H. van Lenthe, State of the art in counterpoise theory, Chem. Rev. 94 (1994) 1873.

Section S2. Convergence problems

In some cases def2-TZVP the SCF did not converge, or converged to a clearly wrong energy, in single-point calculations. In most cases we could solve this by switching from the default DIIS to the second-order (SOSCF) or Newton Raphson (NRSCF) algorithm. In two cases we did not manage to solve the convergence problems. For the R-II I-doped trimer, one of the monomer+ghost CP calculations converged to a too high energy. Here, we calculated the BBSE using Gaussian 09 [2] with BLYP-D3/def2-TZVP and used this to calculate the CP-corrected interaction energy. The second problem case was the At-substituted quartet 6. Here the single-point def2-TZVP calculation of the quartet converged to a too high energy. We calculated the CP-corrected interaction energy of this quartet with Gaussian 09. The Gaussian calculations employed the “Ultrafine” integration grid.

[2] M.J. Frisch, G.W. Trucks, H.B. Schlegel, G.E. Scuseria, M.A. Robb, J.R. Cheeseman, G. Scalmani, V. Barone, B. Mennucci, G.A. Petersson, H. Nakatsuji, M. Caricato, X. Li, H.P. Hratchian, A.F. Izmaylov, J. Bloino, G. Zheng, J.L. Sonnenberg, M. Hada, M. Ehara, K. Toyota, R. Fukuda, J. Hasegawa, M. Ishida, T. Nakajima, Y. Honda, O. Kitao, H. Nakai, T. Vreven, J. Montgomery, J. A., J.E. Peralta, F. Ogliaro, M. Bearpark, J.J. Heyd, E. Brothers, K.N. Kudin, V.N. Staroverov, R. Kobayashi, J. Normand, K. Raghavachari, A. Rendell, J.C. Burant, S.S. Iyengar, J. Tomasi, M. Cossi, N. Rega, J.M. Millam, M. Klene, J.E. Knox, J.B. Cross, V. Bakken, C. Adamo, J. Jaramillo, R. Gomperts, R.E. Stratmann, O. Yazyev, A.J. Austin, R. Cammi, C. Pomelli, J.W. Ochterski, R.L. Martin, K. Morokuma, V.G. Zakrzewski, G.A. Voth, P. Salvador, J.J. Dannenberg, S. Dapprich, A.D. Daniels, Ö. Farkas, J.B. Foresman, J.V. Ortiz, J. Cioslowski, D.J. Fox, Gaussian 09, Gaussian, Inc., Wallingford, CT, 2009.

Table S1. Selected geometrical parameters (distances in Å; angles in degrees) and van der Waals ratios (vdW) for the $(1,2X\text{-}G)_4$ quartets (X = Br, I, At)

Quartet	R(X•••N)				R(X•••O)			
(1,2BrG)4	2.43	2.46	2.43	2.46	3.64	3.09	3.64	3.09
(1,2IG)4	2.46	2.48	2.46	2.48	3.88	3.31	3.88	3.30
(1,2AtG)4	2.58	2.57	2.57	2.54	3.13	3.13	2.81	3.23
	∠(NX•••N)				∠(NX•••O)			
(1,2BrG)4	179	176	179	176	101	142	101	142
(1,2IG)4	179	177	179	177	100	133	100	134
(1,2AtG)4	175	175	176	175	151	159	148	169
	vdW(X•••N)				vdW(X•••O)			
(1,2BrG)4	0.71	0.72	0.71	0.72	1.08	0.92	1.08	0.92
(1,2IG)4	0.70	0.70	0.70	0.70	1.11	0.94	1.11	0.94
(1,2AtG)4	0.72	0.72	0.72	0.71	0.88	0.88	0.79	0.91

Table S2. Cartesian coordinates (in Å) of the undoped and halogenated reverse Hoogsteen base pairs, R-I and R-II ribbons and G-quartets, in xyz format. The title line provides the molecular system (rHS = reverse Hoogsteen, R1 and R2 refer to the R-I and R-II ribbons and G4 denotes a quartet)

```
32
rHS-H-basepair
N   2.79021100   2.97027200  -3.81266300
C   2.05276600   3.66018600  -4.78301100
H   1.03572200   4.01705500  -4.57969800
N   2.71486200   3.81237500  -5.91387200
C   3.94262600   3.19637600  -5.69441500
C   5.09387700   3.03878400  -6.54562800
O   5.27716600   3.44438000  -7.71853700
N   6.12001400   2.31625700  -5.89122300
H   7.02178600   2.14592600  -6.41319700
C   6.09286000   1.86809700  -4.58492400
N   7.22374200   1.23127700  -4.15677100
H   8.07658900   1.28598200  -4.71960700
H   7.28200800   0.98913600  -3.17006500
N   5.04139900   2.01436900  -3.77818600
C   4.01426500   2.66199200  -4.37829600
H   2.50398600   2.72798400  -2.86346800
N  11.65718000   4.67293500  -9.25393900
C  12.34181800   3.76740500  -8.43435200
H  13.43858700   3.74529800  -8.39897400
N  11.52983700   2.98582400  -7.74857300
C  10.25079500   3.38057400  -8.12629700
C   8.95128000   2.91668800  -7.71412200
O   8.65204600   2.03094900  -6.87740900
N   7.92076500   3.61291700  -8.38812200
H   6.92178700   3.37461700  -8.14697800
```

```
C   8.09067900   4.65990700  -9.27261000
N   6.93961000   5.20927400  -9.76649200
H   6.04903700   4.98659100  -9.31417400
H   7.02958000   6.07939300 -10.28787000
N   9.28463100   5.11420200  -9.65447500
C  10.30569500   4.44326400  -9.07010000
H  12.05470900   5.38103500  -9.87250900
32
rHS-Cl-basepair
N  -0.00195000   1.51182200  -4.04583200
C   0.13143300   2.54476400  -3.11081500
H  -0.49133300   2.58570500  -2.20850800
N   1.06647900   3.41369500  -3.44776800
C   1.57917400   2.94433300  -4.65177900
C   2.63030900   3.47591100  -5.50123000
O   3.34287900   4.46558800  -5.38075700
N   2.77133300   2.62866600  -6.67191200
H   3.44625300   2.98487000  -7.35517200
C   2.03745700   1.49713800  -6.97934400
N   2.31094500   0.87689300  -8.17904100
H   2.54922200   1.52331700  -8.94148200
H   1.54771300   0.25502600  -8.46191800
N   1.10111200   1.00818100  -6.16941500
C   0.92267500   1.75330800  -5.04818100
H  -0.62805200   0.70682200  -4.00641200
N  -2.17669900   1.89292900  -7.57318900
C  -1.66089800   0.99595500  -8.50644700
H  -2.13318700   0.02416800  -8.69208500
N  -0.57435300   1.45394200  -9.10742400
C  -0.35877100   2.70529300  -8.54702400
C   0.73036600   3.63279900  -8.76063900
O   1.71139300   3.53633700  -9.48760200
N   0.53642500   4.82177500  -7.91300700
C  -0.47083300   4.98932100  -6.96487600
N  -0.56411000   6.19046500  -6.26076100
H  -1.39976700   6.11858100  -5.66487200
N  -1.41406600   4.07780800  -6.76873700
C  -1.34206800   2.99615200  -7.57704200
H  -2.99443600   1.76950000  -6.97455200
Cl   1.71480100   6.08065800  -8.17719800
Cl   0.78699000   6.52039300  -5.10177600
32
rHS-Br-basepair
N   2.00781600   1.36855000  -2.62310100
C   1.04739500   2.35563300  -2.81617900
H   0.15013300   2.43893800  -2.19319100
N   1.35326200   3.14003400  -3.83685900
C   2.55149700   2.65947500  -4.34881600
C   3.36651300   3.11422600  -5.46316600
O   3.23458200   4.05234700  -6.23770600
N   4.52169500   2.23919900  -5.59257600
H   5.15252400   2.52237200  -6.34838500
C   4.83571100   1.15632500  -4.79367100
N   6.00984800   0.48775500  -5.05571400
H   6.40712200   0.51983800  -5.99464300
H   6.11647300  -0.39619000  -4.55621500
N   4.08069900   0.76798600  -3.77305400
C   2.97918400   1.53968500  -3.59737400
```

```
H   2.01174900   0.63865900  -1.90878400
N  -4.81764400   3.72537700  -9.54789300
C  -4.72756000   2.99460000 -10.73482200
H  -5.62345600   2.68642100 -11.28768400
N  -3.47802300   2.73655600 -11.08122200
C  -2.70570000   3.32029900 -10.08661000
C  -1.26087000   3.37391600  -9.94946100
O  -0.39179200   2.91294700 -10.66924500
N  -0.91165000   4.15192700  -8.73989000
C  -1.81436100   4.68801300  -7.82068500
N  -1.35323000   5.43755500  -6.75064600
H  -2.18439500   5.75937500  -6.23802100
N  -3.13413900   4.57579400  -7.98506400
C  -3.51959700   3.94401900  -9.11652800
H  -5.66146200   4.04475800  -9.07087400
Br  -0.23664100   4.49073400  -5.37188200
Br   0.97193700   4.45481000  -8.54669200
32
rHS-I-basepair
N   3.98958500   1.85296100  -1.41140300
C   2.83760000   1.10422100  -1.66968000
H   2.39201300   0.45676300  -0.90424700
N   2.38300600   1.27641000  -2.89874800
C   3.26660600   2.17402200  -3.48210300
C   3.25225500   2.76734300  -4.80804100
O   2.44709200   2.59965100  -5.71447900
N   4.36962300   3.70316200  -4.95432500
I   4.24300700   4.82836000  -6.78146600
C   5.35528000   3.95420400  -3.99799300
N   6.34570600   4.87608700  -4.26550000
I   7.86809600   4.40423900  -5.82076300
H   6.89355400   4.96624900  -3.39920500
N   5.32922400   3.37027900  -2.79206900
C   4.27938300   2.55005500  -2.57346600
H   4.53085200   1.89270300  -0.54714500
N  11.44558000   3.08354900  -8.70625000
C  10.83595100   3.33208600  -7.48484300
H  11.30388400   3.10036500  -6.52271100
N   9.63461700   3.86675400  -7.64882700
C   9.43417000   3.97711500  -9.01810200
C   8.30608300   4.48291700  -9.78096800
O   7.23548200   4.95210700  -9.42366000
N   8.60437400   4.36124600 -11.20016700
H   7.82971800   4.66748400 -11.79640600
C   9.76415600   3.86031300 -11.76266400
N   9.83435000   3.79320900 -13.13140400
H   9.22190800   4.36399400 -13.71226700
H  10.74624000   3.54707200 -13.51653800
N  10.78593300   3.41472200 -11.03898900
C  10.56813800   3.48587700  -9.70321500
H  12.36844800   2.67579900  -8.86291500
32
rHS-At-basepair
N   4.06003900   1.76936400  -1.34267500
C   2.99323600   0.89433000  -1.57018900
H   2.62752300   0.22130100  -0.78466800
N   2.51223200   0.98593000  -2.79785400
C   3.28801800   1.95845600  -3.41226800
```

C	3.19832500	2.52192600	-4.74974100
O	2.39905700	2.24633200	-5.63915500
N	4.20020600	3.55834100	-4.93133000
At	3.88182300	4.67564900	-6.88025600
C	5.16353500	3.93987900	-3.99989600
N	6.04713800	4.94648200	-4.31832500
At	7.69894500	4.50781400	-5.91443700
H	6.58984700	5.12596600	-3.46227700
N	5.21305500	3.38939700	-2.77514700
C	4.26267300	2.46538800	-2.52415500
H	4.59952800	1.89028500	-0.48497900
N	11.46295300	3.27070200	-8.64830200
C	10.78501600	3.50931200	-7.46233700
H	11.20830300	3.29853300	-6.47506300
N	9.57779500	4.00794700	-7.69258000
C	9.44615000	4.10265600	-9.07158900
C	8.34650200	4.57490900	-9.89410500
O	7.24955100	5.02083400	-9.59073600
N	8.72076200	4.45792300	-11.29454500
H	7.99649900	4.80373900	-11.93108900
C	9.91987500	3.98188400	-11.79319400
N	10.09527600	3.99254900	-13.15402000
H	9.29116000	4.03113700	-13.77910100
H	10.92624200	3.50895200	-13.49553300
N	10.90912200	3.55396600	-11.01540800
C	10.62727500	3.64039200	-9.69284800
H	12.40425000	2.88904500	-8.75276000

32
R1-H-dimer

N	3.76615100	1.50541900	-3.47211600
C	2.48443900	1.75777100	-3.97612700
H	1.61420300	1.83397800	-3.31211100
N	2.47256600	1.88624900	-5.28963800
C	3.79660700	1.71298200	-5.68110500
C	4.41246200	1.73657700	-6.99434900
O	3.90766100	1.91753900	-8.11075800
N	5.82191100	1.50272400	-6.88058500
H	6.32389400	1.49322000	-7.80009300
C	6.53068100	1.27737200	-5.72146700
N	7.87643700	1.07274000	-5.84002200
H	8.38317000	1.07171400	-6.73325700
H	8.38680700	0.89069900	-4.97888300
N	5.96512800	1.25247100	-4.50993900
C	4.62816700	1.47181200	-4.55629600
H	4.04155800	1.36158300	-2.50023400
N	6.69008700	1.67604800	-11.69063100
C	6.06426100	1.71880000	-10.44514100
H	4.99524900	1.90784500	-10.27858600
N	6.92816600	1.49987200	-9.46717300
C	8.15506800	1.30656000	-10.07153900
C	9.44085600	1.03362200	-9.48259200
O	9.76214900	0.90625800	-8.29626400
N	10.43489600	0.91417700	-10.51812300
H	11.38343500	0.75721800	-10.16465200
C	10.20790600	1.03266400	-11.88343600
N	11.30153700	0.93360700	-12.71412700
H	12.12134300	0.40911800	-12.40861800

H 11.07632200 0.87366800 -13.70831400
N 9.01318700 1.27647400 -12.40550100
C 8.03435800 1.41333400 -11.47428900
H 6.25201200 1.81484500 -12.60247900
48
R1-H-trimer
N 8.97992965102586 -1.40674503759260 -12.88613540271741
C 9.92542087749755 -1.68091159350519 -13.87357160431962
H 11.01052851997738 -1.72253445875164 -13.71101743524010
N 9.35228963453730 -1.85558408928782 -15.05296304399107
C 7.99258108041111 -1.68550200450966 -14.85174390869455
C 6.89591202541882 -1.71512238027551 -15.78303475813619
O 6.92250248224518 -1.91261711989019 -17.01775059909540
N 5.66698218202462 -1.46664045914674 -15.12146075881671
H 4.82609714445160 -1.46511528143306 -15.75447147008934
C 5.51147103841486 -1.20858573317374 -13.77087999196052
N 4.24142813340085 -1.00668858956083 -13.31892228845611
H 3.41663921751465 -0.92822326169988 -13.92998449244696
H 4.15004540721979 -0.74264965326799 -12.33995585212530
N 6.53314777800858 -1.16136132982729 -12.90599601522361
C 7.72838519089794 -1.40040540249810 -13.49092100762619
H 9.15968401470547 -1.21944002771172 -11.89910705320006
N 13.89754675477910 -1.42260862240406 -20.03441868447014
C 15.02837796960834 -1.30684973361750 -19.21749061618228
H 16.02846622018510 -1.15618694863684 -19.64321031803090
N 14.73679709322199 -1.39567475227793 -17.93316570953686
C 13.35793727364998 -1.57929102055794 -17.88749905974934
C 12.45237189250932 -1.72977406450677 -16.76241191449288
O 12.68526508065742 -1.72564311759988 -15.54674386554952
N 11.10851035712704 -1.89782460475894 -17.23058369530773
H 10.39364433736674 -1.97214584196225 -16.46214817933642
C 10.68626297219856 -1.89367995926530 -18.54030690720449
N 9.35020045974149 -2.07838772196133 -18.76971639495461
H 8.63118374872048 -2.02242134360418 -18.03080782405596
H 9.05426331970826 -1.94456337232173 -19.73487377650205
N 11.51642050786894 -1.74426317800111 -19.58020279140471
C 12.80790217981268 -1.59862885476478 -19.19525413683843
H 13.85267351346437 -1.38346277585346 -21.05306819731842
N 2.81379771112554 -1.83175689063406 -19.08317247708816
C 3.92846677206295 -1.82209092543375 -18.24747614456048
H 4.95418189783050 -2.04425173906278 -18.56673898524454
N 3.59623949502871 -1.50715167607575 -17.00574071944352
C 2.22929065876852 -1.30169727428349 -17.01349502338168
C 1.34248502508345 -0.93915738582951 -15.93822302148335
O 1.58482935384659 -0.72843444489069 -14.74431802462383
N -0.00493052274832 -0.83672608041115 -16.42987062219629
H -0.69413355755387 -0.61393640790663 -15.70567857429992
C -0.41307586820748 -1.05136004484420 -17.74099645375307
N -1.75771527501995 -0.95304288117735 -18.00482383777398
H -2.36358744010858 -0.40379910654957 -17.39593659908676
H -2.01123579648941 -0.98576361136005 -18.99281598847299
N 0.42168535643546 -1.38151946601976 -18.71866847644485
C 1.71013510949304 -1.50011402428196 -18.31135473083279
H 2.79559896208144 -2.05050426701336 -20.08057767824000
32
R1-Cl-dimer
N 3.59006200 1.63176600 -2.78691900
C 2.31457900 1.19265600 -3.14265200

```
H   1.51201700   1.10090400  -2.40020600
N   2.21199400   0.91246500  -4.43160100
C   3.46861300   1.17508600  -4.95485900
C   3.94119600   1.06030400  -6.32646600
O   3.35146200   0.71263300  -7.33269300
N   5.35793800   1.48745400  -6.38656300
C   6.12438700   1.89116300  -5.30869700
N   7.42157900   2.38207100  -5.51997800
H   7.78206100   2.64263400  -4.59188400
N   5.65860600   1.94835100  -4.06493800
C   4.34787700   1.62655100  -3.94607200
H   3.92302300   1.90707700  -1.86199600
N   7.44295700   2.38102800 -12.47881300
C   6.58496900   2.34462400 -11.38601900
H   5.60810500   2.84123400 -11.38475800
N   7.09407700   1.65062900 -10.37980500
C   8.33101300   1.20992500 -10.81326100
C   9.30812100   0.37785700 -10.13095400
O   9.28823300  -0.13203900  -9.03133400
N  10.46122500   0.15499900 -11.05331000
C  10.60284500   0.68950700 -12.32790100
N  11.70442700   0.32174000 -13.10851600
H  11.58577400   0.78301700 -14.02122500
N   9.68161600   1.46451200 -12.88281600
C   8.57173200   1.65652600 -12.13050700
H   7.28931300   2.84695300 -13.37465000
Cl   6.04107100   1.53466800  -8.02710900
Cl   8.63316200   1.16814700  -6.13314200
Cl  11.63542600  -0.98797800 -10.45110800
Cl  13.30708000   0.95487000 -12.51099200
48
R1-Cl-trimer
  N   9.01110092436307     -1.19493661487754    -11.97482213887815
  C   9.99656520922630     -1.06834168525486    -12.94535417335850
  H   11.06443133890517     -1.02012773108670    -12.70325122829761
  N   9.48406763635741     -1.02377714847434    -14.16548794573358
  C   8.11207471559062     -1.12945796406664    -14.00685042093958
  C   7.05533690655681     -1.17056980027687    -15.00569924334331
  O   7.12135326917358     -1.12309211725527    -16.22041940888369
  N   5.75503443716519     -1.33155999900442    -14.32193116815470
  C   5.56667347637957     -1.40126014783827    -12.95436685333357
  N   4.29423688272539     -1.68175433686264    -12.44091421144728
  H   4.39251066255330     -1.72062783519301    -11.41726984645484
  N   6.56401066522728     -1.33047500307985    -12.07626379070510
  C   7.79255481742084     -1.23876025325447    -12.63656591049563
  H   9.14040652385846     -1.25486419988840    -10.96354317913067
  N   14.68440799922416     -1.83547791096885    -20.61946628030138
  C   15.53474580499277     -2.78391686791876    -20.04982037524343
  H   16.42111627547004     -3.15181050771784    -20.58104176734626
  N   15.14577171124938     -3.15850328688709    -18.84225211930217
  C   13.99021889388836     -2.43112190274324    -18.59791677849258
  C   13.13245969735017     -2.42366571149070    -17.42229569583818
  O   13.22541691443929     -3.04977435018776    -16.38181005252947
  N   12.03610408515541     -1.45194430799501    -17.62737139294020
  C   11.82982153097397     -0.70262895456850    -18.76928890264493
  N   10.82546875417948      0.27576616969416    -18.79524097722526
  H   10.87225492110752      0.70978766987928    -19.72688193706302
  N   12.61876868961128     -0.76892149848291    -19.83884825267402
```

C 13.68164789234072 -1.59622040216674 -19.69404641951826
H 14.76109087682417 -1.39671155821218 -21.53781265993658
N 1.78709542509343 -2.50818548167166 -19.17916290416984
C 2.86782471088322 -2.45165507415253 -18.30887976853777
H 3.86693358482752 -2.81424500418134 -18.57587622521914
N 2.53687815565023 -1.90845148206971 -17.14673653711295
C 1.19527605203269 -1.58975942777160 -17.24490215103755
C 0.31479577367974 -0.98057413550332 -16.26269552370981
O 0.53002750176915 -0.59895094501257 -15.13230159400879
N -1.05516128625362 -0.89551154810647 -16.85027826852870
C -1.41470859579656 -1.26564216887960 -18.14069230048428
N -2.75811121123868 -1.19886459926073 -18.52556408449283
H -2.80876664238465 -1.56703061535055 -19.48579099663463
N -0.54827353575250 -1.77787477446411 -19.00354586623153
C 0.70170280026770 -1.95938465494671 -18.51432331955286
H 1.77781898350899 -2.87177937512130 -20.13357614750128
Cl -3.42884982474237 0.48850489449626 -18.67327999394389
Cl -2.27797789214080 -0.37330004289552 -15.71913401216364
Cl 4.37935436093543 -1.56464757955223 -15.43151254183945
Cl 3.01657596526601 -0.42576524100280 -12.72134268344990
Cl 9.11537086186094 -0.32061961674374 -18.66395876990372
Cl 10.97588414022464 -1.23181309163002 -16.20553360126531
32
R1-Br-dimer
N 3.33464700 1.42063600 -2.56125700
C 2.09475900 0.93960900 -2.98125100
H 1.26094900 0.81352200 -2.27939700
N 2.06417900 0.66579400 -4.27622100
C 3.33354300 0.97693200 -4.73620900
C 3.88008000 0.89299300 -6.08464200
O 3.33632700 0.52808800 -7.11411900
N 5.27000000 1.37004900 -6.08656400
C 5.96906700 1.78774400 -4.97009900
N 7.25606300 2.31980600 -5.11955700
H 7.54374200 2.61107900 -4.17432600
N 5.44776900 1.81615700 -3.74224200
C 4.14693300 1.45080600 -3.68350900
H 3.61290300 1.69961900 -1.61957900
N 7.35716700 2.37280200 -12.33035500
C 6.53785000 2.32634600 -11.21252500
H 5.53800900 2.77302800 -11.18596700
N 7.11624200 1.68263600 -10.20713000
C 8.35832300 1.28752900 -10.66867900
C 9.41795700 0.56004300 -9.98759700
O 9.45933000 0.11867100 -8.85745400
N 10.57714900 0.43351500 -10.90489500
C 10.61597200 0.86302200 -12.22658000
N 11.79944000 0.72697100 -12.95385900
H 11.63520500 1.19083100 -13.85894200
N 9.59413500 1.48524000 -12.80724800
C 8.53206700 1.71216600 -12.00243000
H 7.14649500 2.79788800 -13.23495300
Br 12.13312900 -0.29172100 -10.06402000
Br 12.22994600 -1.13611100 -13.51361400
Br 8.68222100 1.00476200 -5.59569700
Br 6.07147000 1.47840000 -7.87175900
48
R1-Br-trimer

N	8.98237959638552	-0.73561332019891	-11.85424361237220
C	9.98135386704931	-1.12296731821383	-12.73347616210148
H	11.03908260748989	-1.17576942313072	-12.45333538024502
N	9.49010398829908	-1.41024268188015	-13.93185108316349
C	8.12196834097945	-1.20626523869234	-13.85161196530319
C	7.08204895772002	-1.36325328156834	-14.85948446523113
O	7.18477926076204	-1.73915344966753	-16.01502293152414
N	5.79039305542819	-0.95256625802473	-14.30092424022383
C	5.57957739669547	-0.56942524435039	-12.99233623197809
N	4.31643330433658	-0.11370766798917	-12.59846635140286
H	4.40932588187715	0.16220255341720	-11.61105138920689
N	6.54922492021119	-0.49944814346138	-12.07561799041506
C	7.78191559649120	-0.77771744103267	-12.55266559320327
H	9.09111567607981	-0.46401910505550	-10.87603221283424
N	14.61655897221527	-2.21547242583068	-20.66226945343976
C	15.44610760085449	-3.21771849865568	-20.15971731565737
H	16.31229294287404	-3.58244936115558	-20.72554981323658
N	15.06543851268042	-3.64622373354787	-18.96639462440726
C	13.93682840434781	-2.90041413926188	-18.66277179780106
C	13.09289785607950	-2.92932867176336	-17.47396211055272
O	13.19783390225844	-3.62441516549770	-16.47600050397824
N	12.02924357679420	-1.92575512752144	-17.59328637995728
C	11.82885527197099	-1.11248495449678	-18.68896328821031
N	10.84898187717299	-0.11070656107860	-18.64329332222451
H	10.92501873056066	0.38332331035362	-19.54388856064431
N	12.59510640919576	-1.13825658976677	-19.78310394173970
C	13.63567985074391	-1.99873452945227	-19.70669753043836
H	14.68914583212731	-1.73346453409311	-21.55896048597506
N	1.59409405576955	-0.24589382229917	-19.36219816602715
C	2.73434489293591	-0.17754434202558	-18.57817996358262
H	3.66684260018290	0.28823688883974	-18.91499518359854
N	2.54613397563215	-0.74559353672851	-17.39362478397909
C	1.24276337884762	-1.20819316894231	-17.38872992847827
C	0.50023715710703	-1.89775105031218	-16.34584035932331
O	0.85130237236736	-2.23663886758282	-15.23381376583071
N	-0.88459605332317	-2.13217560314494	-16.81987566309895
C	-1.37224435252525	-1.82089792523589	-18.08442533984679
N	-2.71783752422564	-2.05172493694170	-18.37249677461676
H	-2.88245302199227	-1.66602185844504	-19.31347821916468
N	-0.62846462123130	-1.22396323634627	-19.01117387770715
C	0.62435894040508	-0.90289272133807	-18.61860128116614
H	1.47774515213770	0.10539622664212	-20.31434176095484
Br	-3.21003341539400	-3.96396413630528	-18.60897215952515
Br	-2.04469846656008	-2.81365263745920	-15.46236008639991
Br	2.86948308770986	-1.46527810477919	-12.54435789936975
Br	4.34542501966186	-0.87811433621993	-15.63750228798535
Br	10.90956105718805	-1.74466311934765	-15.97514778356182
Br	8.95699763562566	-0.74489042041122	-18.67139494831530

32

R1-At-dimer

N	3.25210500	1.40660000	-2.40048400
C	2.03188000	0.88189800	-2.82137900
H	1.19876800	0.73493900	-2.12259100
N	2.01503300	0.59463700	-4.11576300
C	3.27331900	0.94236300	-4.57430500
C	3.84255800	0.86870900	-5.91718900
O	3.30080000	0.46905800	-6.94432800
N	5.19145900	1.38275200	-5.93557700

```
C   5.87351600   1.82775200  -4.82344200
N   7.14220100   2.37242500  -4.99148300
H   7.41416100   2.71155700  -4.05504900
N   5.35376400   1.85455400  -3.58138200
C   4.06995300   1.45256600  -3.52096800
H   3.51752000   1.70048600  -1.45977300
N   7.43326900   2.43368900 -12.55035700
C   6.57450600   2.40014400 -11.46787800
H   5.58023600   2.85868000 -11.47059900
N   7.11432400   1.74731400 -10.44020600
C   8.36807700   1.33455300 -10.85756400
C   9.40620900   0.60495700 -10.14596700
O   9.37858200   0.17702400  -9.00166200
N  10.58177600   0.46833600 -10.98833500
C  10.67684700   0.88553900 -12.30874900
N  11.87676200   0.70490300 -12.97542500
H  11.77232500   1.18816300 -13.88101000
N   9.67905800   1.52147900 -12.94091400
C   8.59181500   1.75874300 -12.18340400
H   7.25781500   2.86336700 -13.46042400
At  12.34261000  -0.34367400  -9.82901700
At  12.29128500  -1.46732100 -13.72140300
At   8.88542200   0.83051700  -5.35085200
At   6.06511600   1.51214500  -8.07519500
48
R1-I-trimer
  N   8.98128939600825     -1.37819582076938    -11.64197166720651
  C   9.99431068896057     -1.38196853561180    -12.58189791399040
  H   11.05902696809227     -1.38457094318649    -12.32580990702053
  N   9.50246949571611     -1.38420488707479    -13.81813772203626
  C   8.12116090737992     -1.38220322427359    -13.69634870156753
  C   7.07404530132383     -1.41438329044294    -14.70933965176772
  O   7.21026568217897     -1.45180921458214    -15.92570502042169
  N   5.76554547041489     -1.44472175168714    -14.08730900628585
  C   5.55294614852719     -1.39361323076718    -12.72730850944786
  N   4.24660590455298     -1.50194403536737    -12.24481285075548
  H   4.33187551387065     -1.53578550118633    -11.21794413346928
  N   6.53110289730054     -1.33996607567741    -11.80859614325548
  C   7.77261382278255     -1.37810056330058    -12.33155272885290
  H   9.08626494567055     -1.36920570879778    -10.62601677938365
  N   14.81755011779957     -1.43514874367663    -20.77754782483242
  C   15.60363245498065     -2.51488049875216    -20.37827098017447
  H   16.47803682115306     -2.83347464352784    -20.95926628877894
  N   15.17468481134044     -3.07442407291856    -19.25618281164340
  C   14.05815575026660     -2.33860211208624    -18.89446647047511
  C   13.17168308873714     -2.47780464850602    -17.74262865212643
  O   13.23780083214900     -3.30183269346564    -16.83736427521078
  N   12.14756378581223     -1.44901645318714    -17.75185667764550
  C   11.99775370898544     -0.51337443910372    -18.75064759393501
  N   11.02313411908342      0.47793516583754    -18.61144487923127
  H   11.15402261373039      1.10083261694862    -19.42309371733239
  N   12.79557249513632     -0.42049290899167    -19.82818491436637
  C   13.81148129685961     -1.30748927117732    -19.83006719998408
  H   14.92854953646071     -0.85265303616491    -21.60837258493497
  N   1.40382571494039     -3.08112302157668    -19.12000608153331
  C   2.53006090512069     -3.03737023998381    -18.32079816087364
  H   3.45264553641020     -3.58479570205283    -18.54133865212184
  N   2.34364619371628     -2.24303816323041    -17.26925206805452
```

```
  C   1.05718287073546     -1.74293301954585    -17.37698273434176
  C   0.32041598163435     -0.84271300583194    -16.50551529194582
  O   0.68709228027333     -0.31974464858244    -15.46676130379762
  N   -1.03236574782786     -0.64799285180344    -17.02968206028033
  C   -1.51535273583621     -1.17551188533963    -18.22330482140557
  N   -2.83354894476986     -0.92669503590406    -18.57928189465209
  H   -3.01887657792831     -1.50455647759890    -19.41306357141546
  N   -0.78408797926248     -1.97522819336539    -19.00587391004019
  C   0.44698723253613     -2.25974796984503    -18.53694294987050
  H   1.29147470781076     -3.59784381954158    -19.99432659316528
  I   -3.25137405442099      1.10344064723258    -19.36972858007104
  I   -2.28830814300850      0.42290386296485    -15.66715319330947
  I   2.94641278835821      0.25385256346698    -12.51735783538035
  I   4.13463431477152     -1.78383410562632    -15.54987939297532
  I   10.89802956084752     -1.43662723945405    -15.91598046664313
  I   8.91374564062547     -0.15517224288546    -18.91143395199051
32
R1-At-dimer
N   3.25210500   1.40660000  -2.40048400
C   2.03188000   0.88189800  -2.82137900
H   1.19876800   0.73493900  -2.12259100
N   2.01503300   0.59463700  -4.11576300
C   3.27331900   0.94236300  -4.57430500
C   3.84255800   0.86870900  -5.91718900
O   3.30080000   0.46905800  -6.94432800
N   5.19145900   1.38275200  -5.93557700
C   5.87351600   1.82775200  -4.82344200
N   7.14220100   2.37242500  -4.99148300
H   7.41416100   2.71155700  -4.05504900
N   5.35376400   1.85455400  -3.58138200
C   4.06995300   1.45256600  -3.52096800
H   3.51752000   1.70048600  -1.45977300
N   7.43326900   2.43368900 -12.55035700
C   6.57450600   2.40014400 -11.46787800
H   5.58023600   2.85868000 -11.47059900
N   7.11432400   1.74731400 -10.44020600
C   8.36807700   1.33455300 -10.85756400
C   9.40620900   0.60495700 -10.14596700
O   9.37858200   0.17702400  -9.00166200
N  10.58177600   0.46833600 -10.98833500
C  10.67684700   0.88553900 -12.30874900
N  11.87676200   0.70490300 -12.97542500
H  11.77232500   1.18816300 -13.88101000
N   9.67905800   1.52147900 -12.94091400
C   8.59181500   1.75874300 -12.18340400
H   7.25781500   2.86336700 -13.46042400
At  12.34261000  -0.34367400  -9.82901700
At  12.29128500  -1.46732100 -13.72140300
At   8.88542200   0.83051700  -5.35085200
At   6.06511600   1.51214500  -8.07519500
48
R1-at-trimer
  N   9.02476540101266     -1.11624555344569    -10.81795310429990
  C   10.03952354840287     -1.22021174487986    -11.75191762779536
  H   11.10388278018933     -1.17117462303312    -11.50157148876364
  N   9.54637637465330     -1.38639717032959    -12.97717142759182
  C   8.16016593945885     -1.39774521383502    -12.85347495489046
  C   7.07072650390868     -1.49513470930207    -13.77653126452481
```

O	7.25207959069095	-1.60722322456632	-15.06381635978255
N	5.83168195432337	-1.44515487474496	-13.26070376264757
C	5.60683683670828	-1.29092606688402	-11.92380745583049
N	4.30031854520429	-1.30153934317076	-11.51744898636700
H	4.11953440179847	-0.99100755646832	-10.55997101491102
N	6.57307032925945	-1.14949709233424	-10.97047709116533
C	7.80442161981392	-1.22229259029722	-11.48741974753343
H	9.13557117271330	-0.97322204231476	-9.81327480803372
N	13.94568258149883	-2.07672510962307	-21.05144307205037
C	14.99923676692969	-2.23807496281620	-20.15198273306673
H	16.02459959545987	-2.41946966311421	-20.49785728828546
N	14.61805576777331	-2.15216618106211	-18.88418460838568
C	13.25537741133849	-1.92110076088954	-18.93785150783302
C	12.23418182532392	-1.75349285852005	-17.92657832729796
O	12.55334908922758	-1.81876679168121	-16.66784210358217
N	10.96998201923535	-1.53768262998592	-18.34660557077640
C	10.67776958498328	-1.49089352986483	-19.67280959382963
N	9.39077699803548	-1.20463570295395	-20.03414746812064
H	9.19128665914841	-1.33822117295874	-21.03019436545957
N	11.54995998341314	-1.67617444130958	-20.71897037027408
C	12.79951232262276	-1.86815123033425	-20.29219538464738
H	13.98479970018239	-2.10104534073627	-22.07094005718482
N	2.54523660270546	-1.35291491643199	-20.02269071380578
C	3.68879086683228	-1.44553923920614	-19.26214281754850
H	4.70103476019198	-1.53898045674295	-19.67615730305049
N	3.40116031484441	-1.39584184241949	-17.96041152972897
C	2.01766434082179	-1.26837951376977	-17.85654109587605
C	1.13470046141784	-1.12600109362696	-16.71425946460995
O	1.45001846810834	-1.07474269410386	-15.52128837762713
N	-0.22660212525038	-1.00362153223815	-17.12919796524412
C	-0.68803844252460	-1.05102650172635	-18.44819569289533
N	-2.02541464301726	-0.91887724404894	-18.66633098901691
H	-2.16062697531530	-0.89748743305603	-19.68814300408919
N	0.16644751998443	-1.17106604031344	-19.48949195052861
C	1.46029838346445	-1.24254358786637	-19.15584807255217
H	2.48737665382838	-1.37702531104298	-21.04274107886281
At	-3.35481782274395	-2.93288719959880	-18.19933393907930
At	-1.61709987810006	-0.45522960762199	-15.41860989406567
At	2.71226435682807	-1.12763407613420	-13.05615884438627
At	5.25368259677959	-1.51993772806872	-16.25414814158055
At	10.94190645957145	-1.59525660824693	-15.02480830510218
At	7.67987691826292	-1.35646426228086	-18.56116839541863

32
R2-H-dimer1

N	-4.27327511459580	-0.20310656842641	-0.64638240736868
C	-5.44115472637549	0.51122308500299	-0.94647157856487
H	-6.38316087738696	0.31281262673179	-0.42099711355402
N	-5.25426018729492	1.40645385252328	-1.89636552644994
C	-3.91628225999500	1.28535854886901	-2.24988181530080
C	-3.13996919254588	1.99266417094500	-3.25205720342201
O	-3.44374927977649	2.88044718095267	-4.03718487941998
N	-1.77647263398317	1.47403969627284	-3.25808257316964
C	-1.24712145325680	0.48323523242675	-2.45244045852748
N	0.04941331456448	0.11417615483132	-2.64023441383979
H	0.67634692813937	0.73962394722016	-3.14417812460693
N	-1.98556754760298	-0.13329603094805	-1.52072031908262
C	-3.28300693469523	0.28161563890553	-1.48116772265823

```
  H   -4.16987463365043      -0.97487082730928       0.01380444558509
  H   -1.19526916696378       1.89868886652349      -3.98652891207232
  H   0.47096294863966      -0.60138725483497      -1.99980657255223
  N   3.18931945298382      -2.52220536714282      -2.04845722357044
  C   4.41543207858253      -3.09047064554965      -1.67667841002273
  H   5.17051401622395      -3.36013004274909      -2.42518777129876
  N   4.52379798068601      -3.24544989950167      -0.37167568589134
  C   3.32785105842853      -2.76876040820970       0.15025998101675
  C   2.86279988910034      -2.69706875304182       1.52345934330003
  O   3.39126399505541      -3.02283612851773       2.57766930457797
  N   1.51590967110239      -2.13781062776840       1.54598073199529
  C   0.76091222257558      -1.71216706883435       0.46910094944831
  N   -0.49865358452603      -1.25090057890536       0.69768903079120
  H   -0.76193064009944      -0.96871493438320       1.64051533658855
  N   1.22998517949285      -1.76992199753492      -0.78412149643129
  C   2.47641817190320      -2.31204331639547      -0.88248785416633
  H   2.85311405478210      -2.33382234172548      -2.99360502312210
  H   -1.05942341752716      -0.88357499588916      -0.10856264546880
  H   1.11328343801538      -2.11267981353732       2.48714969725813
32
R2-H-dimer2
  N   3.72750383517111      -2.01397403804558      -1.05290297930820
  C   5.02013146519331      -2.54867190890244      -1.11291598010274
  H   5.69553820492084      -2.31715501393742      -1.94603130014381
  N   5.29872719758769      -3.32636314017796      -0.08431846704735
  C   4.14601372321800      -3.31496809312091       0.69408114900617
  C   3.83420759512411      -3.96263787339223       1.94222282636710
  O   4.55309354400280      -4.70672737318286       2.65106731274216
  N   2.51485851600869      -3.66638119522550       2.35833371517004
  C   1.62525470208147      -2.83467536603131       1.70846961708077
  N   0.40871807014937      -2.68400107853090       2.31169109493838
  H   0.25966168492183      -3.04458677997011       3.25645157865203
  N   1.89835506458518      -2.21733769663160       0.55834411751646
  C   3.14401205065471      -2.49479658908975       0.10537628568951
  H   3.27634234895405      -1.39070752775177      -1.72367394249679
  H   -0.23427977769136      -2.00028401534378       1.91823208350185
  H   2.17657185211354      -4.07859390173597       3.26927974766753
  N   2.77536443086055      -5.83544414857424       9.00985121793376
  C   1.43808435206538      -5.42105872316501       9.01845862114343
  H   0.83912983519018      -5.44395030003874       9.93740020615626
  N   1.01989076147220      -5.02211169561553       7.83249071593584
  C   2.11995308108739      -5.17763682109722       6.99559452700959
  C   2.29363472080278      -4.90644661612824       5.59157984167057
  O   1.47002201590621      -4.44629506095872       4.76505361180583
  N   3.60250476376467      -5.23114438303551       5.16414610559192
  C   4.62270485914351      -5.70944554301577       5.96199376140829
  N   5.81059161348035      -5.93831075737220       5.32675960408908
  H   5.94907938920930      -5.60367937243375       4.37069080764865
  N   4.48492512158769      -5.95886110727639       7.26469695843663
  C   3.23693637347472      -5.68606476156467       7.71457421591580
  H   3.32784484006569      -6.18236315227719       9.79473111370854
  H   3.85155012522349      -5.06273851312498       4.15195466028410
  H   6.61242506967052      -6.19186577325169       5.89950455202862
48
R2-H-trimer
  N   -2.98495765379756       0.30275086582331       1.11710219998094
  C   -4.29145340839509       0.79296712351740       0.99864066429239
  H   -4.89048854575628       1.04193349411095       1.88300701239595
```

N	-4.67646268607799	0.90594208845717	-0.25793306443773
C	-3.58903291870225	0.47685444595363	-1.00877233977196
C	-3.41785442352105	0.37972098982080	-2.44663445073701
O	-4.17068664571765	0.64494281480280	-3.37445326968399
N	-2.07965842308239	-0.12142720781838	-2.73829094719139
C	-1.09423123191470	-0.47235527336549	-1.83383824379297
N	0.11247338611432	-0.88422577052245	-2.31535619772123
H	0.16931922335050	-1.22064703856852	-3.27547112840575
N	-1.29126651163286	-0.38681389986552	-0.51340706472068
C	-2.51785079424284	0.09547368809126	-0.16754389691319
H	-2.45871717334167	0.14241382489348	1.97706658735964
H	-1.88317756959506	-0.16332217044444	-3.74239120778841
H	0.84519614404339	-1.20472491589710	-1.63845379438454
N	4.17279857106912	-1.75944675943173	-1.75192152995316
C	5.46052588851598	-2.28029038593620	-1.55510226431464
H	6.25464053953234	-2.13123161062574	-2.29723555752605
N	5.57383177568077	-2.93411855776989	-0.41649779374100
C	4.31805841009369	-2.84735273728359	0.17512651950014
C	3.82133724884963	-3.35157338659429	1.43110570158478
O	4.42028210116889	-4.01627280901966	2.30704337543611
N	2.46690445717610	-2.99782094297425	1.62791881991722
C	1.66723905087215	-2.27384589933940	0.76615194370794
N	0.39506876124098	-2.04658874805317	1.16230763331148
H	0.09459300388321	-2.41239282635854	2.06800224150902
N	2.11755212800012	-1.81482252255906	-0.41742570967676
C	3.42170906083408	-2.11340500785404	-0.64579313889321
H	3.84605008428710	-1.19709609301645	-2.53841954079138
H	-0.22841227365154	-1.43199877756317	0.59056834147310
H	2.00632461636758	-3.29389566507104	2.53227932359977
N	1.41106971766885	-5.54070991003404	8.11836705769652
C	0.13123379718151	-5.00724921612588	7.92328281616326
H	-0.63814480733993	-5.07046708879283	8.70305315871998
N	-0.01376572786764	-4.45060117636841	6.73596596610461
C	1.21733083774074	-4.61845966692832	6.11064065461551
C	1.68356471865933	-4.22425354208590	4.80660926781045
O	1.07280440629549	-3.61404217857450	3.89682494146967
N	3.02788795760612	-4.61452910536382	4.60166176915844
C	3.82935597462249	-5.27775321322319	5.50985638419261
N	5.09749234963711	-5.55762279459911	5.08723219477335
H	5.43620883544769	-5.19414222654936	4.19506730116243
N	3.41803667494481	-5.64642338130350	6.72338327378506
C	2.13028951004698	-5.30074201999741	6.96156323614849
H	1.77022478465378	-6.01731866338937	8.94647997958744
H	3.47601371051160	-4.36650688590235	3.68007189593012
H	5.74594510853994	-5.96351555030061	5.75787424905865

32

R2-Cl-dimer1

N	-4.24592457002011	0.51966590222210	-0.60125096602827
C	-5.44485021430843	1.22086432238014	-0.76452989634076
H	-6.20604248118425	1.24603574525531	0.02510740317386
N	-5.52940962494581	1.81630070299386	-1.94023829167702
C	-4.34121304765640	1.50176845566805	-2.58537735819071
C	-3.87372917476245	1.90015941335068	-3.89691826243131
O	-4.37495239242293	2.58538577486459	-4.76421452007342
N	-2.51086013791458	1.30971203027109	-4.10836548278094
C	-1.79943716030535	0.50021235991847	-3.21965639831831
N	-0.58770406247211	-0.00392321976499	-3.66215937860333
H	-0.10876928382333	0.53435358207049	-4.39106171106591

```
  N   -2.29541613041399      0.18249291988016     -2.03429659155637
  C   -3.52430896036957      0.69184248929255     -1.76617462701767
  H   -3.92688254049949     -0.00494823142267      0.21596965951448
  Cl  -1.79317675089925      1.72359751318444     -5.66281968958680
  Cl  0.50740726160000     -0.64437606255845     -2.43979138765402
  N   3.66838912845385     -1.97186622616223     -1.48738092827388
  C   4.88140452644485     -2.64687253888872     -1.31756625865103
  H   5.66235189784514     -2.62431853764802     -2.08774194143827
  N   4.95288649993352     -3.28037572147654     -0.16107368541664
  C   3.74119500065059     -3.01924627215046      0.46391406909321
  C   3.25314542690196     -3.47385744203256      1.74945008806468
  O   3.75095612936163     -4.17628466657043      2.60489534824885
  N   1.87190099409548     -2.92325163833908      1.94811516118591
  C   1.16180564890923     -2.10071639406050      1.07041847707377
  N   -0.06905468578194     -1.63600616450938      1.50361930151417
  H   -0.55459116405863     -2.21216913866852      2.19857328549556
  N   1.67535962976047     -1.73402388799370     -0.09315828257064
  C   2.92326493466745     -2.20265906221062     -0.34758347654992
  H   3.35705050329689     -1.42541470354682     -2.29309529111462
  Cl  -1.14842950327343     -0.96991639238360      0.28053611804286
  Cl  1.13086645319098     -3.40267399096464      3.47243887393249
32
R2-Cl-dimer2
  N   3.52458184033346     -1.85661199650074     -2.07839314128143
  C   4.81911304915472     -2.38508418971954     -2.07083858824166
  H   5.47786294196953     -2.29812331510108     -2.94371577345675
  N   5.12381598549490     -2.97063867468277     -0.92699162818625
  C   3.98855430894392     -2.82543393745401     -0.14058972187625
  C   3.73754011063342     -3.28388870217982      1.20870233556128
  O   4.46148285018717     -3.90532684532443      1.97571210785059
  N   2.36899342642417     -2.89260452801274      1.60472966214791
  C   1.45934072594516     -2.18169845382569      0.81885021783531
  N   0.26325941301776     -1.83823742367480      1.43153418825432
  H   -0.02611966507172     -2.41324598684838      2.23067401658807
  N   1.73368100879244     -1.79624747073820     -0.41369542280676
  C   2.97493958741446     -2.13146675711387     -0.84038290315528
  H   3.05370424624660     -1.36067506940868     -2.83659154647537
  Cl  -1.09121404761197     -1.40004868512916      0.42149936453051
  Cl  1.91116188217742     -3.41282203956223      3.22946103123554
  N   2.77600409780919     -6.54420278801988      9.80751118974480
  C   1.48134104850664     -6.01602441047763      9.80009629931481
  H   0.82258375443921     -6.10346394922943     10.67292038102760
  N   1.17656988725941     -5.43009497940808      8.65646893866556
  C   2.31187522873032     -5.57480815626241      7.87002864221853
  C   2.56288246644857     -5.11576977711708      6.52094707082883
  O   1.83895304672462     -4.49407800461668      5.75412650769046
  N   3.93143071265882     -5.50687606004515      6.12473900827880
  C   4.84126839592516     -6.21776400737334      6.91043095564137
  N   6.03746568118994     -6.56050259446959      6.29785848120849
  H   6.32652927203618     -5.98612795179326      5.49818565045297
  N   4.56699024211225     -6.60360165970443      8.14289958665305
  C   3.32563258607873     -6.26880156671642      8.56961631023483
  H   3.24695468496129     -7.04034346896620     10.56553013535982
  Cl  4.38883167084122     -4.98678143698400      4.49985717509861
  Cl  7.39179406022699     -6.99956256354029      7.30731529905766
48
R2-Cl-trimer
  N   -4.04076797374599      0.24679452654891     -0.29965371682740
```

C	-5.29379456695060	0.86718403659792	-0.32545179599154
H	-5.97349544921716	0.82026459047281	0.53422831416200
N	-5.53285667094165	1.48307836901589	-1.46896698616777
C	-4.39551763693773	1.26651461854922	-2.23485158386766
C	-4.08704096388725	1.72646128742787	-3.57318857344084
O	-4.71706860555195	2.39574370031757	-4.36637476340907
N	-2.71734861227375	1.23661733112781	-3.93397669168326
C	-1.86924438578396	0.45752274130482	-3.14281917901637
N	-0.67784172150102	0.04686877438448	-3.71585547781643
H	-0.30833059515393	0.63198030792186	-4.47196919864456
N	-2.22222098042422	0.08012161783134	-1.92389905550187
C	-3.45011848251145	0.49598241703486	-1.52294569820795
H	-3.61033366441152	-0.27553583610269	0.46632657554182
Cl	-2.18558178421876	1.73520884903164	-5.53834016478397
Cl	0.57070247732614	-0.55068705111397	-2.62369155887884
N	3.85505989900713	-1.80617430821913	-2.13535464602551
C	5.10274058228909	-2.43837334962496	-2.11553175858385
H	5.77318456922038	-2.40820921208603	-2.98323304255414
N	5.34908479359625	-3.04270592363134	-0.96752195077637
C	4.22173083659588	-2.80611639168064	-0.19134148392577
C	3.91591111913609	-3.24731729419988	1.15229745326617
O	4.57597467455895	-3.92410569485585	1.92852938048612
N	2.57255157065995	-2.75410034783431	1.53001044230700
C	1.72571831547468	-1.97370642829052	0.74068684303307
N	0.54824490453722	-1.55371882910607	1.33088135180876
H	0.20624179409154	-2.10564359937243	2.12496260242460
N	2.05903844406609	-1.60422212786310	-0.48893195356870
C	3.27591179617206	-2.03529841261105	-0.90262494146979
H	3.41834257795137	-1.29087179493408	-2.90233390014704
Cl	-0.72464115152459	-0.96570616086835	0.27258511573218
Cl	2.05245220811649	-3.25083498968444	3.14184976711779
N	2.45212332000045	-6.42139381758707	9.71799251054816
C	1.20502423414165	-5.79093033342022	9.66746746771070
H	0.51420230614457	-5.81997381281347	10.51921953342833
N	0.98483383774229	-5.18871097030817	8.51296022431881
C	2.12934693094344	-5.42840048384501	7.76400897829377
C	2.45903125436003	-4.99832991358937	6.42267733958961
O	1.80959344833524	-4.32483869931743	5.63248148515664
N	3.80366939218253	-5.49827851622578	6.07120682575949
C	4.62932116209525	-6.27470701457532	6.88843039752100
N	5.81415858978783	-6.71245283706758	6.31617297484667
H	6.17268239888271	-6.16611999590290	5.52514330744459
N	4.28517786122653	-6.63150343403811	8.11181452502310
C	3.06137410000111	-6.19740962288540	8.49781196836527
H	2.85717314528119	-6.94935809096709	10.49249787926627
Cl	4.35477598324958	-5.02270331581642	4.46224961580151
Cl	7.09639667786185	-7.25251801712882	7.36919972233524

32

R2-Br-dimer1

N	-4.29954255035416	0.62515528966259	-0.61816376593167
C	-5.55001495841015	1.21783988025453	-0.83396720715803
H	-6.32714093313839	1.21025977694729	-0.05977883525445
N	-5.65541664962247	1.75624936482297	-2.03374415266074
C	-4.43048670672588	1.51466346786502	-2.64132467213630
C	-3.97355412000474	1.87759322647958	-3.96545218705414
O	-4.53004607611872	2.47326201507072	-4.86908225009620
N	-2.57824376324774	1.39226434544251	-4.14525912615517
C	-1.80283603338392	0.69326162833139	-3.21051637021841

```
  N   -0.53920342441010      0.33808837497833     -3.58584329390653
  H   -0.19022126762588      0.59035479779549     -4.51224057856504
  N   -2.29516410397538      0.39354763441815     -2.00597758472289
  C   -3.56761140531442      0.80953723656592     -1.77517440179525
  H   -3.97211327469360      0.14740927164514      0.22428143638793
  Br  -1.85893929032901      1.81870830247353     -5.88116230794462
  Br  0.61381853623612     -0.60694023680936     -2.40964034950042
  N   3.68988856228927     -2.16141760490596     -1.50910445245233
  C   4.94595691399647     -2.74018733009971     -1.28812434388814
  H   5.72587046845810     -2.72459986409949     -2.05937107094109
  N   5.05267378067222     -3.27662996121882     -0.08757447230049
  C   3.82285449288866     -3.04807809193885      0.51518067738493
  C   3.36517064661297     -3.41445322954310      1.83809320155044
  O   3.92475892969563     -4.00327997987436      2.74428449885887
  N   1.96418934250177     -2.94363559592184      2.01255676524477
  C   1.18446766746812     -2.25454218502863      1.07405396237653
  N   -0.08533352918556     -1.91536336341890      1.44332269945897
  H   -0.43047431635206     -2.15901776518658      2.37346114638529
  N   1.67782351716274     -1.95086698956816     -0.12907471741859
  C   2.95564681612756     -2.35301247246319     -0.35475245161986
  H   3.36050652821183     -1.68724561468060     -2.35281328091555
  Br  -1.23658416781104     -0.96410663681241      0.27054912372599
  Br  1.24363673838176     -3.37524644118321      3.74664394126218
32
R2-Br-dimer2
  N   3.52385713008190     -1.80775738310371     -2.15928085324724
  C   4.83301322061192     -2.29773648439445     -2.10306879726949
  H   5.52777353534887     -2.17050018249095     -2.94247067637578
  N   5.10266620595155     -2.90232764802178     -0.96038734832190
  C   3.92657356461310     -2.81466059392302     -0.22715667968546
  C   3.63249224288818     -3.28626424389158      1.10799396963796
  O   4.36921539740708     -3.87842401405058      1.89334940087146
  N   2.24874144359242     -2.94456184766055      1.46340135953527
  C   1.34001231458857     -2.28192684671863      0.63449910246182
  N   0.04937573918594     -2.14845388334288      1.11918577938008
  H   -0.05530216105229     -2.20675921021575      2.13958579166099
  N   1.65717203955895     -1.85666313096109     -0.57768218635347
  C   2.92557908381747     -2.13446419424782     -0.95700517908600
  H   3.07223469340786     -1.31187215150781     -2.92914070424086
  Br  -1.07521666433864     -0.79812376921795      0.38743196818197
  Br  1.75361806642306     -3.49248649949938      3.24962298141182
  N   2.78304351336720     -6.57919001566119      9.89499174177417
  C   1.47162949570709     -6.09556171756398      9.83719616465429
  H   0.77894240036274     -6.21912909108639     10.67885640666835
  N   1.19717645966021     -5.50151317544265      8.69012343607653
  C   2.37232941591839     -5.58987656876716      7.95547489036416
  C   2.66192201332259     -5.12779697656428      6.61598010053989
  O   1.92124067998550     -4.54542853789398      5.82706924872210
  N   4.04659407062544     -5.46626940326988      6.26079467983641
  C   4.95986214625059     -6.11751239142498      7.09360252150597
  N   6.25035112116114     -6.24865372857641      6.60796042007323
  H   6.35341193717804     -6.19548426128841      5.58709866931647
  N   4.64682325732912     -6.53445254164200      8.30979383310402
  C   3.37773293614369     -6.25964146526296      8.68899930950352
  H   3.23830230690285     -7.06667744500917     10.66805627608115
  Br  4.53686246756811     -4.92691230274611      4.47055665745632
  Br  7.38161205643135     -7.58989294455255      7.34639146576219
48
```

R2-Br-trimer

N	-4.08079563875857	0.67071122236680	-0.37134992021653
C	-5.39772329101106	1.13002072026490	-0.48743595988139
H	-6.09605440409780	1.08185480817110	0.35705068817076
N	-5.66645715550987	1.60323365975656	-1.69004981653200
C	-4.48471744320182	1.45432946436178	-2.40276929675781
C	-4.19272040445846	1.78831318722887	-3.78124802939572
O	-4.89135979594434	2.27680949314124	-4.64990377644572
N	-2.78072132827826	1.42622025875646	-4.07718853739194
C	-1.85427426626050	0.86636151872546	-3.18936459729189
N	-0.56746093133709	0.69650204648547	-3.64644805631835
H	-0.44196662887757	0.65657086354696	-4.66292249833545
N	-2.20060869524933	0.57135578993710	-1.93726307843483
C	-3.47994361296095	0.87468880457473	-1.59862012403631
H	-3.63238042921400	0.26033095416975	0.45064199982696
Br	-2.27294549775841	1.82326808143469	-5.89438047721839
Br	0.60991607748481	-0.45030508297950	-2.65180411475300
N	3.76533633984653	-1.96809778242761	-2.20192894947378
C	5.09070595711632	-2.40399751487076	-2.09070629941657
H	5.80486144381451	-2.27554567257847	-2.91330608151735
N	5.34715191055184	-2.95988659552020	-0.92115592239744
C	4.14706019605973	-2.89234514654902	-0.22490311194892
C	3.83213375105104	-3.32583830100287	1.11744082233734
O	4.56284895049056	-3.86258036963233	1.94511872069110
N	2.42673246685256	-3.02037277865183	1.42375578238167
C	1.50498119399660	-2.42746410800638	0.55525419677092
N	0.21200654614097	-2.32659359992870	1.01314721046991
H	0.07317022994040	-2.41587959613324	2.02580851236702
N	1.85263491717963	-2.03640544605754	-0.67302613764633
C	3.14579101219784	-2.27722700659322	-1.00713579000317
H	3.32088175467077	-1.50077892398236	-2.99560897146023
Br	-1.01247088008192	-1.15439167925796	0.13590684374482
Br	1.91171463321630	-3.53237431333062	3.21360594364461
N	2.57606816949611	-6.53227771199434	9.88604020739163
C	1.27849267736233	-6.02518222367617	9.75717832424559
H	0.53928366424999	-6.13524744790153	10.56030610179015
N	1.07701589837333	-5.42800321351828	8.59672397429914
C	2.28857159929045	-5.53825107346634	7.92707815628406
C	2.65848220057665	-5.08459951677764	6.60546002994029
O	1.96785010536703	-4.49097789710272	5.77888123053832
N	4.05410257214167	-5.44476293318841	6.32524362454859
C	4.90938889209416	-6.11400653992640	7.20566450407492
N	6.22067613065104	-6.27300184443636	6.79044447124602
H	6.38251305703489	-6.22526001591577	5.77734433982353
N	4.52254288545325	-6.52359985711714	8.40265736167790
C	3.24070282297947	-6.22488836780090	8.71409942420834
H	2.97935584757701	-7.02763483712547	10.68270614719049
Br	4.65407402779700	-4.91559044323331	4.56704352518524
Br	7.28468311194519	-7.62773109223850	7.59821298402384

32

R2-I-dimer1

N	-4.64602014974521	1.06470099760255	-0.82303541599977
C	-5.80189327780655	1.82276639029206	-1.05262401333998
H	-6.54368479147047	1.98946021764155	-0.26162648432139
N	-5.87004998192988	2.28343801031525	-2.28785587899490
C	-4.71979062548823	1.81356909179300	-2.90710286541107
C	-4.24345921466166	2.00617842967181	-4.26266278950642
O	-4.74819057768425	2.63002457782188	-5.18804445994013

N	-2.96131824929812	1.32002814679930	-4.44692946055346
C	-2.27622421154959	0.57478090630876	-3.48109321031495
N	-1.11218387886343	-0.04746270420858	-3.83042215520346
H	-0.67013443119061	0.25400485841122	-4.70438681764248
N	-2.75743475579815	0.44001531208747	-2.24431068230976
C	-3.93794453405626	1.04945943510994	-2.01107176395998
H	-4.36130251385700	0.59739674460348	0.03863329684198
I	-2.19949145330730	1.58552746605061	-6.45070909506394
I	0.20531254407588	-0.77386738638881	-2.30056242083555
N	3.73537006903171	-1.59004274821943	-2.17622767318417
C	5.02609576471339	-2.12262709412875	-2.19874155737403
H	5.68674110457780	-1.98640512184905	-3.06350316857878
N	5.32674698811197	-2.78064399257268	-1.09052192108650
C	4.19296042623393	-2.68807903777067	-0.30416163706983
C	3.93921028899388	-3.20895708156624	1.02659886580380
O	4.66724100688408	-3.85156891869436	1.76603348222428
N	2.59016727114088	-2.82044114804086	1.46500711606037
C	1.64769159655081	-2.10880902613696	0.72181989024630
N	0.38109781171734	-1.91244497997949	1.23397002798004
H	0.28941927047505	-2.34032857392367	2.16664631143397
N	1.93978017846031	-1.67026178241887	-0.50874826057447
C	3.18749406281211	-1.94546886434606	-0.96229611518971
H	3.24705572664099	-1.06810823990773	-2.90916859505687
I	-0.30470938447364	0.13209550579867	1.53897801328965
I	2.21481450283548	-3.44707964653472	3.49610570041729

32

R2-I-dimer2

N	3.90544664991342	-1.65766368631770	-2.09034892544013
C	5.15059289462392	-2.29510183931308	-2.03503302914226
H	5.86106173513787	-2.23413654036599	-2.86882713817627
N	5.34056055621745	-2.94597148189600	-0.90216941500203
C	4.17543860529407	-2.73839661420356	-0.17481884233432
C	3.81354363888229	-3.19105200279727	1.14910185061500
O	4.47997952810710	-3.87877129511624	1.92606764086870
N	2.48367934891145	-2.70883276959490	1.51323135729305
C	1.66254914394048	-1.93444608372839	0.69170650473999
N	0.39414141884356	-1.65512189642031	1.15865760733244
H	0.25670014205088	-1.74155187502726	2.17433437929955
N	2.03661872315328	-1.52252797522690	-0.51471413714005
C	3.26403227953512	-1.93567002665257	-0.89781049944427
H	3.52032305361153	-1.09952300091228	-2.85358625909801
I	-0.68966819624058	-0.03311187774105	0.34739205184693
I	1.88965316729233	-3.33040870105581	3.48633202264788
N	2.82208103052110	-6.93194711249857	10.07182881937347
C	1.58083951238379	-6.28670508183401	10.01917177225059
H	0.86841781124405	-6.34947944814285	10.85116351596021
N	1.39671423968575	-5.62632723085307	8.89082924364216
C	2.56177594841196	-5.83551802373416	8.16389547343383
C	2.92893441553101	-5.37499887518006	6.84413500430845
O	2.26856224684716	-4.67612657887642	6.07185837137213
N	4.25598040274965	-5.86316693539667	6.47834184764649
C	5.07118354140280	-6.64855587872919	7.29546665136121
N	6.33840082099823	-6.93242736843992	6.82827709957746
H	6.47787740337616	-6.83916911004723	5.81348937776127
N	4.69270591043464	-7.06663907522731	8.49835795556851
C	3.46715386751697	-6.64900607357861	8.88245832207514
H	3.20263865638735	-7.49769025645009	10.83175720145512
I	4.85539301501687	-5.23444222161075	4.50908652513988

I 7.41190959298925 -8.56580014449225 7.62997619798399
48
R2-I-trimer
N -4.62373238970876 1.08034329536566 -0.81259036100336
C -5.79053693401013 1.82155241635395 -1.04364923166723
H -6.52855323477775 1.98938652425660 -0.24935130720767
N -5.87249508669183 2.26616438440361 -2.28370164623427
C -4.71979998488754 1.80293645752065 -2.90505829024276
C -4.25485074541977 1.98239479304529 -4.26608279251505
O -4.77389785170531 2.58782460096053 -5.19650485999602
N -2.96665791764639 1.30847240133752 -4.45023567471190
C -2.26375147126110 0.58503921551063 -3.47930451286208
N -1.09290531362178 -0.02125522749303 -3.82392549688184
H -0.66713195091662 0.25413794970184 -4.71365929473416
N -2.73721303342042 0.46100110330064 -2.23761369574345
C -3.92314359403666 1.05924140664178 -2.00501861991464
H -4.32725619051100 0.62606936182246 0.05210185933087
N 3.73895321895566 -1.58306929246360 -2.17539788138984
C 5.03004236431936 -2.11522705579253 -2.18883904324719
H 5.69637050340443 -1.97725343230396 -3.04904783859956
N 5.32344207948427 -2.77423207979546 -1.07963035301778
C 4.18249185038739 -2.68238966164539 -0.29901311804058
C 3.91442409909333 -3.20995629673612 1.02341996775251
O 4.65426258763040 -3.86880505514499 1.74884269656237
N 2.57093797501670 -2.82261140587804 1.46435148113444
C 1.64318499374022 -2.10875798879285 0.71259527372722
N 0.37428486411540 -1.91042765451791 1.22321437990363
H 0.28615390492093 -2.33871471641424 2.15773883623817
N 1.93241073092297 -1.66467310494105 -0.51963878589772
C 3.18248126202934 -1.93951188062411 -0.96505493870740
H 3.25487679207651 -1.05972587707273 -2.91001027102896
N 2.63188945525958 -6.83410295988674 9.99136492923592
C 1.42572084586826 -6.13993310807371 9.83330962438112
H 0.64775082269273 -6.16259851931748 10.60662259655577
N 1.35817795672677 -5.48831918289931 8.68733293183041
C 2.56663137435764 -5.75521906021805 8.05812522527514
C 3.05710776112005 -5.32558350474680 6.77080644754039
O 2.47742610320638 -4.59610173048142 5.95779951317798
N 4.38123750563339 -5.87035592286143 6.50755038151689
C 5.09981103463325 -6.68264566076441 7.39378927563446
N 6.38017643104125 -7.03320531326433 7.02850803398049
H 6.61367198951954 -6.95735064376529 6.03074349515534
N 4.61045801182355 -7.06506708915623 8.56692021764681
C 3.37863923416068 -6.59452716765525 8.85441240118186
H 2.92640814313661 -7.40388510321513 10.78599475651604
I -2.22192936718179 1.55679573802393 -6.46376618134994
I 0.22544164102598 -0.75666979929157 -2.29524980604269
I -0.30639768756499 0.13345374910481 1.53623471010825
I 2.18386046831423 -3.48068312482173 3.48183105906469
I 5.18098488289096 -5.31901863481409 4.58444488941114
I 7.34734954585413 -8.64978860250093 7.97028765417411
32
R2-At-dimer1
N -4.55457932966125 1.15586052140090 -1.06475401575313
C -5.71761071703971 1.89934347373101 -1.28970474507083
H -6.49232614567831 2.00001443149700 -0.51947359881722
N -5.75490130138156 2.43816334013276 -2.49873027657188
C -4.57573930180776 2.03991749285819 -3.10270230292446

C	-4.04726572684533	2.32346499081935	-4.42747253216768
O	-4.54733292422554	2.99026887661441	-5.32853487264915
N	-2.75045450214496	1.69099945631979	-4.60639575453785
C	-2.07351132848569	0.91268268599623	-3.66705719418899
N	-0.86577219316513	0.37820758772677	-3.95531032110435
H	-0.43707200808320	0.61280038254075	-4.85543789180274
N	-2.60820002737093	0.67763140038267	-2.45567103125929
C	-3.80409386660780	1.23578267828239	-2.22521755709501
H	-4.27546830205020	0.64754287174891	-0.22257596679512
At	-1.90440160008875	2.13248633598866	-6.67270278665084
At	0.24256632753580	-0.77465875375552	-2.34313386715063
N	3.69947382453033	-1.74542723187153	-1.55366380904762
C	4.97304960883489	-2.18867377162997	-1.19337584947535
H	5.84900798038463	-1.98490693569291	-1.82067211546242
N	4.97706706357058	-2.85419005079313	-0.04793255294138
C	3.66014539490145	-2.85533971312837	0.36807404199110
C	3.05929043426962	-3.42124405334025	1.56645180376526
O	3.59892292751766	-4.02900436697074	2.48079815618687
N	1.63170225396905	-3.14518190068727	1.59016777325126
C	0.88321424979968	-2.47854105504957	0.61630775532653
N	-0.46841875615556	-2.33634103725368	0.71084230974033
H	-0.82287232383573	-2.86573068340881	1.52165868572596
N	1.50405264030190	-1.98522159807468	-0.48546897037259
C	2.84384888424193	-2.16523135380005	-0.55664987798981
H	3.41787992178021	-1.21712418953517	-2.38453833840260
At	-1.25441521346644	-0.14897380842440	1.42585159365985
At	0.70429911445611	-3.89901136462373	3.51622498458379

32

R2-At-dimer2

N	3.57538461786264	-1.79066567646080	-2.00011192679310
C	4.86061730147410	-2.34379367404472	-1.95872599885152
H	5.56922648713726	-2.20477900636524	-2.78469594745372
N	5.08988178814323	-3.02139238753543	-0.84858607021049
C	3.90970726018655	-2.92143513348115	-0.12306725528242
C	3.57090154480855	-3.43874408736269	1.18247119171840
O	4.29554150813855	-4.11256008545832	1.92936558627370
N	2.22545411900213	-3.05790421396246	1.56280254456078
C	1.35325950936218	-2.31991286051412	0.76434055958103
N	0.06544732877764	-2.15102182110940	1.22888835228037
H	-0.04967309057920	-2.27499032037332	2.24546346730659
N	1.69645588713933	-1.84231802107426	-0.43193048297518
C	2.94802977006543	-2.15549356923717	-0.82287702460918
H	3.15648638965549	-1.23359532251230	-2.74610955601659
At	-1.11537267604205	-0.38596994098550	0.52557583465710
At	1.67258787952830	-3.80553302890393	3.63351723469835
N	3.15304176364329	-6.89642987097540	10.27050534454393
C	1.84583516084153	-6.40175736303409	10.19342838252593
H	1.16487781993480	-6.45087925544246	11.05220439871633
N	1.55873205205635	-5.89876266013152	9.00657642838483
C	2.71800908334442	-6.06882695600465	8.26071486775693
C	3.01522651993336	-5.68892870778510	6.89906498210831
O	2.25061162214434	-5.13253185883061	6.09724971975228
N	4.37817765127776	-6.03024609709246	6.54544865092788
C	5.27917456259522	-6.67665051358333	7.39058920225680
N	6.49741605651816	-7.03658140482507	6.85421938244918
H	6.77468947121774	-6.51343701297840	6.01090651362977
N	4.98298829016057	-7.00542977494308	8.64802197484769
C	3.72965506541873	-6.68907888214284	9.03084031743837

H	3.61255771492962	-7.33076002721851	11.07196034920075
At	4.90815263003410	-5.39625301153464	4.43019522829744
At	8.24848939328989	-7.29742991209705	8.22036525827943

48
R2-At-trimer

N	-4.17350422402105	0.92468539051299	-0.44463082984765
C	-5.37390039390070	1.64410718066825	-0.45553537287098
H	-5.98532486344836	1.75280206580350	0.44868866131709
N	-5.65604240430191	2.14961408634507	-1.64505455215783
C	-4.60728810139918	1.75300992345155	-2.45885281219315
C	-4.35019532691338	2.01157963683715	-3.86544210565853
O	-5.02713852866318	2.64884543512939	-4.66773665274497
N	-3.09936317850105	1.39412825259168	-4.27799134871265
C	-2.23576096018314	0.65121809424021	-3.47141299556873
N	-1.09559281237451	0.12737770454392	-3.97772545811553
H	-0.83733350653018	0.37041677997517	-4.93794084096159
N	-2.52297141208852	0.44009448374645	-2.17554714147288
C	-3.66483286030544	0.98524194227316	-1.73052205149835
H	-3.72446753565796	0.44457110383147	0.33814168793209
At	-2.67432979323496	1.80900094923165	-6.47638615723182
At	0.35233606937345	-0.89703504403347	-2.55538784132022
N	3.92739512413902	-1.74465647944875	-2.36597584252686
C	5.25742428339362	-2.14528804058164	-2.22589238877123
H	5.99514932079759	-1.96551527993310	-3.01734251973688
N	5.49353792284366	-2.73988783432435	-1.06633504540372
C	4.27544997086147	-2.73669883847748	-0.40901993042528
C	3.92011156838438	-3.24171376612775	0.90472810181149
O	4.66555996284391	-3.79652997031635	1.71565240082716
N	2.52231626981372	-2.98712687826193	1.19689591035170
C	1.60276399263465	-2.39241136030764	0.33770450453755
N	0.28393364986043	-2.26969070444654	0.68495984035767
H	0.11484621899785	-2.75644852360297	1.58021465216542
N	1.97745595911500	-1.95619534220133	-0.89184530962785
C	3.28451201398943	-2.11658935484628	-1.20336250122712
H	3.48149661799208	-1.27136835350097	-3.15705685233842
At	-0.41983507445589	-0.08463607459452	1.37763988498917
At	1.99107240543796	-3.65767076867061	3.30413002695028
N	2.36820182147455	-6.71238140734376	9.89691769239127
C	1.10437230599961	-6.18206373261149	9.60820104672283
H	0.27095690011444	-6.27518021609768	10.31548548024709
N	1.05690637607074	-5.58531399642903	8.43146470225076
C	2.33942612279730	-5.71801219166728	7.91734243260279
C	2.88674586124418	-5.28209177985026	6.65618019365801
O	2.27436987038511	-4.65890213414717	5.77218131174966
N	4.27332815376526	-5.66430755889600	6.51894271140585
C	5.01057866859698	-6.33977304660560	7.49687632024761
N	6.35214513268952	-6.53074930484990	7.25980368461562
H	6.64277373114503	-6.47539835177360	6.27365408581954
N	4.47522401643462	-6.73868900542146	8.64918752730211
C	3.17681159068194	-6.42013094426866	8.81528673638916
H	2.65995453013948	-7.21137546264235	10.73863354939498
At	5.19387287068478	-5.05748074092965	4.53347600491030
At	7.42365935327771	-8.17223300197204	8.32060603546499

64
G4-1

N	-1.87124500	0.82698500	6.52368300
C	-3.06849700	0.48487600	5.89715600

H	-4.02923700	0.51572500	6.42378900
N	-2.88369400	0.13201600	4.63351200
C	-1.51480300	0.24729100	4.41417800
C	-0.72715500	0.00857200	3.22839600
O	-1.11612300	-0.36575500	2.10928200
N	0.64784500	0.26372300	3.45976600
H	1.25944900	0.21127700	2.60188600
C	1.19521200	0.67314200	4.66455800
N	2.53541600	0.87013600	4.71400600
H	3.21213600	0.53444300	3.99316400
H	2.89700000	1.12769500	5.63137600
N	0.46182300	0.89599500	5.77179000
C	-0.85452300	0.68049000	5.58610800
H	-1.74443400	1.13903100	7.48689400
N	6.53349600	-0.73275700	1.89369500
C	5.89801500	-0.37914000	3.08282000
H	6.42803200	-0.36157300	4.04204900
N	4.62187300	-0.07776600	2.89230200
C	4.40331500	-0.24063600	1.52810100
C	3.20705900	-0.06990600	0.73810900
O	2.07506400	0.27009700	1.12038200
N	3.44510600	-0.35368600	-0.63033700
H	2.58546200	-0.34464100	-1.24236500
C	4.66342000	-0.73200600	-1.16976800
N	4.71775700	-0.96271300	-2.50473900
H	3.99077700	-0.65382000	-3.18781300
H	5.64460400	-1.19316900	-2.86068300
N	5.77930600	-0.89500300	-0.43384900
C	5.58865300	-0.64984300	0.87661600
H	7.50674300	-1.01467400	1.77275200
N	1.89251700	0.65057600	-6.49425600
C	3.07882900	0.28295400	-5.86160800
H	4.03929500	0.27042100	-6.38952100
N	2.88441000	-0.03628200	-4.59042500
C	1.52046900	0.12870600	-4.37218500
C	0.72647500	-0.05856000	-3.18097900
O	1.10369500	-0.41932100	-2.05344800
N	-0.63977500	0.23501100	-3.41808600
H	-1.25293400	0.22500100	-2.55949800
C	-1.17502500	0.63437100	-4.63151300
N	-2.50895900	0.87182400	-4.68403000
H	-3.19329200	0.55213000	-3.96309600
H	-2.86428800	1.11401200	-5.60810900
N	-0.43606400	0.81045900	-5.74326900
C	0.87306400	0.55771900	-5.55289100
H	1.77446900	0.94635200	-7.46371900
N	-6.52059300	-0.69042100	-1.85303800
C	-5.88364000	-0.35804000	-3.04748800
H	-6.41046600	-0.36557500	-4.00860900
N	-4.61011300	-0.04433500	-2.85967900
C	-4.39481400	-0.17677100	-1.49168400
C	-3.20273400	0.01943800	-0.70163700
O	-2.07153700	0.35992600	-1.08685600
N	-3.44308600	-0.23759800	0.67137200
H	-2.58313700	-0.22178600	1.28239800
C	-4.66004700	-0.61369200	1.21540000
N	-4.71621300	-0.81706100	2.55450900
H	-3.99108200	-0.49419800	3.23280200

```
H  -5.64122500  -1.04896600   2.91412700
N  -5.77276700  -0.79871200   0.47974800
C  -5.57953100  -0.57977700  -0.83496800
H  -7.49233500  -0.97618600  -1.72911400
64
G4-2
  C   2.16748100169149      5.35702377351588     -0.00000014932829
  C   0.94617048696471      4.64646067603288     -0.00000020745789
  N   -0.13291116039171      5.52391866232098     -0.00000023711723
  C   0.39855795171286      6.73750484743802     -0.00000022622456
  N   1.79123387086493      6.69599748443451     -0.00000016824586
  N   3.41971907338246      4.86494905639116     -0.00000009373494
  C   3.45920257329553      3.51821418418118     -0.00000010787798
  N   2.32353242918178      2.72352872826395     -0.00000018074128
  C   0.98672647166232      3.20298878637836     -0.00000022213282
  O   0.03469450734825      2.40638428425714     -0.00000026551238
  N   4.66653434674975      2.90735136551822     -0.00000005534022
  H   -0.15578086278789      7.68301983455538     -0.00000024875820
  H   2.41989403624943      1.67454494621311     -0.00000019490848
  H   5.46830012052890      3.53567550278923      0.00000001655009
  H   4.84565517961312      1.87876377665514      0.00000000312678
  H   2.43360761245128      7.48884332509938     -0.00000014485080
  C   -5.35702498286719      2.16748009641338      0.00000030975858
  C   -4.64646179690869      0.94616962859401      0.00000006668860
  N   -5.52391969479191     -0.13291209693251      0.00000014477458
  C   -6.73750592188571      0.39855692293033      0.00000042471896
  N   -6.69599866823882      1.79123285778429      0.00000054186386
  N   -4.86495033536569      3.41971819300914      0.00000033091334
  C   -3.51821543541858      3.45920177807397      0.00000009740595
  N   -2.72352988370084      2.32353170390682     -0.00000013791756
  C   -3.20298991171381      0.98672572623333     -0.00000016727035
  O   -2.40638538873475      0.03469379048974     -0.00000035303857
  N   -2.90735261161006      4.66653352868761      0.00000010098589
  H   -7.68302086283256     -0.15578197249339      0.00000055793832
  H   -1.67454594269943      2.41989339232100     -0.00000021475578
  H   -3.53567674093649      5.46829931291280      0.00000032656963
  H   -1.87876482976524      4.84565426829855     -0.00000004530093
  H   -7.48884453708676      2.43360655935509      0.00000075975795
  C   -2.16748194499488     -5.35702483973230     -0.00000017314949
  C   -0.94617136247415     -4.64646184430977     -0.00000017744071
  N   0.13291017439549     -5.52391996822163     -0.00000015274023
  C   -0.39855904332996     -6.73750611275067     -0.00000013607806
  N   -1.79123494799573     -6.69599860100893     -0.00000014148433
  N   -3.41971995485878     -4.86495000460207     -0.00000018168175
  C   -3.45920333670785     -3.51821511359047     -0.00000020710449
  N   -2.32353312709102     -2.72352975325973     -0.00000022919646
  C   -0.98672721065354     -3.20298992272895     -0.00000020763254
  O   -0.03469525192983     -2.40638536616044     -0.00000021552216
  N   -4.66653501882599     -2.90735213898110     -0.00000022220548
  H   0.15577967894702     -7.68302114758326     -0.00000012043322
  H   -2.41989464846978     -1.67454587697754     -0.00000028163873
  H   -5.46830087577111     -3.53567617630763     -0.00000019146994
  H   -4.84565566278696     -1.87876439996264     -0.00000009368376
  H   -2.43360878699534     -7.48884436070121     -0.00000013175286
  C   5.35702587392802     -2.16747860453696      0.00000026910381
  C   4.64646204471476     -0.94616850936630      0.00000009068577
  N   5.52391937379936      0.13291368954058      0.00000013776010
  C   6.73750588262438     -0.39855468685896      0.00000032435554
```

N	6.69599936143477	-1.79123065359814	0.00000041676555
N	4.86495189219687	-3.41971695197287	0.00000029646325
C	3.51821699324517	-3.45920125220936	0.00000012877565
N	2.72353081975457	-2.32353161906436	-0.00000004435965
C	3.20299017633460	-0.98672538945110	-0.00000007206905
O	2.40638519645842	-0.03469382547361	-0.00000021676838
N	2.90735472932142	-4.66653324824638	0.00000012535343
H	7.68302053831065	0.15578470494884	0.00000040635834
H	1.67454674380477	-2.41989384680983	-0.00000012839650
H	3.53567921143831	-5.46829875583866	0.00000029294173
H	1.87876682125485	-4.84565441251857	0.00000000746498
H	7.48884556696079	-2.43360393529464	0.00000056824123

64

Cl-G4-1

H	-1.02626628581307	-3.11669730300382	6.73895462135718
C	-0.64584948115100	-1.42932951839222	5.46963559007455
N	0.24467610182841	-1.88653247757742	4.55821363107484
H	1.89365456900292	-2.32851086624168	3.01213735455191
Cl	1.15590814713912	-1.41962511241202	1.03192474371980
N	1.67904295185756	-1.34403012037294	2.80003855752624
C	0.65743967144836	-0.97439698090235	3.68495699873582
Cl	0.87298462825515	1.49044344919201	2.56721347203025
N	0.18663287057144	0.32804004458288	3.67681451487990
O	-1.31075663555059	1.98894326727211	4.39097914270330
C	-0.88163957128808	0.85892605852677	4.55087074435327
C	-1.24551820871194	-0.15114394267174	5.52667510749962
N	-2.16535604015130	-0.08511152659208	6.55813083699667
H	-2.72674339889799	-1.60022007704260	7.98672051662405
C	-2.13082439921746	-1.28198501111796	7.12249926656228
N	-1.22537310504833	-2.14337529814487	6.50358026629353
H	5.76101020982389	-1.72322845962741	2.81233514913330
C	4.67237747557493	-0.49479146489447	1.42932051850282
N	4.59997214957147	-1.23890119262223	0.30182890844909
H	4.47132425632558	-2.18324654836522	-1.80359362112508
Cl	2.25752513893498	-1.96012317267880	-2.37105996730237
N	3.94520613272301	-1.30840791432833	-1.94430360688490
C	3.97489831634169	-0.64752560696831	-0.70847344069771
Cl	2.66459106974918	1.34545929967371	-2.01737205817635
N	3.42113075560014	0.62068734910485	-0.61506783614818
O	2.74064960250461	2.49120373811257	0.62785630635210
C	3.35339235471815	1.43739222798942	0.61077348514863
C	4.09749754749594	0.77337006355959	1.66656037326364
N	4.31725093573103	1.17713360439790	2.97051807954424
H	5.37185244110135	0.16456207556165	4.55559767956633
C	5.00944238861873	0.19195393515946	3.52071888949891
N	5.25922215934157	-0.85274854297171	2.63161465068604
H	0.95260914214244	-3.29421976060285	-6.67700586003004
C	0.61770044327069	-1.56919522603202	-5.44601847799702
N	-0.28115523770600	-1.98314783732545	-4.52211269668742
H	-1.93516197047222	-2.34934140087558	-2.96215857390125
Cl	-1.17170574027889	-1.41065700218163	-1.00585189457896
N	-1.69722958320893	-1.36533379463548	-2.77424680057205
C	-0.66943992053940	-1.04100343182407	-3.66957169266672
Cl	-0.82292089664808	1.45423476050299	-2.61041123060601
N	-0.16745977358913	0.24946509143024	-3.69321965299093
O	1.36753812855449	1.85650081693417	-4.44957485513223
C	0.91136380315548	0.73350992059133	-4.58154981112041
C	1.24820878316103	-0.30771861924504	-5.53416236439165

N	2.16638970810281	-0.28818833734687	-6.56904334609408
H	2.68658342617689	-1.84940920426183	-7.96317372810826
C	2.10102095434586	-1.49658475861480	-7.10534678729208
N	1.17658104902516	-2.32104158345898	-6.46444530658255
N	-5.26017966910687	-0.79628719342552	-2.62651198145854
C	-4.98654047812525	0.22209494207667	-3.53874178025970
H	-5.34832208391855	0.17909143231363	-4.57333732584525
N	-4.27383518229940	1.20438672663247	-3.00992924313775
C	-4.06460686585799	0.82578469797413	-1.69664883319444
C	-3.30744123160722	1.49730308686732	-0.65505731984871
O	-2.67201487403046	2.53684904761604	-0.69482019943268
N	-3.39388674721778	0.70990691141549	0.58889025877926
Cl	-2.62054042021718	1.44865686066748	1.97460263815137
C	-3.97639833039774	-0.54275826186942	0.71052397705726
N	-3.96424959247070	-1.17543934922461	1.96130986836561
Cl	-2.29336327725738	-1.85525884422878	2.40881122441317
H	-4.50991834942193	-2.04107939834525	1.83928137390990
N	-4.61311212609250	-1.14314276773374	-0.28717372251694
C	-4.66742720332719	-0.42376053914734	-1.43156025710012
H	-5.78044063257359	-1.65964136884738	-2.78834447392451

64

Cl-G4-2

N	-2.34960379570621	-0.18440001757676	7.89086574128327
C	-3.61205054045459	-0.34093684180513	7.32880931280079
H	-4.48809620110751	-0.63687722644980	7.91625780168803
N	-3.60282368601905	-0.08612087049968	6.03034101390742
C	-2.29643996372291	0.25440766892535	5.71326412765319
C	-1.71698272611758	0.64117790690539	4.43972386001219
O	-2.22981145915417	0.74683719156779	3.34310889864270
N	-0.28017132905673	0.96169832061418	4.64658147354801
C	0.42462420274819	0.83160813786231	5.84160404876096
N	1.74889209596305	1.22158165768114	5.91946453112957
H	2.07174728177068	1.04269898969360	6.87821599248234
N	-0.16412609999675	0.42558410435630	6.96954988094478
C	-1.49074113768833	0.19957740970823	6.86903159439659
H	-2.08050777989211	-0.33054232088637	8.86475128940239
Cl	2.96352950901133	0.40934995686270	4.80165044620625
Cl	0.50675676163107	1.62084476217862	3.23228292414656
N	5.93271187730829	-1.95884080907079	1.98956386478356
C	5.35076883164943	-1.65060435096102	3.21375935192403
H	5.56555936852243	-2.21471154852280	4.12805897651653
N	4.52824936443875	-0.61859623189488	3.11953524226052
C	4.55511904104295	-0.22561136839718	1.78909340842798
C	3.86572172226195	0.86399276514600	1.11930824758502
O	3.09704471330914	1.69975744784274	1.55435642352911
N	4.27921029767741	0.88269708318578	-0.30681065270516
C	5.13760046385633	-0.02114206028084	-0.92978304442793
N	5.47996182746203	0.16036360472268	-2.25863128293595
H	6.13556146589342	-0.59170050331829	-2.50599965024371
N	5.71668654479146	-1.01737953402652	-0.25844253617154
C	5.43516263720039	-1.05445381429587	1.06129179140496
H	6.59229254204940	-2.71101696381084	1.78533006670260
Cl	3.67462479350500	2.25989802661051	-1.19024853097338
Cl	4.09149377887339	0.00055035001225	-3.48363814312919
N	0.93937018983726	-0.29160522332910	-6.95738756585974
C	2.19877350294013	-0.46538058812012	-6.39357383363881
H	3.06846645608480	-0.78683738417890	-6.97704604759129
N	2.19539470935407	-0.19213304244681	-5.09883007999407

```
  C   0.89623388984091     0.17867982018780    -4.78613193467680
  C   0.32542720981721     0.59484007827726    -3.51799958218545
  O   0.84095289838482     0.70581420942969    -2.42319470833479
  N   -1.10505937423559     0.94027639466902    -3.72869728130318
  C   -1.81287112869854     0.80807034701508    -4.92168568827655
  N   -3.12904898553740     1.22365128676990    -5.00491682590691
  H   -3.45549608604896     1.03793278482919    -5.96115216389469
  N   -1.23292691198393     0.37489546072544    -6.04405645795686
  C   0.08890910790526     0.12365883208875    -5.94074457153266
  H   0.66695306593232    -0.44588463633128    -7.92907438932240
  Cl  -4.36000552475864     0.45129101007651    -3.87656410059583
  Cl  -1.87863574934781     1.63173561509669    -2.32243754337209
  N   -7.38366185489673    -1.82130503352958    -1.04452504501686
  C   -6.79430960913376    -1.53656462628467    -2.27093210639854
  H   -7.02312366771528    -2.10174841279933    -3.18115646647899
  N   -5.94645302211366    -0.52461238247410    -2.18410520836935
  C   -5.96359175072344    -0.12124451173581    -0.85667182167113
  C   -5.24847526203485     0.95684947592778    -0.19521832818186
  O   -4.46050440211109     1.77105678074107    -0.63663242622820
  N   -5.66127400964334     0.99635027366826     1.23057665499369
  C   -6.54129557724420     0.11831272312880     1.86026676591314
  N   -6.87902959299423     0.31825307884350     3.18761975281324
  H   -7.55263924702450    -0.41567783201024     3.44093012023372
  N   -7.14449214296642    -0.86866995642426     1.19647859533388
  C   -6.86390045338713    -0.92265693832467    -0.12290470977219
  H   -8.06297752034367    -2.55430657565922    -0.83519782252712
  Cl  -5.02483891399231     2.36610861683103     2.10312235670903
  Cl  -5.49471964521143     0.13531743326353     4.41399499353723
64
Cl-G4-3
  N   -2.03144405404214     0.57419978462537     6.62777275547600
  C   -2.71414416039446    -0.63805754521011     6.54311903148189
  H   -3.57449711237742    -0.86264077184247     7.18479186831782
  N   -2.21056447252456    -1.43693717344958     5.61495712935402
  C   -1.15681704191303    -0.72509175603973     5.05804101924553
  C   -0.28963918604767    -1.08115651658521     3.95113945276180
  O   -0.19979475331891    -2.10818001681607     3.31130297568246
  N   0.52260115239806     0.11548186022719     3.58038797044445
  Cl  1.14600156874043     0.03362447142690     1.93662969867952
  C   0.58622319335039     1.30908078927586     4.28957708361865
  N   1.34199518483065     2.36676250796658     3.80835294951578
  Cl  3.09790845072955     2.11896919927317     3.47687413907914
  H   1.26516602067458     3.15485152645310     4.46259969359540
  N   -0.14540307415461     1.52352586201026     5.37930740191122
  C   -1.03650186113093     0.54282729971211     5.66345862306263
  H   -2.22926555914870     1.36079470055830     7.24833053482837
  N   6.64443174011593    -0.52132113680623     2.02053005252014
  C   6.53828606039867     0.69915001126294     2.68544091200817
  H   7.18376942709272     0.95223439895916     3.53497871810904
  N   5.58635457420435     1.46809986181742     2.17970084944708
  C   5.03520593951295     0.72696484190069     1.14326565296482
  C   3.91245021199815     1.04373332778687     0.28106801993176
  O   3.24941376064183     2.05480324269161     0.17946005115608
  N   3.56014976731225    -0.17495571729630    -0.50610963934852
  Cl  1.90855974848190    -0.14059566755086    -1.11301133803724
  C   4.29800099465513    -1.35140363837816    -0.56114085582017
  N   3.83564167437823    -2.43061073805138    -1.29816126027699
  Cl  3.47797754150054    -2.21143276170467    -3.05341613343223
```

H	4.51136631687553	-3.20032145012033	-1.21977255713063
N	5.40026071596877	-1.52852026021074	0.16172216583666
C	5.66941725349440	-0.52811656792660	1.03557849695289
H	7.28586886254259	-1.28964694059454	2.22355651915520
N	2.03982065339235	0.39967520707326	-6.60372291147955
C	2.69989634606644	-0.82248926585945	-6.48828677741041
H	3.55394993908405	-1.08041729686809	-7.12579712744121
N	2.18411782015624	-1.58648585660655	-5.53773863307224
C	1.14566428763597	-0.84019945534365	-4.99734384936929
C	0.27481739247287	-1.14999271184387	-3.87953588392619
O	0.16735425272599	-2.15749563676854	-3.21198732754135
N	-0.51324709979851	0.07156513688212	-3.53867834821326
Cl	-1.13346489295488	0.04440304152392	-1.89194661671666
C	-0.55678509155918	1.24661266126543	-4.27965140572151
N	-1.29105406599860	2.33114675224263	-3.82595371343306
Cl	-3.04903163965283	2.12596965194138	-3.47736962067460
H	-1.20267724848124	3.09903045210719	-4.50246667674263
N	0.17538149829487	1.41753681273424	-5.37681182619293
C	1.04719910634060	0.41301877987775	-5.63650417878310
H	2.25032424644047	1.16515117979674	-7.24615456591738
N	-6.63075082264189	-0.42347446615374	-1.97954058927909
C	-6.50303642571370	0.78400437065559	-2.66392480833391
H	-7.14189225256462	1.03364550289371	-3.51943897825165
N	-5.54005488261156	1.54553744056759	-2.16789080181823
C	-5.00378462982194	0.81270042580689	-1.11789236959773
C	-3.87817765810560	1.12528210763146	-0.25780375494529
O	-3.19882545466447	2.12695684298036	-0.17154818766208
N	-3.54866705213598	-0.08516197523467	0.55129940867579
Cl	-1.89816009350405	-0.06859714239328	1.16251323391333
C	-4.30556587459649	-1.24864403214053	0.62286171558175
N	-3.86276548210642	-2.32258393734821	1.37937074290000
Cl	-3.51110572443349	-2.07929760202981	3.13254631483851
H	-4.54983281269423	-3.08315444856528	1.31053761041252
N	-5.40822154127721	-1.42005239046023	-0.10069314374560
C	-5.65863882853201	-0.42992309686568	-0.99168146800549
H	-7.28434685360607	-1.18435707886321	-2.17163144313808

64

Cl-G4-4

N	-11.20821850962558	-8.27054056945251	-1.07205399518447
C	-11.61156803294244	-6.94428154662673	-0.90745305933355
H	-12.63563321532312	-6.61737678893907	-1.12114428567239
N	-10.63164641897022	-6.16597847444064	-0.48361688788162
C	-9.52912884035602	-7.00067880450769	-0.35663951001877
C	-8.16384820350918	-6.82111836564827	0.01447573403327
O	-7.76924757209636	-5.59395260824990	0.37192299412547
N	-7.33099614993425	-7.85812088509610	0.00414714514570
C	-7.78292818997051	-9.08656740407278	-0.36260433759204
N	-6.91131082867852	-10.15269617401467	-0.27698428344865
H	-7.21703781135590	-10.99188077446881	-0.77696429519771
N	-9.04461231584879	-9.39381101820123	-0.76430748573802
C	-9.86445596727124	-8.33772756610331	-0.72544704994998
H	-11.77614121599413	-9.05670163663661	-1.38926504321611
Cl	-5.91839046049432	-5.47071774842089	0.54361095146387
Cl	-5.15440274013900	-9.92704901764218	-0.32670751929355
N	-0.41071048256369	-10.34709319186705	-1.07904680570716
C	-1.73612263164917	-10.75048905742813	-0.90825502535862
H	-2.06489472145044	-11.77332860836842	-1.12488485403162
N	-2.51125528240196	-9.77212036849546	-0.47510908354030

C	-1.67517425662723	-8.67061887533764	-0.34789209353282
C	-1.85198804199535	-7.30665095116891	0.02909455604066
O	-3.07703553865879	-6.91308627576617	0.39534218796808
N	-0.81484544679847	-6.47368571098587	0.01589054602117
C	0.41091950955053	-6.92506840278405	-0.36091401994964
N	1.47770922321782	-6.05415144097847	-0.27924796980981
H	2.31452858555451	-6.35844948524422	-0.78396052981140
N	0.71532666430494	-8.18529951144095	-0.76952682265975
C	-0.34063867204684	-9.00499045481714	-0.72660306081426
H	0.37317336198041	-10.91396538950363	-1.40369688626472
Cl	-3.20294039579842	-5.06193742680099	0.56052885591083
Cl	1.25292438154319	-4.29707503855828	-0.31808983717800
N	1.67256288940164	0.44155555935703	-1.08027695637839
C	2.07717913931931	-0.88187641111758	-0.89734696210652
H	3.10016855787592	-1.21183295604748	-1.11146705573127
N	1.09967877285527	-1.65386732748640	-0.45673361405842
C	-0.00268180261072	-0.81768520574929	-0.33709041914349
C	-1.36654522487947	-0.99265812103258	0.04125615152305
O	-1.75850229938613	-2.21454739619293	0.41938524553562
N	-2.20069141152627	0.04355257088336	0.01805069144070
C	-1.75064682613233	1.26585403126439	-0.37082236786401
N	-2.62239251171566	2.33299194644498	-0.29991959221866
H	-2.31765931094659	3.16469756213486	-0.81280409109865
N	-0.49052505848530	1.56796572645987	-0.78189967878590
C	0.33046780271047	0.51352643836334	-0.72816318381899
H	2.23865775706347	1.22294886014697	-1.41220013915923
Cl	-3.61087113695371	-2.34622584601304	0.56633771069071
Cl	-4.37948716309717	2.10772487447460	-0.34207831921431
N	-9.13029461887806	2.53499052828878	-1.06384020386533
C	-7.80398356686482	2.93854816970763	-0.90010288431190
H	-7.47774949653465	3.96322346095774	-1.11189874458943
N	-7.02468360116782	1.95791159347611	-0.47980538556695
C	-7.85873753106829	0.85468377318412	-0.35443082659399
C	-7.67777110741078	-0.51134653475181	0.01314010231619
O	-6.45009751101044	-0.90576685139259	0.36830297636250
N	-8.71428071645429	-1.34494524807335	0.00135910673002
C	-9.94343397508645	-0.89258742624558	-0.36221278240817
N	-11.00927118337569	-1.76476035133594	-0.27743362074155
H	-11.84935814450774	-1.45779185074462	-0.77511258334197
N	-10.25215959767455	0.37003568370403	-0.76003868234620
C	-9.19633399228596	1.19032707261804	-0.72047306853514
H	-9.91712079438018	3.10336100345637	-1.37857859401464
Cl	-6.32491026335122	-2.75726359701415	0.53486392274037
Cl	-10.78413284909321	-3.52150516765805	-0.33237638097011

64

Br-G4-1

H	-1.97937798389673	-2.37025582824222	6.76048565019752
C	-1.05020435531232	-0.96302135517571	5.43240975131937
N	0.00756021644109	-1.62169054966599	4.91582256263819
H	1.93029821187705	-2.44308727407234	3.94516024979933
Br	1.68823124050034	-1.94227110299225	1.54532104002685
N	1.89044770178561	-1.49892188228543	3.53250675453586
C	0.74776668392487	-0.90566741400168	4.06849765257382
Br	1.64509968262466	1.41741808644452	2.59464904534617
N	0.45194258573032	0.40883834145452	3.73105515396056
O	-0.99996897550234	2.24034792190433	3.69428163143618
C	-0.73975735126829	1.12614787537033	4.14987068328712
C	-1.49609974195997	0.33900354330146	5.10218225250395

N	-2.66938318110072	0.64221130332350	5.76887361490024
H	-3.79896901122217	-0.55833360107097	7.15906870137513
C	-2.93626054879666	-0.43392815754766	6.49263209802509
N	-1.98752358292805	-1.44405518247688	6.33092055035150
H	6.71822772692554	-2.32562502578008	2.03564728831801
C	5.39746261017987	-0.93182842082568	1.07610985128366
N	4.90232511521349	-1.59964165556946	0.01381733652784
H	3.95923607413131	-2.44088785349930	-1.91463897370031
Br	1.55419666818779	-1.94822869925231	-1.69840699000630
N	3.54212156824949	-1.49817613460287	-1.88762282193496
C	4.06173163525129	-0.89350363965273	-0.74322126642372
Br	2.58760044834049	1.41504126538788	-1.67821471012179
N	3.71239802067987	0.42102072951874	-0.46132836574873
O	3.63745682560259	2.25855391052639	0.98113515160174
C	4.10570272262607	1.14681005840000	0.73349451454871
C	5.05067428182714	0.36998977587789	1.50982852273381
N	5.69436277535931	0.68332350258224	2.69313405993876
H	7.07267265460461	-0.50221261223491	3.85277717097656
C	6.42076285476335	-0.38650708834591	2.97786393053356
N	6.28274884980847	-1.40215790993633	2.03133060558885
H	1.97766186109835	-2.37326716427821	-6.75955890027076
C	1.04954097371571	-0.96482271993213	-5.43204338274175
N	-0.00873367286068	-1.62248005358567	-4.91520153232624
H	-1.93202579839184	-2.44206926410171	-3.94411542485336
Br	-1.68886397669377	-1.94083445021658	-1.54453574473692
N	-1.89144378468930	-1.49781042143402	-3.53173172793025
C	-0.74839241586820	-0.90557923616159	-4.06815644209225
Br	-1.64380990359320	1.41873748159010	-2.59504251719638
N	-0.45166477650065	0.40884955785726	-3.73126280010159
O	1.00130664271017	2.23949266973731	-3.69563053627198
C	0.74049611408755	1.12517051643477	-4.15054071472208
C	1.49636763385878	0.33702415757912	-5.10235565443554
N	2.66990895449119	0.63908326857605	-5.76913066781885
H	3.79866960574299	-0.56288513468525	-7.15875781302107
C	2.93602480918100	-0.43755680122742	-6.49241200864569
N	1.98651355057995	-1.44690996834886	-6.33034826491367
N	-6.28227827616744	-1.40168095227611	-2.03178293667760
C	-6.41974746760747	-0.38617492015420	-2.97854044293151
H	-7.07135809346434	-0.50189068059355	-3.85367504784957
N	-5.69319075177452	0.68353490186612	-2.69375918953379
C	-5.04992238622498	0.37024772934223	-1.51020834971559
C	-4.10515015320061	1.14710859419177	-0.73371631454928
O	-3.63680351225321	2.25883635087410	-0.98098011842246
N	-3.71214906650065	0.42116645414661	0.46126173126391
Br	-2.58704983018996	1.41480868175056	1.67811440354048
C	-4.06194965317032	-0.89320174125431	0.74330380706271
N	-3.54269592422533	-1.49786798302128	1.88789734235774
Br	-1.55485852727959	-1.94888184729054	1.69870579874705
H	-3.96007749165174	-2.44046604990114	1.91496158160774
N	-4.90261585501579	-1.59919252326921	-0.01379781152329
C	-5.39717243020516	-0.93141227387328	-1.07635333472614
H	-6.71801882058433	-2.32502510520202	-2.03605068296463

64

Br-G4-2

N	-2.40151100	-0.62653500	7.80141300
C	-3.65846900	-0.58349800	7.21615200
H	-4.56689100	-0.93299900	7.71689800
N	-3.59825200	-0.07165800	5.99439200

C	-2.26596400	0.23543300	5.75581800
C	-1.63045400	0.83234900	4.59709900
O	-2.13284800	1.20242400	3.55048900
N	-0.18726700	0.98660000	4.85393800
C	0.48757700	0.57697200	6.00777900
N	1.82572200	0.83240100	6.17322500
H	2.09789900	0.46736600	7.09350500
N	-0.16278800	0.00509500	7.03579400
C	-1.49384200	-0.10624100	6.88317100
H	-2.16599900	-0.97546600	8.73128800
Br	3.20994500	0.15845800	4.86838400
Br	0.73216500	1.89733000	3.44209700
N	6.27651500	-1.79246500	1.96141400
C	5.70201900	-1.57648900	3.20541100
H	5.94600900	-2.17451900	4.08955200
N	4.83310900	-0.57652400	3.16687100
C	4.82008100	-0.11352700	1.85699500
C	4.09218500	0.98513200	1.24704700
O	3.32301300	1.78420600	1.75688000
N	4.44626100	1.07373300	-0.17678200
C	5.31817000	0.22431600	-0.86660600
N	5.56980200	0.44302700	-2.19783000
H	6.27002800	-0.25060200	-2.48946800
N	5.97054900	-0.76471800	-0.23752500
C	5.72285100	-0.86935400	1.08073500
H	6.96769600	-2.50222500	1.71409500
Br	3.68604400	2.60044200	-1.04139700
Br	4.01828400	0.16644800	-3.50381100
N	0.98072600	-0.72618600	-6.86812300
C	2.23838300	-0.70733900	-6.28296900
H	3.13871100	-1.08079400	-6.78105300
N	2.18970500	-0.18583000	-5.06481800
C	0.86465000	0.15292100	-4.82862800
C	0.24287700	0.77225700	-3.67432200
O	0.75315200	1.13801200	-2.63004700
N	-1.19648200	0.95784100	-3.93295000
C	-1.88052500	0.55455700	-5.08343700
N	-3.21272900	0.83811500	-5.25072200
H	-3.49331100	0.47207400	-6.16806200
N	-1.24322900	-0.03931600	-6.10727100
C	0.08499300	-0.17923600	-5.95368100
H	0.73742500	-1.07564300	-7.79578900
Br	-4.61061800	0.20554100	-3.93950800
Br	-2.09493700	1.89951600	-2.52791200
N	-7.72195100	-1.64996400	-1.01675800
C	-7.14111200	-1.45937100	-2.26198400
H	-7.39915400	-2.05890500	-3.14108800
N	-6.24812500	-0.48053000	-2.23132300
C	-6.22527400	-0.00603600	-0.92572600
C	-5.47250800	1.08095100	-0.32520800
O	-4.68384700	1.85685000	-0.84091600
N	-5.82665900	1.19161800	1.09713900
C	-6.71845600	0.36904600	1.79393800
N	-6.96510500	0.60544600	3.12303200
H	-7.68111500	-0.06910400	3.42103400
N	-7.39347700	-0.61012500	1.17323900
C	-7.14702500	-0.73262400	-0.14376700
H	-8.43179300	-2.33919700	-0.76424500

Br -5.03225500   2.70863400   1.94804500
Br -5.41945900   0.30606700   4.43125600
64
Br-G4-3
H   -1.87997609354591   -0.37412939615394   8.47812980927515
C   -0.85681794837346   0.05271425400987   6.63925391266126
N   0.45023935492721   -0.16076679266337   6.90290822770171
H   2.69491124734985   -0.65680624152903   6.98818045171527
Br 3.84965305969159   1.21684705790380   5.96603210899281
N   2.60122459370365   -0.33770987573829   6.01335771157721
C   1.25441061955203   -0.00240546825118   5.84867743361327
Br 1.99751255826027   0.32772239716453   3.02084775382560
N   0.81423346803210   0.38434022715427   4.59479921263154
O   -0.84649582928493   1.11683101942122   3.15249951608949
C   -0.54850992815302   0.72556656721299   4.29092815889694
C   -1.41898744871977   0.51191376106017   5.42606330273663
N   -2.78749534561392   0.67479195250370   5.54518373364081
H   -4.06357267951101   0.32132070100288   7.24391483063204
C   -3.06537262886469   0.32178385992392   6.78925632824530
N   -1.93091414906879   -0.06538874216739   7.50625586087653
H   8.47837004321961   1.72193576016768   1.87983635113709
C   6.63916734872773   1.29678471291452   0.85656315117772
N   6.90316242264918   1.50981515572851   -0.45049766613819
H   6.98911950818965   2.00546593900207   -2.69525197034285
Br 5.96540323842512   0.13248744714346   -3.84971182155524
N   6.01398831325501   1.68729851289053   -2.60161012075792
C   5.84884972793082   1.35240465710225   -1.25474644602084
Br 3.02083983823939   1.02469369447871   -1.99819453949342
N   4.59453625739398   0.96700818685019   -0.81463625877558
O   3.15123631391282   0.23651082114955   0.84609683527747
C   4.29014036497497   0.62641664162164   0.54815130798717
C   5.42543966581115   0.83895450349963   1.41869087747969
N   5.54426566363695   0.67615623228618   2.78723280280597
H   7.24325056852076   1.02799705746240   4.06341391756248
C   6.78868553757593   1.02788975991095   3.06517132177034
N   7.50619319352451   1.41413827805816   1.93072242140627
H   1.88023176984726   -0.37410916821154   -8.47800796336986
C   0.85696109501597   0.05263598778438   -6.63917101463563
N   -0.45005158238387   -0.16110477973949   -6.90283760331283
H   -2.69463457343643   -0.65757932129274   -6.98809255044033
Br -3.84970831109630   1.21584190388163   -5.96600867788352
N   -2.60100884353003   -0.33843757184755   -6.01327942804107
C   -1.25426595747147   -0.00284207178303   -5.84862367209767
Br -1.99751847281310   0.32741631040666   -3.02088337859980
N   -0.81418741492960   0.38407365688641   -4.59476844550543
O   0.84637128128817   1.11698989625749   -3.15248102193664
C   0.54848364965193   0.72557335322333   -4.29088600383330
C   1.41902114995869   0.51201280994448   -5.42599537815814
N   2.78749560548670   0.67516471843986   -5.54510908151889
H   4.06367133450994   0.32185313558974   -7.24379821793432
C   3.06546299425793   0.32214032087558   -6.78915766719436
N   1.93109459192196   -0.06530840426818   -7.50614921686072
N   -7.50628714828136   1.41379653382621   -1.93081845582195
C   -6.78875774576233   1.02750407857662   -3.06523861220324
H   -7.24331640602821   1.02752974775757   -4.06348403957154
N   -5.54432565202265   0.67583464917107   -2.78727107348443
C   -5.42551845220715   0.83871180197885   -1.41873728792198
C   -4.29022513118362   0.62625151248700   -0.54817028027339

O -3.15129995600455 0.23637756583592 -0.84607783636885
N -4.59464243968377 0.96690519739714 0.81459059972023
Br -3.02099528196613 1.02459946500326 1.99816098017137
C -5.84896105343260 1.35231639644426 1.25466614544459
N -6.01408779896711 1.68734948674531 2.60149397976186
Br -5.96543980426507 0.13270899155714 3.84970602860453
H -6.98923443090542 2.00547330418468 2.69511283439732
N -6.90327744279296 1.50963766322402 0.45040106746791
C -6.63926591888493 1.29653492122246 -0.85664535782839
H -8.47847451025873 1.72156526732088 -1.87995788740309
64
Br-G4-4
N -1.96205500 0.25376400 7.36866800
C -2.96937800 -0.58810700 6.90684800
H -3.87672100 -0.79034900 7.48671200
N -2.69561200 -1.07498300 5.70502100
C -1.46540600 -0.52796300 5.35043200
C -0.66948400 -0.68382000 4.15360100
O -0.82921700 -1.45398000 3.21200700
N 0.40607600 0.30450600 4.13287000
Br 1.15482900 0.57861300 2.37852400
C 0.81296100 1.07293600 5.22165100
N 1.84159200 1.97958200 5.12677400
Br 3.60667300 1.66013100 4.28668800
H 1.98530300 2.42147800 6.04102800
N 0.14356300 1.04507200 6.38700000
C -0.99499000 0.32434900 6.37205300
H -1.93321200 0.75269500 8.25885300
N 7.33269700 -0.24411300 1.99825600
C 6.88046200 0.62205700 2.98910700
H 7.46317200 0.83574200 3.89200700
N 5.68347100 1.11584000 2.70723100
C 5.32172400 0.54851400 1.48795900
C 4.12712000 0.70128200 0.68827300
O 3.19233300 1.48332100 0.83321900
N 4.09706600 -0.30386600 -0.37103600
Br 2.33951000 -0.57149500 -1.11552800
C 5.17680100 -1.09382100 -0.76073700
N 5.07328600 -2.02054800 -1.76998400
Br 4.24270800 -1.73365000 -3.54452200
H 5.98226400 -2.47710100 -1.90067400
N 6.34172800 -1.06600700 -0.09036900
C 6.33447000 -0.32327200 1.03364200
H 8.21789500 -0.75226900 1.97806600
N 1.97926500 0.09262700 -7.32388700
C 2.96962300 -0.76316600 -6.85122100
H 3.87381400 -0.98890800 -7.42730300
N 2.68514500 -1.23142400 -5.64454000
C 1.46499800 -0.65708000 -5.29785100
C 0.66483100 -0.78381200 -4.10046900
O 0.80964000 -1.54517800 -3.14927000
N -0.39319000 0.22368500 -4.09295700
Br -1.13428100 0.53489900 -2.34110000
C -0.78278200 0.98849700 -5.19072400
N -1.79346800 1.91579400 -5.10793000
Br -3.56738700 1.63917600 -4.27241500
H -1.92500200 2.35208800 -6.02667800
N -0.11178200 0.93505900 -6.35423300

```
C   1.01250900   0.19284800  -6.32953000
H   1.96134000   0.58244800  -8.21938700
N  -7.32529600  -0.17282900  -1.96717400
C  -6.86330200   0.66774700  -2.97551000
H  -7.44195100   0.86669000  -3.88436600
N  -5.66295900   1.15766900  -2.70088300
C  -5.30950200   0.61420100  -1.46854200
C  -4.11441900   0.77351900  -0.67091100
O  -3.17214200   1.54255600  -0.83311800
N  -4.09495500  -0.20864800   0.41061900
Br  -2.33957300  -0.47736600   1.16063400
C  -5.18147100  -0.98279900   0.81337100
N  -5.08730300  -1.89155600   1.83955100
Br  -4.25479200  -1.58501200   3.60945200
H  -6.00006500  -2.33848500   1.97667400
N  -6.34524000  -0.95867600   0.14074000
C  -6.33018500  -0.23923700  -0.99832100
H  -8.21432700  -0.67384000  -1.93889500
64
Br-G4-5
  N   -11.28754854958778     -8.32723813982566     -1.11720745836102
  C   -11.71029220481986     -7.01258477223934     -0.93173904858666
  H   -12.72867257982669     -6.68402996271717     -1.16563556228053
  N   -10.74274778438720     -6.23955488071758     -0.46364021756719
  C   -9.63436023258948     -7.06638645635805     -0.32989172714916
  C   -8.28180148225127     -6.87080890939443      0.08073482607447
  O   -7.89588237029793     -5.67839590692513      0.49354826533390
  N   -7.43556793244620     -7.90577778503453      0.04079832275271
  C   -7.85636183680624     -9.13496455834271     -0.37372563587754
  N   -6.96857301016557    -10.17671075339312     -0.32706575756425
  H   -7.28535776924866    -11.02018527246366     -0.81169352956761
  N   -9.11741214261246     -9.43795519522269     -0.79857606428580
  C   -9.94810083376258     -8.39299968395341     -0.74338851964450
  H   -11.84168700926421     -9.10868269797835     -1.46867558280126
  Br  -5.84156952947095     -5.59790591462025      0.65959285556068
  Br  -5.03708100537818     -9.96506669729124     -0.33702996014529
  N   -0.35150624533670    -10.42737880851342     -1.12288848706335
  C   -1.66591200185972    -10.84998042185840     -0.93556644746420
  H   -1.99578224510971    -11.86729708014573     -1.17217788173754
  N   -2.43713211097786     -9.88356351885729     -0.46206091210826
  C   -1.60929606326972     -8.77618226674882     -0.32648359579066
  C   -1.80309942586450     -7.42481808031657      0.08886845522156
  O   -2.99488235368353     -7.03913832011377      0.50420272460094
  N   -0.76780400011286     -6.57899226721037      0.04931581522937
  C   0.45991733969251     -6.99909045375750     -0.37041113811953
  N   1.50221567828058     -6.11173770302042     -0.32365201574397
  H   2.34365365353780     -6.42757308939443     -0.81252064156600
  N   0.76104811445400     -8.25878547983403     -0.80029487304074
  C   -0.28396963701319     -9.08932281532194     -0.74450169345567
  H   0.42868065801174    -10.98065430912763     -1.47850135178858
  Br  -3.07762583562699     -4.98493419482353      0.66539536451511
  Br  1.28963326769591     -4.18002194306381     -0.33392825789947
  N   1.75088522997527      0.50438416838769     -1.12565742345151
  C   2.17373316707388     -0.80960999097712     -0.93624722815148
  H   3.19103600131019     -1.13973761665416     -1.17255276837126
  N   1.20757766190377     -1.58015244806486     -0.46108465000417
  C   0.10013404534298     -0.75225223401343     -0.32648749900146
  C   -1.25112118031427     -0.94564000850884      0.08952657570865
```

```
  O   -1.63639688438825     -2.13700738703960      0.50635354661645
  N   -2.09714579818189      0.08953205869657      0.04861387950631
  C   -1.67730694669453      1.31642957647375     -0.37374217816333
  N   -2.56471082706172      2.35879687539642     -0.32874070363425
  H   -2.24890091516723      3.19949765777754     -0.81886864192950
  N   -0.41783198039323      1.61709272127209     -0.80459809550472
  C    0.41291751961853      0.57237812432522     -0.74693231573675
  H    2.30393511406435      1.28406519809592     -1.48273154854336
  Br  -3.69090464403923     -2.22141899866209      0.66366919362638
  Br  -4.49630433547267      2.14770456007833     -0.33722686657309
  N   -9.18356399430771      2.61147830718279     -1.11860987378599
  C   -7.86925389293020      3.03438292000937     -0.93104598486657
  H   -7.54042799384679      4.05280849463474     -1.16435622168381
  N   -7.09685556188817      2.06689976530773     -0.46171393254083
  C   -7.92378254803613      0.95846018698474     -0.32927938205724
  C   -7.72867588924936     -0.39410820102378      0.08146153777610
  O   -6.53651875370862     -0.77998310582903      0.49540320178988
  N   -8.76352476137818     -1.24038989780090      0.04043591173531
  C   -9.99196746300285     -0.81982777967626     -0.37654002300647
  N  -11.03373522205873     -1.70766702830529     -0.33170457928405
  H  -11.87653566671208     -1.39074184977724     -0.81739332000200
  N  -10.29443462713657      0.44112450408161     -0.80205313870779
  C   -9.24975886922179      1.27202841425475     -0.74487148656096
  H   -9.96449805832245      3.16548704258955     -1.47141595393269
  Br  -6.45425828424064     -2.83418333170040      0.65788737606700
  Br -10.82312315343814     -3.63908336693077     -0.34050967701271
64
I-G4-1
  N  -11.41268981214231     -8.40795733332876     -1.15740488312835
  C  -11.84376232302640     -7.10319106000543     -0.95343962442400
  H  -12.85208533626069     -6.76139808951955     -1.20758807067021
  N  -10.88036247496396     -6.34466502680613     -0.44295576709675
  C   -9.77262139088292     -7.17464419552022     -0.30147983332722
  C   -8.43457760283443     -6.98124473305850      0.15357661338691
  O   -8.04009064567051     -5.82888868951839      0.62765727384227
  N   -7.58175997175047     -8.01677580191950      0.07457597415345
  C   -7.97656721105347     -9.24052514249588     -0.40141881696769
  N   -7.06508584575992    -10.24540168978502     -0.42953585373955
  H   -7.40980174180996    -11.09550505922086     -0.88298692737230
  N   -9.24644143955067     -9.52744743229336     -0.83557554624445
  C  -10.07610719507258     -8.48547065059800     -0.76126857944736
  H  -11.95928885487065     -9.17866191828805     -1.54293132952368
  I   -5.77004996999388     -5.84007828726405      0.80751191990037
  I   -4.89938504925808    -10.05666245122950     -0.35512593568451
  N   -0.26763913464292    -10.55505672599497     -1.15687285835193
  C   -1.57253961012582    -10.98612779698233     -0.95365193318976
  H   -1.91421238458255    -11.99440987745855     -1.20812732473472
  N   -2.33127016658593    -10.02281631607066     -0.44330761677303
  C   -1.50130291835092     -8.91517890281690     -0.30115858525308
  C   -1.69504311422766     -7.57717450235408      0.15383815880834
  O   -2.84796180819143     -7.18300684755108      0.62681256591681
  N   -0.65948962949627     -6.72432302753166      0.07578207878887
  C    0.56450010248872     -7.11903049490023     -0.39982411285242
  N    1.56943305122764     -6.20756877595331     -0.42711940772979
  H    2.41971425192352     -6.55226768078147     -0.88025966023724
  N    0.85163302239117     -8.38877064832606     -0.83416966660544
  C   -0.19030434849875     -9.21855521573899     -0.76039806221226
  H    0.50322056071060    -11.10160138480316     -1.54216459272896
```

I	-2.83678261700468	-4.91277229499589	0.80693675755442
I	1.38055892823686	-4.04181198886841	-0.35323826184004
N	1.87894222178919	0.58967999212006	-1.15612904918983
C	2.30996802198334	-0.71516607451270	-0.95242724500298
H	3.31832510759474	-1.05691569887683	-1.20649685678728
N	1.34649151123407	-1.47376385497598	-0.44218910288773
C	0.23882829516696	-0.64375820057768	-0.30056298842438
C	-1.09933986766764	-0.83743038877456	0.15395561336062
O	-1.49380576546866	-1.99027749861242	0.62684666198366
N	-1.95212429924668	0.19813721512507	0.07556890285132
C	-1.55729154871122	1.42203537988096	-0.40019962410462
N	-2.46872927776307	2.42694942491886	-0.42767781109494
H	-2.12405699331000	3.27717935675563	-0.88093312657528
N	-0.28744992738766	1.70903316383420	-0.83435271317403
C	0.54232983083125	0.66712303920704	-0.76005526233673
H	2.42559570205295	1.36044706094972	-1.54145288804134
I	-3.76410120843501	-1.97907298040624	0.80588450569905
I	-4.63451907549761	2.23796614205732	-0.35349106555360
N	-9.26589112355954	2.73644841243434	-1.15599403346127
C	-7.96096926861551	3.16725084074876	-0.95243339741729
H	-7.61907757090403	4.17556543400176	-1.20648016229967
N	-7.20248663255331	2.20365930090788	-0.44225675384675
C	-8.03263508395198	1.09610564192767	-0.30049962339182
C	-7.83931556607090	-0.24208780430236	0.15409831043633
O	-6.68664311442961	-0.63682263212644	0.62731978577890
N	-8.87501706315682	-1.09466946403070	0.07562736266648
C	-10.09886772437965	-0.69956489429874	-0.39993667182173
N	-11.10386956223638	-1.61091238683634	-0.42757143923758
H	-11.95414593367216	-1.26612919652257	-0.88065265728438
N	-10.38567626215226	0.57036278822675	-0.83397075961053
C	-9.34352973428296	1.39985307243520	-0.75988055472527
H	-10.03662750736536	3.28324175226766	-1.54118137396191
I	-6.69757884154207	-2.90678377784542	0.80690272834043
I	-10.91476605066303	-3.77666413112085	-0.35387880310253

64

I-G4-2

N	-2.21203700	-1.59462400	7.78016600
C	-3.44699900	-1.44292000	7.17289700
H	-4.32519000	-2.04417600	7.42769600
N	-3.40577300	-0.49196100	6.24588100
C	-2.10361600	0.00003100	6.23074300
C	-1.49854200	1.08117100	5.47590800
O	-2.03614800	1.84364300	4.67926800
N	-0.09527900	1.21332400	5.82269300
C	0.60731900	0.40611500	6.72960700
N	1.93835800	0.61364700	6.92454600
H	2.23472800	-0.01861500	7.67922300
N	-0.02473500	-0.54844500	7.44630600
C	-1.33308900	-0.68883400	7.18938700
H	-1.96757200	-2.25670500	8.51806100
I	3.39217400	0.23297800	5.23051500
I	0.78779100	2.94502200	4.91603400
N	6.72185300	-0.92599500	2.20837600
C	6.14775000	-0.83534800	3.46512000
H	6.61343400	-1.22513800	4.37511400
N	4.96962200	-0.22252000	3.40142600
C	4.75329900	0.10123200	2.06617700
C	3.66289600	0.80476900	1.42357200

O	2.66598500	1.28568000	1.94875000
N	3.90475200	0.92178600	-0.00681800
C	5.01020100	0.39743000	-0.69183500
N	5.15967400	0.57489500	-2.03236500
H	6.06302400	0.16243600	-2.29419900
N	6.00343900	-0.24339700	-0.03088800
C	5.84792700	-0.33560000	1.29574700
H	7.62160800	-1.34759800	1.97503500
I	2.39039100	2.10899600	-0.96408400
I	3.69363800	-0.01614200	-3.61727000
N	0.76643300	-1.68357200	-6.84355900
C	2.00489200	-1.55820500	-6.23742300
H	2.86823200	-2.18201800	-6.48896400
N	1.98710900	-0.60099400	-5.31607300
C	0.69714100	-0.07766400	-5.30351000
C	0.11864100	1.02240100	-4.55530300
O	0.67460600	1.77664900	-3.76336000
N	-1.28133000	1.18574100	-4.90179800
C	-2.00363700	0.39001900	-5.80343900
N	-3.32961900	0.62809000	-5.99830200
H	-3.64176100	-0.00115200	-6.74916300
N	-1.39499200	-0.58340600	-6.51487800
C	-0.09019400	-0.75348500	-6.25778500
H	0.50575200	-2.34409000	-7.57728900
I	-4.78959400	0.29070100	-4.30048100
I	-2.12215000	2.94347500	-4.00534600
N	-8.14302700	-0.77229400	-1.26940600
C	-7.56676500	-0.70487000	-2.52662100
H	-8.03944300	-1.09450900	-3.43307300
N	-6.37709400	-0.11421100	-2.46817700
C	-6.15513200	0.21819200	-1.13599600
C	-5.05129400	0.90620300	-0.49949800
O	-4.04496800	1.36245600	-1.02866800
N	-5.29118000	1.04109900	0.92967300
C	-6.40708700	0.54538800	1.61898300
N	-6.55314000	0.73877700	2.95770900
H	-7.46455000	0.34652800	3.22278300
N	-7.41244300	-0.08202500	0.96360700
C	-7.25825600	-0.19003000	-0.36199000
H	-9.05061900	-1.17465700	-1.03255800
I	-3.75272500	2.20480000	1.87704800
I	-5.09957300	0.12568500	4.54646600

64

I-G4-3

N	-1.69998400	0.55782300	7.58585300
C	-2.67347600	-0.41167300	7.30332200
H	-3.49386600	-0.61662500	8.00262900
N	-2.48082800	-1.01313000	6.14489600
C	-1.33294100	-0.42229300	5.62858000
C	-0.63217900	-0.62740700	4.39962500
O	-0.94084400	-1.46664900	3.49584000
N	0.47387900	0.22507400	4.20630500
I	1.21138600	0.07973200	2.05013300
C	0.90627400	1.14630100	5.15693300
N	1.93174800	2.01802300	4.90300800
I	3.93122800	1.57275100	4.14516600
H	2.03612300	2.62039100	5.72726900
N	0.27477600	1.30955900	6.34290500

```
C  -0.82590700   0.56537200   6.51133300
H  -1.62503100   1.14490700   8.41752000
N   7.46586000  -0.80648800   2.17551100
C   7.15515700   0.15180700   3.13302500
H   7.69770200   0.23974400   4.08026800
N   6.11928800   0.89484900   2.76190700
C   5.72713500   0.40549800   1.51808000
C   4.61124300   0.77388200   0.66896700
O   3.85729200   1.73299200   0.76502800
N   4.38988300  -0.25810800  -0.33691600
I   2.29490400  -0.30659700  -0.94274500
C   5.31846300  -1.24490900  -0.68963800
N   5.08781000  -2.06345300  -1.75132100
I   4.23976300  -1.43269900  -3.68293300
H   5.86481200  -2.72499600  -1.85568800
N   6.44069600  -1.43606500   0.03295000
C   6.53954600  -0.68204900   1.14407800
H   8.21011100  -1.50402900   2.22180500
N   2.32767000   0.73123100  -7.41174500
C   3.33704100   0.00067100  -6.81349500
H   4.33789300  -0.10583900  -7.24430800
N   2.93179500  -0.52884500  -5.66189500
C   1.60958700  -0.13148600  -5.48632400
C   0.65812000  -0.38848800  -4.41392200
O   0.83775400  -1.09266500  -3.41773400
N  -0.56556200   0.33159400  -4.61162100
I  -1.87294400   0.31396800  -2.78299700
C  -0.85041900   1.04403700  -5.75247400
N  -2.07619400   1.70469000  -5.83956300
I  -3.83547400   0.42097100  -6.12385200
H  -2.05362200   2.23047700  -6.72606400
N   0.00455800   1.22103600  -6.77775800
C   1.21421400   0.66832400  -6.57684300
H   2.37361200   1.22593900  -8.30396100
N  -7.47904000  -0.26024500  -2.02597300
C  -6.68870900   0.22789000  -3.06840400
H  -7.11019700   0.45532400  -4.05510400
N  -5.42130700   0.38075500  -2.72206600
C  -5.36149100  -0.02410000  -1.39893800
C  -4.25834200  -0.09344400  -0.47525500
O  -3.05303000   0.21901600  -0.68903100
N  -4.61110200  -0.59925500   0.78949200
I  -2.85653700  -1.04806700   2.13672600
C  -5.91071600  -0.90815400   1.14014200
N  -6.14327300  -1.43374200   2.40908500
I  -6.00041000  -0.02189200   4.10017000
H  -7.14393800  -1.67971000   2.43457600
N  -6.96214500  -0.82186900   0.30835100
C  -6.63930000  -0.42737100  -0.93627300
H  -8.48110000  -0.45626400  -2.03917400
64
I-G4-4
  H   -1.87862595694565     -1.02364894990668      8.64159971410346
  C   -0.88882095534368     -0.56407342176532      6.79169962401758
  N   0.41499583739163     -0.81091325960145      7.01089962811194
  H   2.70545791532290     -0.89457495996010      7.13896535721571
  I   3.52202220516864     -2.49161350804380      5.25603161649239
  N   2.58314312033609     -0.68098873694713      6.13715409586660
```

C	1.20505776761667	-0.56098369431933	5.95287873554912
I	2.09758512115827	0.27469098943261	2.97855468474348
N	0.74669683220829	-0.11643943117280	4.72769628179259
O	-0.93778700373878	0.55590117847012	3.31919543578329
C	-0.61596629042146	0.12593773109798	4.45990767213694
C	-1.48338588596686	-0.13031329858718	5.58017719116633
N	-2.85646571583807	-0.01478110774962	5.71559395672875
H	-4.09606858984537	-0.40641944470245	7.43344687050940
C	-3.10436383837666	-0.36746002925771	6.96646542916223
N	-1.94877071278964	-0.70764101972847	7.67327932992904
H	8.64120144070808	-1.02616159563354	1.87838416881520
C	6.79141899076179	-0.56577210776500	0.88873564643037
N	7.01035888650487	-0.81285320055609	-0.41507866963259
H	7.13811944394640	-0.89680056113038	-2.70563856271425
I	5.25425436576463	-2.49318953788556	-3.52161698826753
N	6.13640562454749	-0.68279010092099	-2.58315025380606
C	5.95237753928099	-0.56249393874391	-1.20505025324711
I	2.97835618329075	0.27438701885462	-2.09737469689988
N	4.72746871812211	-0.11727690654450	-0.74660781551421
O	3.31949623560765	0.55599467940619	0.93794186163667
C	4.45994886684020	0.12538837444828	0.61605588088816
C	5.58017359747426	-0.13135454333144	1.48339056368486
N	5.71581371935557	-0.01570747301979	2.85644007066107
H	7.43360822589037	-0.40806613877647	4.09589238121115
C	6.96653818032026	-0.36898449227956	3.10423464309844
N	7.67303817220851	-0.70969007102970	1.94860447677884
H	1.87857742127368	-1.02367499097358	-8.64159724383506
C	0.88878607912649	-0.56407855535649	-6.79169533794804
N	-0.41503085991785	-0.81093068018206	-7.01087957159729
H	-2.70549133227198	-0.89461387130137	-7.13891866243969
I	-3.52201631457332	-2.49162236876032	-5.25595449857928
N	-2.58317090920634	-0.68100687977556	-6.13711367915094
C	-1.20508475935865	-0.56098940059501	-5.95285544703434
I	-2.09758925427164	0.27470883449707	-2.97853188802882
N	-0.74671578117322	-0.11642307074250	-4.72768411581943
O	0.93777640609993	0.55594960283565	-3.31920828689357
C	0.61594788683899	0.12596827337893	-4.45991165668378
C	1.48335901006617	-0.13029403397331	-5.58018530366334
N	2.85643722804652	-0.01475567787021	-5.71561549475454
H	4.09602775590811	-0.40642086593893	-7.43347133632592
C	3.10432665709427	-0.36745758844176	-6.96648256813634
N	1.94872893670003	-0.70765266384200	-7.67328202577083
N	-7.67301188435365	-0.70965986916922	-1.94862106873765
C	-6.96650631980305	-0.36895505454689	-3.10424823601806
H	-7.43357496839863	-0.40802870588678	-4.09590695836350
N	-5.71578001203982	-0.01568769742942	-2.85644919551073
C	-5.58014472964345	-0.13134138436541	-1.48339982780775
C	-4.45991977195745	0.12539013295756	-0.61606249207569
O	-3.31946711707954	0.55599648221325	-0.93794711826246
N	-4.72744112664866	-0.11728665121123	0.74659915082241
I	-2.97832850090705	0.27436191717725	2.09737250029242
C	-5.95235300372613	-0.56250079651782	1.20503635589521
N	-6.13638348658804	-0.68280814591746	2.58313663269393
I	-5.25422631272304	-2.49321714440829	3.52161694877654
H	-7.13810684147248	-0.89679141990674	2.70564421376767
N	-7.01033558008770	-0.81284521229947	0.41506268087395
C	-6.79139295936433	-0.56575674634986	-0.88875010874043
H	-8.64117759614844	-1.02612461764882	-1.87840443737664

64
I-G4-5

H	-8.56427012839218	-0.39621712191788	-2.40530849754328
C	-6.79191432764999	-0.08362994397106	-1.23431837513663
N	-7.14518591628649	-0.35881597706659	0.03427262818930
H	-7.37248634488698	-0.96447154011116	2.24196212787136
I	-6.42304670765401	0.98389133609596	3.71035850778601
N	-6.38978802745226	-0.65349552732475	2.22573766703391
C	-6.13283245762159	-0.26806276672918	0.91226608617443
I	-3.11328813355076	-0.14731150973653	2.00730959214977
N	-4.84261921353149	0.09933000148056	0.58294422780672
O	-3.28348893403850	0.87576719955435	-0.91441365157078
C	-4.46979481933859	0.49369419307608	-0.71639811041221
C	-5.52813924225361	0.37218344978184	-1.68606299244295
N	-5.54984255177077	0.61770029356833	-3.04844404171205
H	-7.16317790437624	0.38794533634068	-4.45464917119767
C	-6.78011105782379	0.31688819386368	-3.42931838868911
N	-7.58355295349023	-0.11443384434052	-2.37142911260282
H	-2.02097109489746	1.98001832531380	8.28196050388220
C	-1.04447310920491	1.10270684500142	6.58310345407237
N	0.26718039614096	1.11062396631316	6.88138909233314
H	2.55170221359258	1.21879354625461	6.98607789928632
I	3.53988193128639	-1.13448299339506	6.43625733434178
N	2.42816519607062	0.74023083740372	6.08131229785385
C	1.05087244582737	0.62459573053182	5.90407868398950
I	1.94991209605841	-0.37968192548576	2.99146075359007
N	0.58204145820607	0.11579195786008	4.70864155280273
O	-1.12700837617252	-0.50079996459240	3.30360756548876
C	-0.78975867687989	0.01791874617740	4.40380843106449
C	-1.65087792394564	0.57017025125068	5.41775230284133
N	-3.02959165516183	0.68545163624080	5.48264275347757
H	-4.25925733508677	1.52613918700110	7.03793404366364
C	-3.26793326067160	1.27197854432339	6.64378908480510
N	-2.10126637057930	1.55196829812318	7.35863857489247
H	8.47376272563811	0.90572766761647	2.35066798324746
C	6.70696650136313	0.41504789206818	1.23291468724584
N	7.02037740118409	0.64318727227443	-0.05537464845353
H	7.16726017460267	1.16929220666368	-2.28356357428182
I	6.33144214159439	-0.86488142907673	-3.69689770738726
N	6.20938362206758	0.79094908607362	-2.23994723254820
C	6.00371683129036	0.43444794443826	-0.90928375135179
I	2.98585232479960	-0.00842306097049	-1.93286706396895
N	4.75114101933143	-0.01135850089874	-0.53592777792932
O	3.26543149489091	-0.78556042266225	1.03634453439691
C	4.42264683918676	-0.34292270220434	0.79246817322753
C	5.48280149664462	-0.09462717084674	1.73409896301659
N	5.54119026048094	-0.26009344381218	3.10802223935324
H	7.15918882969444	0.15432046100776	4.46652555148353
C	6.75505156900759	0.14053107611700	3.44684867243213
N	7.51244600333279	0.56215478950629	2.35103850495842
N	2.32767881390797	1.36975299425798	-7.43711628587994
C	3.41247539757367	0.93876233985580	-6.66977235064136
H	4.43519402053846	0.93605386584912	-7.06628127890320
N	3.05926385253650	0.54992447128031	-5.45597733780481
C	1.68752560448293	0.73221421224074	-5.40885239535564
C	0.73492253183145	0.50362243318765	-4.35328150248773
O	0.95915183853928	0.06352636358103	-3.19245693688847
N	-0.58596779933698	0.85341570469113	-4.69284643453003

```
  I   -2.00294782486374      0.86620180609331     -2.94094825286155
  C   -0.94518294450536      1.28537297760907     -5.95409245090154
  N   -2.27827462456042      1.62445061539230     -6.17666388311020
  I   -3.68035196717470     -0.07988314899880     -6.33310802505039
  H   -2.31559307774007      2.00048843319158     -7.13590045656698
  N   -0.08181858975173      1.48013904449603     -6.96477523324462
  C   1.20173820567594      1.24112003069157     -6.63936549073073
  H   2.33844811327234      1.70712143040121     -8.40059206257294
64
I-G4-6
  H   -1.89099702153744     -0.77353490105246      8.55978522939568
  C   -0.86417306580201     -0.21425731777259      6.75866317315160
  N   0.44547414556169     -0.36077172528732      7.03086656111835
  H   2.71490197433151     -0.72317724214975      7.10674171717440
  I   3.90620662032815      1.43808581364065      6.23721266865071
  N   2.61045173945878     -0.35146828501435      6.15076937274839
  C   1.24969137131191     -0.10223840955674      5.98616804519270
  I   2.14506450482356      0.35681627674305      2.93088511096904
  N   0.80918555485989      0.31007987423169      4.74434239031243
  O   -0.84724475908385      1.01440694391855      3.32057295826576
  C   -0.54531262443100      0.57480172835686      4.46387354092732
  C   -1.43517286638798      0.26551949611571      5.55332215341240
  N   -2.81358448012718      0.34317819409690      5.65614989934922
  H   -4.09164929302510     -0.15657816531659      7.31534601900842
  C   -3.08828168146131     -0.07887702900739      6.87918444238985
  N   -1.94444895923251     -0.43188677798560      7.59920433239963
  H   8.55969607681678      2.12333915238852      1.89112423320161
  C   6.75865499516134      1.56394721002563      0.86422024808963
  N   7.03092562840960      1.71036291054643     -0.44542403955313
  H   7.10695710973376      2.07264684254494     -2.71482972128510
  I   6.23763698880838     -0.08870955104645     -3.90609480888926
  N   6.15096633717668      1.70095122022991     -2.61045656034554
  C   5.98627286488532      1.45177820621506     -1.24969193876859
  I   2.93099698428443      0.99282464146745     -2.14517344893700
  N   4.74442396591731      1.03948361911176     -0.80923143118641
  O   3.32057460271473      0.33532460278429      0.84717304767528
  C   4.46390453314927      0.77487296379455      0.54526570310312
  C   5.55328266306553      1.08419361835143      1.43517071913960
  N   5.65599704196142      1.00667526261767      2.81360393185342
  H   7.31510143214781      1.50654814381257      4.09174211432683
  C   6.87901296012495      1.42875518289648      3.08834926488172
  N   7.59911931196254      1.78167367815515      1.94453621285223
  H   1.89088051106290     -0.77367800972797     -8.55980769448981
  C   0.86410734622104     -0.21425737726401     -6.75870309234518
  N   -0.44554852696644     -0.36066915384082     -7.03089935580032
  H   -2.71499663712982     -0.72290960187925     -7.10676530385582
  I   -3.90610144502794      1.43856091042642     -6.23746917672496
  N   -2.61053699710601     -0.35113173097298     -6.15081917056869
  C   -1.24975707455997     -0.10201711218456     -5.98621961826515
  I   -2.14511337362769      0.35718593712102     -2.93093050850423
  N   -0.80921694306561      0.31030160034489     -4.74440744117354
  O   0.84726653268178      1.01452115993575     -3.32066940631278
  C   0.54531009329561      0.57490786754635     -4.46396109834837
  C   1.43514326252168      0.26553450505719     -5.55338453418056
  N   2.81356456608185      0.34307497287732     -5.65621382756251
  H   4.09159015513345     -0.15686703009550     -7.31538357485741
  C   3.08822722507140     -0.07905764132167     -6.87923037110782
  N   1.94436259204349     -0.43200349157441     -7.59923807421930
```

N	-7.59913066162917	1.78226581566199	-1.94442418002516
C	-6.87910506374770	1.42926608923124	-3.08825903295988
H	-7.31524932568399	1.50701461826525	-4.09163081310218
N	-5.65609595076739	1.00714095553697	-2.81355451918871
C	-5.55328136047215	1.08474899098562	-1.43513762175926
C	-4.46386490761521	0.77540920623758	-0.54526775250270
O	-3.32056985691075	0.33579416653828	-0.84719221711416
N	-4.74435742013273	1.04004252506353	0.80924904645725
I	-2.93097247365059	0.99308677628452	2.14521244749546
C	-5.98617269708887	1.45241010967742	1.24973197604142
N	-6.15078989888644	1.70162868534797	2.61049047886179
I	-6.23731473400513	-0.08794557786976	3.90622994746398
H	-7.10676361295715	2.07334906544259	2.71490865038125
N	-7.03083309491840	1.71102530942086	0.44549375152402
C	-6.75861492740877	1.56455568256349	-0.86414691124489
H	-8.55969895666223	2.12394859930869	-1.89097514263613

64

At-G4-1

N	-11.50133465428265	-8.48573508691773	-1.13318923824309
C	-11.94050211011281	-7.18173247093625	-0.94922364589096
H	-12.95268467009173	-6.84999433607173	-1.20015889675437
N	-10.97471076200776	-6.40938841861214	-0.45929812342785
C	-9.86083184446275	-7.23110804811118	-0.31225483528600
C	-8.52193875685576	-7.01063550600761	0.12910384909959
O	-8.12620022385501	-5.85785384186696	0.57562046273079
N	-7.66001347303213	-8.04667037166559	0.05617778809805
C	-8.04795648144740	-9.28786249335892	-0.39133254834270
N	-7.13453393587708	-10.28441318746735	-0.40942305671925
H	-7.49744524384133	-11.14392239115585	-0.83057250114581
N	-9.32476557928542	-9.58671449322882	-0.80808984632163
C	-10.15928561521255	-8.54929667617099	-0.74711621118834
H	-12.04709356720893	-9.26564311820823	-1.50093781539853
At	-5.72205201826370	-5.95662736752035	0.73566195151380
At	-4.84881457457701	-10.11857880319941	-0.35164359282435
N	-0.19051421050139	-10.64425632142002	-1.13280063994211
C	-1.49469039204972	-11.08306411756227	-0.94923858087038
H	-1.82664352456261	-12.09515716158843	-1.20024395525954
N	-2.26690613153315	-10.11704777864115	-0.45952833296175
C	-1.44492101408967	-9.00344692536299	-0.31210739272032
C	-1.66504629177346	-7.66439604587098	0.12896503309960
O	-2.81800705635247	-7.26847051163106	0.57510868814548
N	-0.62888582710127	-6.80272320614794	0.05632682784913
C	0.61229081829403	-7.19103775751382	-0.39095464110799
N	1.60898972698149	-6.27774693144995	-0.40892327553415
H	2.46834834658129	-6.64069130602165	-0.83037477336802
N	0.91092223520179	-8.46793798723516	-0.80752672281143
C	-0.12669199195935	-9.30223366477009	-0.74667476869249
H	0.58940844614556	-11.19027847163951	-1.50012911676057
At	-2.71962972487207	-4.86468336352003	0.73548262865616
At	1.44259895177837	-3.99183879954893	-0.35154085976768
N	1.96785154983707	0.66687609679238	-1.13321395429257
C	2.40663283208721	-0.63731983301836	-0.94961038915687
H	3.41861521297906	-0.96938949035101	-1.20090321974565
N	1.44064105753348	-1.40944242619340	-0.45970766881685
C	0.32712564025900	-0.58738530891503	-0.31213549877257
C	-1.01191568528677	-0.80748776256253	0.12895219071814
O	-1.40764189329259	-1.96039458321499	0.57541943817267
N	-1.87362708925822	0.22862968355771	0.05621743294884

```
  C   -1.48535311066534      1.46977160522204     -0.39117261456361
  N   -2.39860983870380      2.46651461583992     -0.40934439428564
  H   -2.03567736961467      3.32553502642766     -0.83152090800394
  N   -0.20839589953172      1.76841246959057     -0.80764940744389
  C   0.62588243559777      0.73079112699763     -0.74689976334236
  H   2.51375533598345      1.44666521931331     -1.50100147359820
  At  -3.81157088389390     -1.86237282211675      0.73587487552615
  At  -4.68477181730567      2.30035742571495     -0.35147811331504
  N   -9.34404487742623      2.82554145396821     -1.13278892239210
  C   -8.03993517336426      3.26446638195166     -0.94891996844671
  H   -7.70804830367111      4.27667117338709     -1.19955129618148
  N   -7.26769055524222      2.29831716423366     -0.45948064964770
  C   -8.08955784562973      1.18460374055302     -0.31257840394216
  C   -7.86924034999502     -0.15445557074423      0.12840575009552
  O   -6.71621885697661     -0.55019321910662      0.57443140386548
  N   -8.90538900352543     -1.01612986181685      0.05585393283643
  C   -10.14644977443928     -0.62798653550776     -0.39186146741759
  N   -11.14302384699752     -1.54142506354900     -0.41020445490416
  H   -12.00235205142642     -1.17858711692632     -0.83179700129045
  N   -10.44524434931769      0.64890991681827     -0.80835913217310
  C   -9.40775545651548      1.48336205465600     -0.74719256808145
  H   -10.12393533631829      3.37150473184139     -1.50027522453243
  At  -6.81389038761296     -2.95394182855805      0.73531540705747
  At  -10.97682015003948     -3.82720251186143     -0.35179979472745
64
At-G4-2
N  -2.48512700   0.76369600   8.51628800
C  -3.68096300   0.55242800   7.84702300
H  -4.65450600   0.84800200   8.24949700
N  -3.47336200  -0.01843700   6.66079100
C  -2.09668100  -0.19258400   6.54711800
C  -1.24841100  -0.68636100   5.50954100
O  -1.75289200  -1.20415300   4.42763000
N   0.08054600  -0.56923600   5.67473000
C   0.61740700  -0.04231200   6.82590600
N   1.96292100   0.02886100   6.92520100
H   2.27862200   0.50942300   7.77220000
N  -0.12697600   0.41163700   7.89516700
C  -1.44029500   0.31971500   7.69579400
H  -2.37250300   1.19157700   9.43583300
At   3.47879000  -0.08336200   5.20628100
At  -0.11823200  -1.12515600   2.68515100
N   7.22715900   0.46452000   2.34682500
C   6.57758000   0.29969000   3.56258000
H   7.06747800   0.45181600   4.52931300
N   5.30412400  -0.04149500   3.38846400
C   5.10410800  -0.10534900   2.01174700
C   3.97049200  -0.38634800   1.18730700
O   2.82048100  -0.71026200   1.68520200
N   4.13476400  -0.28930800  -0.15066000
C   5.34510300   0.03782700  -0.71538200
N   5.43213400   0.09063800  -2.06174200
H   6.34728400   0.39945600  -2.39895500
N   6.48358200   0.30617600   0.00813100
C   6.30347600   0.22249100   1.32623600
H   8.20034200   0.73855900   2.20889200
At   1.21999300  -0.74634200  -0.15636500
At   3.73941300   0.01808500  -3.59347500
```

```
N   1.09314800   0.32427000  -7.51013200
C   2.25722100   0.27075000  -6.75757300
H   3.25432500   0.42147600  -7.18287300
N   1.99521100   0.02796300  -5.47678800
C   0.61078500  -0.08333300  -5.37647900
C  -0.29766600  -0.32136600  -4.29970900
O   0.13817900  -0.52012200  -3.08491000
N  -1.61261900  -0.33199300  -4.57350200
C  -2.09161700  -0.13219200  -5.85177600
N  -3.42370900  -0.17107500  -6.05464100
H  -3.69463500   0.04292900  -7.01776300
N  -1.28601400   0.09670600  -6.94286300
C   0.01246300   0.10681500  -6.65155300
H   1.02244200   0.50986300  -8.51112900
At  -5.05240900  -0.18382200  -4.45591700
At  -1.67873600  -0.76877100  -1.58912300
N  -8.52131600   0.82400000  -1.39396900
C  -8.05374700   0.55298700  -2.67366200
H  -8.60246400   0.82330600  -3.58107800
N  -6.86271000  -0.03644000  -2.63569100
C  -6.53280500  -0.15887400  -1.28910500
C  -5.36601300  -0.68660700  -0.64080500
O  -4.42553800  -1.26421800  -1.23516200
N  -5.35803700  -0.48665900   0.75332900
C  -6.42352300   0.05192200   1.46668500
N  -6.42856500   0.13661100   2.81538600
H  -7.30361000   0.57618600   3.11534600
N  -7.55959800   0.50048700   0.84419500
C  -7.55226000   0.39303400  -0.48473900
H  -9.40548400   1.27127500  -1.15012900
At  -3.06006100  -0.93880500   1.48356800
At  -4.99337300  -0.03603600   4.62468900
64
At-G4-3
  N   -2.08204229971972      0.83882846612981      7.80707168859758
  C   -3.30807044987693      0.41766057509325      7.30543951624764
  H   -4.23763135702590      0.46525923250715      7.88137127948541
  N   -3.19299493809763     -0.01829343353050      6.05449247784913
  C   -1.84942956503560      0.11980063157020      5.72188481450574
  C   -1.14659210250149     -0.14286470555106      4.49484727151655
  O   -1.65279752474277     -0.66596378876821      3.48000336813778
  N   0.18921685432584      0.29992135306360      4.51714311012182
  At  0.90568248921391      0.40279886558484      2.15979531669055
  C   0.82378852995082      0.80974543804727      5.63925503767431
  N   2.11796553969430      1.21248269966573      5.57960799326532
  At  3.93329341030287      0.47700708253801      4.33214328196507
  H   2.41501034861637      1.55008863731218      6.50058385810519
  N   0.17516534215114      0.97696839492203      6.83027283086141
  C   -1.12897000290677      0.67482820101681      6.79995810914023
  H   -1.90381527114334      1.22370376026567      8.73533703709160
  N   7.84870652709880     -1.06556688591270      2.08802103773118
  C   7.11486276367011     -0.71067526700002      3.21055749232484
  H   7.48519765224101     -0.83554165485726      4.23276742631592
  N   5.92410728323643     -0.22065813903655      2.87268862560940
  C   5.87081985324405     -0.24945574854160      1.48032354792104
  C   4.86875589068613      0.08066401942700      0.51585719981752
  O   3.72676066904324      0.58171749051735      0.89668300385832
  N   5.13224084023829     -0.15364466308072     -0.77819724388017
```

At	2.09803900056327	0.41419159106042	-0.82463427837043
C	6.34235296239061	-0.66881168272651	-1.18451008219467
N	6.54554275768617	-0.84313573668718	-2.50726428792640
At	4.98984292593122	-0.57882679376408	-4.15796590116177
H	7.44276399003263	-1.27588208677696	-2.73949420218219
N	7.36326544646246	-1.01603135460556	-0.32535431142873
C	7.07376426119039	-0.79133162202678	0.95481886116430
H	8.78611923898532	-1.46848617768860	2.07804351622601
N	2.10754854135172	0.65821863282739	-7.78228617828709
C	3.32530449909502	0.22291457654145	-7.27236412952924
H	4.25568331637531	0.24226483665286	-7.84862740155557
N	3.20145590009580	-0.18826007231949	-6.01387399245422
C	1.86069193243562	-0.01875210794390	-5.68470613246452
C	1.15252630680687	-0.24587115286898	-4.45364159958231
O	1.64831050104221	-0.75932346505356	-3.42882427332603
N	-0.17463073516299	0.22166416545276	-4.48483270244301
At	-0.88763965799406	0.38170199720411	-2.12852612236438
C	-0.79916009473486	0.72273503400458	-5.61636819786099
N	-2.08591131119346	1.14975032251906	-5.56509037380686
At	-3.91310088590814	0.47170070105762	-4.30351133792133
H	-2.37632147812454	1.47688513667972	-6.49194939319604
N	-0.14697250587114	0.85656716052174	-6.80972066794070
C	1.15124179563154	0.53042242539349	-6.77304191214126
H	1.93730320571813	1.03085268269072	-8.71703551269338
N	-7.85597868005912	-0.95517553972948	-2.03113527906710
C	-7.11549050924292	-0.63551139762298	-3.15986619963487
H	-7.48738233961658	-0.77377101983243	-4.17975762650190
N	-5.91604727415945	-0.16099380457799	-2.83069194970502
C	-5.86370950104969	-0.16379076679922	-1.43805785948396
C	-4.85612612659067	0.16601549753523	-0.47931611479806
O	-3.70392907380862	0.63700378652805	-0.86825409536577
N	-5.12462448344988	-0.03802896647001	0.81877393925664
At	-2.07906918631458	0.46692331194050	0.85557800670699
C	-6.34413308953033	-0.52291822968917	1.23411459782324
N	-6.55135030179386	-0.66675847177390	2.55989394265841
At	-4.98966383373138	-0.40550216410505	4.20558035654173
H	-7.45494496845341	-1.08197488394528	2.79945281483630
N	-7.37066689275360	-0.86827405828172	0.38097507754810
C	-7.07658344332005	-0.67338664251224	-0.90294858051089
H	-8.80013269159415	-1.34179922219090	-2.01402249781643

64
At-G4-4

H	-1.91042811414333	-0.89353241724736	8.62726786088039
C	-0.86932964575626	-0.30897795920159	6.84140694828157
N	0.43700987312540	-0.44595451324972	7.12301995359203
H	2.71333490847310	-0.78477260860597	7.19962804891453
At	3.95289097151506	1.49203340505326	6.38635252668402
N	2.60598913957057	-0.41130126179329	6.24382829480359
C	1.24896770169752	-0.17042177371808	6.08199111464756
At	2.21580983348614	0.36312607429527	2.93868134748776
N	0.81300294077937	0.25319390815853	4.84261592049134
O	-0.83232889194442	0.95457731795363	3.41456640538158
C	-0.53451769463042	0.50325160454101	4.56020801916330
C	-1.43522084301787	0.17384412310868	5.63308602560486
N	-2.81530537875117	0.23264218073466	5.72149661787593
H	-4.10443304673269	-0.29457973780503	7.36375591598670
C	-3.09736567622110	-0.20189094884640	6.93928155733757
N	-1.95732717161121	-0.54575967494022	7.66857795194958

H	8.61882428980426	2.28603186947007	1.90980920608426
C	6.83644007970417	1.68957805428146	0.86951027882155
N	7.11672086015381	1.82811095233765	-0.43693602421522
H	7.19010730089698	2.16735436012438	-2.71337881123555
At	6.39081759050345	-0.11411222210169	-3.95369001736009
N	6.23699205678641	1.78726710246189	-2.60552189564772
C	6.07730238299181	1.54534168590916	-1.24852542754076
At	2.93736742975470	0.98994390027526	-2.21382074508064
N	4.84104283940214	1.11334586665380	-0.81193481278146
O	3.41854274570330	0.40187446905221	0.83379001554207
C	4.56087825627456	0.86140111546930	0.53570364812504
C	5.63164117415918	1.19863186766081	1.43593269007790
N	5.72086634486535	1.14090719230866	2.81601177699887
H	7.35984844788233	1.67992918929501	4.10447703875566
C	6.93571636836751	1.58391268609227	3.09757460128616
N	7.66229395408765	1.93243238281074	1.95718620129402
H	1.91536710192436	-0.92460718109534	-8.62161424690165
C	0.87265889357032	-0.33705439905786	-6.83770326242930
N	-0.43325389002013	-0.47924031229879	-7.11862164509147
H	-2.70842621383747	-0.82634838176865	-7.19330448767233
At	-3.95566362658514	1.44955506936047	-6.38959454061108
N	-2.60219915391210	-0.44845984148569	-6.23913105195434
C	-1.24597808642895	-0.20249084103473	-6.07847343348598
At	-2.21412322820850	0.34187859105788	-2.93723354033182
N	-0.81117706386585	0.22724566390054	-4.84079136639113
O	0.83222573124888	0.93866447481404	-3.41545264029283
C	0.53564295707767	0.48243121772044	-4.55942631175150
C	1.43713985719421	0.15183690574744	-5.63121214692594
N	2.81705624729296	0.21445152273787	-5.71984986478855
H	4.10767625996411	-0.31468080274476	-7.36037472305205
C	3.10037446789915	-0.22356472340160	-6.93610968258304
N	1.96134037642363	-0.57350765025666	-7.66410072985864
N	-7.66754439583512	1.89370751442572	-1.96197619048360
C	-6.93959832482557	1.54321327513293	-3.10087380845742
H	-7.36432655679131	1.63248982440258	-4.10814401979673
N	-5.72263557576319	1.10715477044526	-2.81753859886902
C	-5.63341129979381	1.17179631716387	-1.43776137750850
C	-4.56093867571748	0.84365643569192	-0.53619963625945
O	-3.41655543303967	0.38819934523428	-0.83258664540831
N	-4.84197643288255	1.10034840527575	0.81035014427010
At	-2.93766499274089	0.99116147818413	2.21270540179119
C	-6.08002128030040	1.52892785818159	1.24510485405009
N	-6.24043945062277	1.77633852974338	2.60113993270143
At	-6.38784604472269	-0.12037953096894	3.95657374594590
H	-7.19512438380342	2.15301875520026	2.70721247693105
N	-7.12091758291790	1.80325504413469	0.43249380880811
C	-6.84035135572060	1.65984713937671	-0.87337010386267
H	-8.62545987143639	2.24372733564302	-1.91608854193663

64

At-G4-5

N	-2.27167509618561	-0.94347885612501	8.37652090471248
C	-3.53374061270900	-0.90770154870713	7.80903318815026
H	-4.39594061686004	-1.44167936115658	8.21999248920629
N	-3.54001765890255	-0.16783904342753	6.70454971013496
C	-2.23600540790556	0.29861251867837	6.52873728346562
C	-1.65249650520859	1.19574333822418	5.55333753031498
O	-2.23730478150440	1.82913419685868	4.66754196961176
N	-0.23726018409182	1.32983334106809	5.74941365839091

C	0.50960104221502	0.72995525347472	6.77427359235483
N	1.84535142296943	0.93661806042091	6.85884731272231
H	2.16355883465343	0.47421027669159	7.71991693009687
N	-0.10179981984758	-0.02182211978283	7.72508464492898
C	-1.41853919606585	-0.19165879651848	7.56910339244691
H	-1.99518033624090	-1.43839082485605	9.22539048999007
At	3.37952957248328	0.25221084104211	5.18617441212507
At	0.64432957640029	2.78081834237613	4.24842538657949
N	6.70241602826550	-1.27921792727523	2.24837449368290
C	6.13578213306088	-1.09711997156724	3.49869696987801
H	6.58434796883367	-1.46087186443542	4.42759023698032
N	4.98731530602189	-0.43202643667598	3.40158592239297
C	4.78542924135191	-0.16716892017621	2.04967060547806
C	3.72454472681481	0.53028316216808	1.35806093043826
O	2.74118341824854	1.07766972761628	1.86059455483281
N	3.94105543765379	0.53958748510265	-0.06303475580348
C	5.04090995650819	-0.02609065902538	-0.72181007659983
N	5.23934597225997	0.09578925658153	-2.05723426759508
H	6.12910689480072	-0.36826637853994	-2.27209261381499
N	6.01807815242069	-0.66968308713630	-0.02233734409013
C	5.85676575478050	-0.69550157519893	1.30267383117606
H	7.58361582594080	-1.74992835381077	2.04012374962748
At	2.22742241791363	1.61369590339264	-1.11395979320055
At	3.84662459711551	-0.14467139491669	-3.91855889064166
N	0.84503613083605	-1.03756367263207	-7.44484994630995
C	2.10847397498486	-1.02545817027574	-6.87935574592738
H	2.95819345561391	-1.58088136785519	-7.28796602038895
N	2.13279601780418	-0.27837625209850	-5.78000667918183
C	0.83942013930841	0.21746891569681	-5.60558845087956
C	0.27684378858387	1.13287987423057	-4.63510073745525
O	0.87687044377588	1.75908362076183	-3.75419414427222
N	-1.13554345700931	1.29555307208583	-4.82889630952445
C	-1.89693754238801	0.70581724340709	-5.84894103958065
N	-3.22800060926600	0.94060597492443	-5.93373922938761
H	-3.55674292964982	0.47917258851115	-6.79135068140108
N	-1.30331803826755	-0.06470275882658	-6.79616223359737
C	0.00968580993902	-0.26196041238961	-6.64131419264722
H	0.55645987810325	-1.53252481094597	-8.28967043006603
At	-4.77909874762033	0.30724098069678	-4.25679694516788
At	-1.98302150331524	2.76958591754955	-3.33045606604033
N	-8.13530041763964	-1.12356680651628	-1.30577526823809
C	-7.56459933135876	-0.96620348338602	-2.55755134085206
H	-8.01977797951498	-1.33118580026671	-3.48275496273830
N	-6.40304100859636	-0.32330423318387	-2.46689027950051
C	-6.19644376698066	-0.04814083989119	-1.11769825957640
C	-5.12182493537418	0.63481055244281	-0.43272033516381
O	-4.12747179802942	1.15711039983615	-0.94028745356618
N	-5.33836001940904	0.66256504610067	0.98803674305780
C	-6.44955059447303	0.12596435147103	1.65208212886757
N	-6.64478075994892	0.26622800662686	2.98630064323447
H	-7.54407882361444	-0.17703639712592	3.20565865788185
N	-7.43945408772824	-0.50446853036987	0.95887124526331
C	-7.27839250501117	-0.54711171192291	-0.36580286362344
H	-9.02504545422866	-1.57576478346545	-1.09293574072207
At	-3.60279755164364	1.70968672146630	2.02969628126980
At	-5.25343584307262	-0.00240881902030	4.84587120826084

64
At-G4-6

H	-1.87857813151413	0.56241861580915	8.56086608867644
C	-0.86911900600843	-0.13530644754964	6.79749795120376
N	0.44209870253984	0.00738240598833	7.05052376449020
H	2.70782942374901	0.36193721964630	7.06779702673531
At	3.99207456426672	-1.93693926226870	6.43945273866062
N	2.59646847683425	-0.09012729446949	6.14721197013079
C	1.23885982826505	-0.35143385270940	6.02263634606737
At	2.17707354264497	-1.22936368282046	2.94620897518043
N	0.78165897360395	-0.86269023154076	4.82392365790245
O	-0.90079507236589	-1.56948821176854	3.43913305823846
C	-0.57528987376693	-1.07959218127902	4.56290993252394
C	-1.45703645505556	-0.68089568627340	5.62704213995852
N	-2.83670093607216	-0.72163077300423	5.73826862784959
H	-4.09566624630099	-0.08567437031182	7.36539218199806
C	-3.09625089654452	-0.21386333993003	6.93215661476165
N	-1.94252116736194	0.16187461913760	7.62402975586229
H	8.55379953279841	0.59274657084802	1.87481196532967
C	6.79293546231726	-0.11374177495150	0.86709287751960
N	7.04580577105994	0.02583784190833	-0.44448016102425
H	7.06238955653823	0.37397811586915	-2.71108411405659
At	6.44253245685067	-1.93039236702696	-3.98944309904171
N	6.14336845501639	-0.08100203678394	-2.59870986470002
C	6.01938206491083	-0.33900050191941	-1.24040762288445
At	2.94656813019656	-1.23200625274230	-2.17675271709361
N	4.82241120958067	-0.85323545786522	-0.78198372810892
O	3.43938041818341	-1.55906605289707	0.90229278143399
C	4.56166079411864	-1.06652590397779	0.57560773516687
C	5.62412770786332	-0.66137958343995	1.45640290205411
N	5.73506027979893	-0.69729948948166	2.83621671816695
H	7.35971175066714	-0.05211259685728	4.09363728992030
C	6.92721276932305	-0.18485311848629	3.09450364622130
N	7.61820122289254	0.18948306364866	1.93978184860638
H	1.87738426672052	0.56434592310922	-8.55969379210985
C	0.86942869554822	-0.13570337395660	-6.79639275774677
N	-0.44204011422136	0.00578699551733	-7.04874816387909
H	-2.70831841397819	0.35729371218122	-7.06498145225475
At	-3.98868252110178	-1.94434985494457	-6.43919239173851
N	-2.59604485302209	-0.09551525066207	-6.14486698570763
C	-1.23801323502201	-0.35503853916571	-6.02096568329793
At	-2.17431335215706	-1.23784774116165	-2.94533238368690
N	-0.77976720486791	-0.86675196193043	-4.82283011101550
O	0.90422555320659	-1.57226037168838	-3.43909377882945
C	0.57755849220761	-1.08209955082216	-4.56243249362975
C	1.45840327988425	-0.68175855565706	-5.62667233137974
N	2.83803807975412	-0.72127616525754	-5.73867061433346
H	4.09567687766561	-0.08276944658858	-7.36577763566803
C	3.09656289872343	-0.21220457439648	-6.93222365583041
N	1.94215563045622	0.16313743711081	-7.62319793684194
N	-7.62169193290191	0.15890121317269	-1.94147515190712
C	-6.92931266069479	-0.21439513597917	-3.09569229128392
H	-7.36236170119022	-0.08469175139208	-4.09499087572363
N	-5.73521358571575	-0.72195479437315	-2.83672876237066
C	-5.62433391875325	-0.68357824674780	-1.45697413673134
C	-4.56020943201777	-1.08322883909893	-0.57566450210452
O	-3.43599508849816	-1.57175482866024	-0.90166560250872
N	-4.82184839066924	-0.86909405807896	0.78164443412750
At	-2.94471846437119	-1.23819110683594	2.17718139376151
C	-6.02094771322151	-0.35932937160795	1.23941804765778

N	-6.14613059277464	-0.10071226216057	2.59750228305733
At	-6.43842295600302	-1.95070596659382	3.98903802629341
H	-7.06700059107298	0.35064719365964	2.70940491197340
N	-7.04876791825043	0.00051198296538	0.44299783097980
C	-6.79525814772079	-0.13967378855852	-0.86838279183722
H	-8.55869429496978	0.55898268810135	-1.87707793318348
